# Polymer-Supported Synthesis of Various Pteridinones and Pyrimidodiazepinones

**DOI:** 10.3390/molecules26061603

**Published:** 2021-03-14

**Authors:** Jan Chasák, Lucie Brulíková

**Affiliations:** Department of Organic Chemistry, Faculty of Science, Palacký University, 17. listopadu 12, 771 46 Olomouc, Czech Republic; jan.chasak01@upol.cz

**Keywords:** solid-phase, cyclization, dichloronitropyrimidine, pteridinones, pyrimidodiazepinones

## Abstract

In this report, we employed the solid-phase synthetic approach to prepare variously substituted dihydropteridinones, tetrahydropyrrolopteridinones, and pyrimidodiazepinones, using a versatile building block-4,6-dichloro-5-nitropyrimidine. All these compounds are pharmacologically significant scaffolds of the great importance of medicinal chemists. The fast and efficient synthetic methodology is highly desirable for defining their structure-activity relationship (SAR) and optimizing pharmacokinetic properties. Our research efforts utilize simple synthetic methods to generate a library of analogues for future SAR studies. The efficiency of our approach was exemplified in various pteridinones as well as pyrimidodiazepinones.

## 1. Introduction

**Figure 1:**
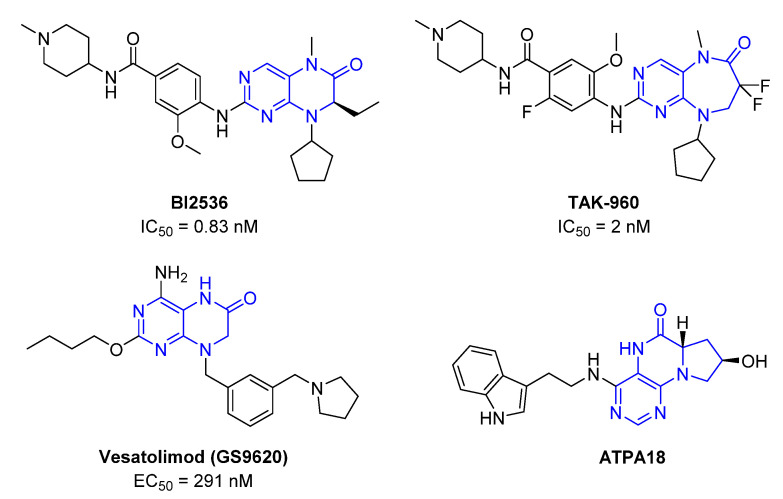
Representative examples of biologically active pteridinones and pyrimidodiazepinones.

Pteridinones and pyrimidodiazepinones represent an important group of heterocyclic compounds that have attracted enormous attention within medicinal research, especially in the last decade [1,2,3,4,5,6,7,8,9,10,11,12,13,14,15]. Many of them have been intensively studied as Polo-like kinases inhibitors (serine/threonine kinases playing a crucial role during mitosis, and their deregulation can be observed in many types of tumors) [2,3,4,5,7,8,10,11,12,15]. Of these compounds, dihydropteridinone BI-2536 [3,12,13,15] or pyrimidodiazepinone TAK-960 [16] (Figure 1) reached advanced clinical trials and have been taken into considerable attention due to their anticancer effects in any kind of tumors. Another example is 2-butoxy-7,8-dihydropteridin-6(5*H*)-one analogue GS9620 (Vesatolimod, Figure 1) discovered as Toll-like receptor agonists being currently under clinical evaluation for the treatment of HBV and HIV positive patients [17,18]. Interestingly, pyrrolopteridinone ATPA18 (Figure 1) was identified as a nontoxic, cell-permeable, and reversible inhibitor of the RNA interference pathway [14].

**Figure 2:**
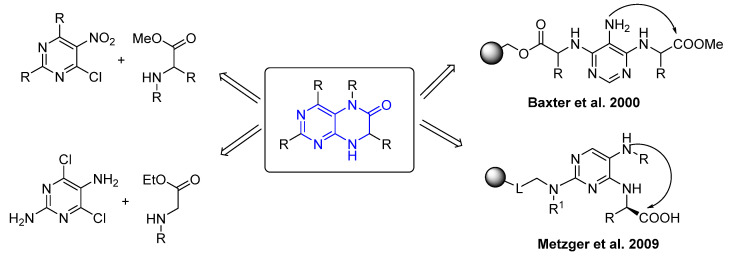
Overview of known methodologies.

**Figure 3:**
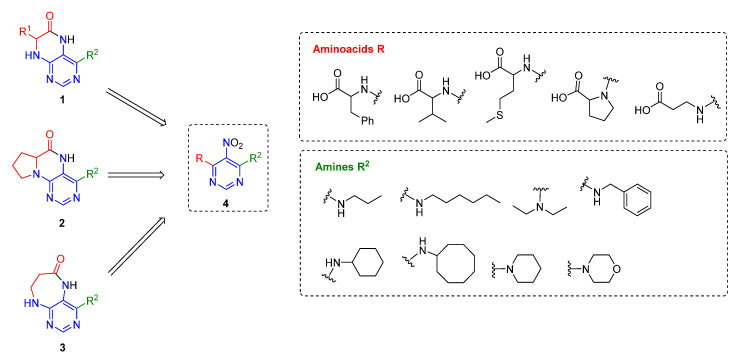
Overview of the target compounds and used building blocks.

The literature described methodologies leading to the synthesis of 7,8-dihydropteridin-6(5*H*)-ones based on various synthetic approaches comprising either traditional solution-phase synthesis or solid-phase synthesis (Figure 2) [1,2,8,19,20,21,22,23]. The most convenient solution-phase synthesis of dihydropteridinone heterocycle consists of the cyclization of appropriately substituted pyrimidine with modified amino ester. The successful solid-phase synthesis of dihydropteridinones lies in preparing a suitable resin-bound intermediate that is cyclized and subsequently cleaved from the resin. In 2000, Baxter et al. published the first solid-phase synthesis of dihydropteridinones using Wang resin (Figure 3) [19]. This work was followed by Metzger et al., who used ArgoGel resin instead [21].

In contrast to dihydropteridinones, the number of synthetic strategies leading to fused pyrrolopteridinone or pyrimidodiazepinone is rather limited.

The abovementioned examples of biologically active compounds (Figure 1) demonstrate the extensive application of dihydropteridinone-based compounds in the field of medicinal chemistry. Dihydropteridinone offers a diverse range of modifications that may significantly contribute to comprehensive SAR studies. Given this fact, it is highly desirable to find an advantageous synthetic methodology to implement structural diversity from the readily available building blocks through minimum synthetic operations. For this reason, we selected the fast and straightforward solid-phase synthesis strategy leading to the generation of pteridinone and pyrimidodiazepinone libraries for future SAR studies utilizing one versatile building block (Figure 3).

## 2. Results and Discussion

Utilizing the versatile building block 4,6-dichloro-5-nitropyrimidine, we synthesized structurally three different types of compounds **1**–**3** from pyrimidine precursor **4** (Figure 3). To determine the scope and limitations of our synthetic pathway leading to cyclized products, we tested a combination of different amino acids R and primary and secondary amines of varying sizes. The synthesis was enabled by immobilization of amino acids via esters on a Wang resin in all cases.

### 2.1. Synthesis of Dihydropteridinones

**Scheme 1:**
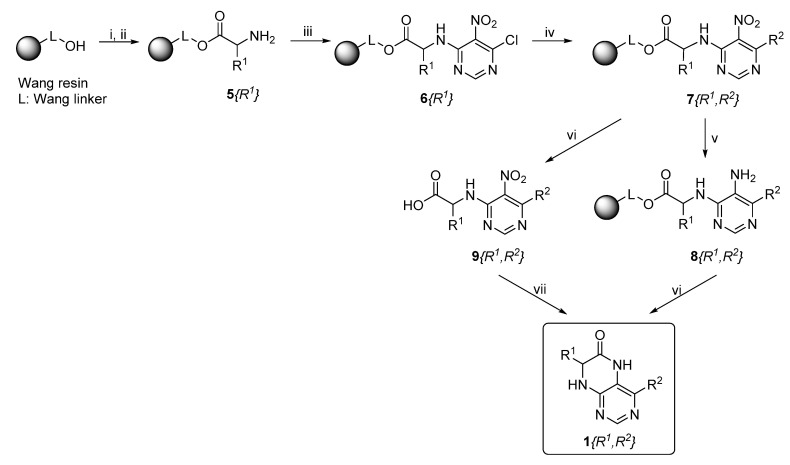
Reagents and conditions: (i) Fmoc-amino acid, *N*-hydroxybenzotriazole (HOBt), DMAP, DIC, DMF/DCM (1:1, *v/v*), rt, 16 h; (ii) 50% piperidine/DMF, rt, 15 min; (iii) 4,6-dichloro-5-nitropyrimidine, DIEA, dry DMF, rt, 2 h; (iv) amine, DIEA, DMF, rt, 16 h; (v) Na_2_S_2_O_4_, K_2_CO_3_, ethylviologen diiodide, DCM/H_2_O (1:1, *v/v*), rt, 16 h; (vi) 50% TFA/DCM, rt, 1 h; and (vii) Zn, AcOH, rt, 1 h.

Synthetic strategy leading to target dihydropteridinones **1** is depicted in Scheme 1. The solid-phase synthesis of intermediate **7** was performed according to our previous reports [24,25,26]. Briefly, the Wang resin was acylated with Fmoc protected Phe, Val, or Met. This reaction was followed by cleavage of the Fmoc-protecting group and nucleophilic reaction with 4,6-dichloro-5-nitropyrimidine giving intermediates **6**. As we described earlier, these intermediates are not stable and were immediately reacted with an appropriate amine, affording resin-bound nitro derivatives **7**.

Further, the nitro group of **7** was reduced using sodium dithionite under phase-transfer catalysis conditions in a DCM–water solution. Finally, TFA-mediated cleavage from the polymer supports triggered cyclization of the dihydropteridinone heterocycles **1**. All products **1** were obtained in excellent crude purities (estimated from UV−vis spectra at 210–400 nm). However, overall yields were meagre, especially for methionine amino acid (below 20%). Subsequently, we found out that cyclization leading to dihydropteridinones **1** occurred spontaneously during the nitro group reduction and inadvertently cleaved desired products were wash out with washing solvents.

For this reason, we changed the strategy and cleaved intermediate **7** from the resin before the nitro group reduction. After simple evaporation of the cleavage cocktail, crude intermediates **9** were subjected to reducing nitro groups using Zn in acetic acid simultaneously, followed by immediate acid-catalyzed cyclization. Products **1** were obtained in excellent crude purities in most cases and overall yields 41–92% after column chromatography (Table 1).

### 2.2. Synthesis of Tetrahydropyrrolopteridinones

**Scheme 2:**
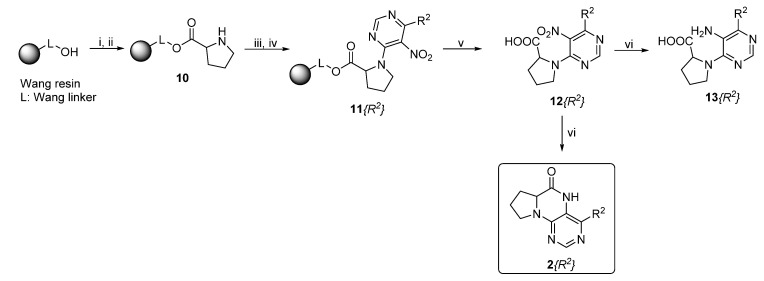
Reagents and conditions: (i) Fmoc-Pro-OH, *N*-hydroxybenzotriazole (HOBt), DMAP, DIC, DMF/DCM (1:1, *v/v*), rt, 16 h; (ii) 50% piperidine/DMF, rt, 15 min; (iii) 4,6-dichloro-5-nitropyrimidine, DIEA, dry DMF, rt, 2 h; (iv) amine, DIEA, DMF, rt, 16 h; (v) 50% TFA/DCM, rt, 1 h; and (vi) Zn, AcOH, rt, 3 h.

Similarly, as dihydropteridinones **1**, a series of tetrahydropyrrolopteridinones **2** was prepared, as shown in Scheme 2. Briefly, nitro derivatives **12** were prepared from Wang resin acylated with Fmoc protected proline followed by deprotection, nucleophilic substitution with 4,6-dichloro-5-nitropyrimidine, further nucleophilic substitution with various amines, and subsequent cleavage from the resin. Zinc-mediated reduction of the nitro group in acetic acid for 3 h yielded desired pyrrolopteridinones **2** (Table 2). The willingness to cyclization is a bit lower compared to the higher mentioned dihydropteridinones **1**. When a shorter reaction time was used, amino derivatives **13** were also observed.

### 2.3. Synthesis of Pyrimidodiazepinones

**Scheme 3:**
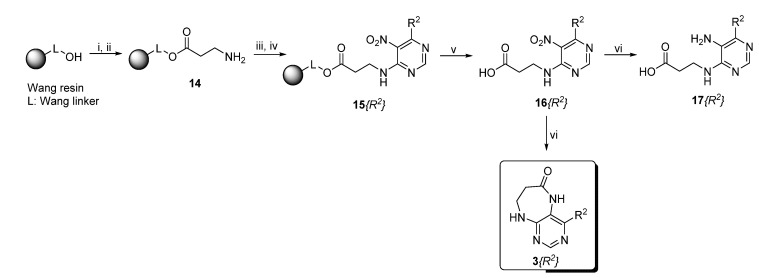
Reagents and conditions: (i) Fmoc-β-Ala-OH, *N*-hydroxybenzotriazole (HOBt), DMAP, DIC, DMF/DCM (1:1, *v/v*), rt, 16 h; (ii) 50% piperidine/DMF, rt, 15 min; (iii) 4,6-dichloro-5-nitropyrimidine, DIEA, dry DMF, rt, 2 h; (iv) amine, DIEA, DMF, rt, 16 h; (v) 50% TFA/DCM, rt, 1 h; and (vi) Zn, AcOH, 80 °C, 3–13 h.

Finally, we tested the scope and limitations of reported cyclization during the preparation of pyrimidodiazepinones **3** (Scheme 3). The required β-alanine intermediates **16** were synthesized in a similar way to previous dihydropteridinones. However, β-alanine substituent emerged unwilling to cyclize giving desired pyrimidodiazepinones **3**. When the reduction with expected simultaneous cyclization was performed at room temperature for 3 h, as in the previous case of pyrrolopteridinones **3**, only noncyclized amino derivatives **17** were observed. For this reason, a prolonged time of 16 h was applied; however, only traces of product **3a** were apparent after this reaction time, as shown in Table 3.

Subsequently, we found out that heating to higher reaction temperature accelerated the conversion. When the reaction was carried out at 50 °C, cyclized products **3** were apparent beside noncyclized amines **17** (according to LC-MS traces at 205−400 nm). Finally, we found out that the cyclization could be achieved at 80 °C using at least 3 h reaction time. It is worth noticing that the willingness to cyclize depends on the type of modification R^2^ (Table 3). Products **3** were obtained in crude purities 49–69% and overall yields 38–55% after column chromatography (Table 4).

## 3. Materials and Methods

Solvents and chemicals were purchased from Sigma-Aldrich (St. Louis, MO, USA) or Fluorochem (Hadfield, UK). The polystyrene resins were purchased from Aapptec (Brossard, Canada). The synthesis was performed on Domino Blocks in disposable polypropylene reaction vessels obtained from Torviq (Niles, MI, USA). Analytical thin-layer chromatography (TLC) was performed using aluminum plates precoated with silica gel (silica gel 60 F254).

All reactions were carried out at room temperature (21 °C) unless stated otherwise. Resin slurry was washed with the appropriate solvent (10 mL per 1 g) by shaking for 1 min. All intermediates were characterized by the LC-MS analysis. For this purpose, a sample of the polymer-bound compound (~5 mg) was treated with 50% trifluoroacetic acid (TFA) in dichloromethane (DCM) for 30 min. Residual solvents were evaporated by a stream of nitrogen and residuum extracted into 1 mL of MeOH.

The LC-MS analyses were carried out on the UHPLC-MS system (Waters, Santa Clara, USA). This system consists of UHPLC chromatograph Acquity with photodiode array detector and single quadrupole mass spectrometer and uses a XSelect C18 column (2.1 × 50 mm) at 30 °C and flow rate of 600 μL/min. The mobile phase was (A) 10 mM ammonium acetate in HPLC grade water and (B) HPLC grade acetonitrile. A gradient was formed from 10% A to 80% of B in 2.5 min; kept for 1.5 min. The column was re-equilibrated with a 10% solution of B for 1 min. The ESI source operated at a discharge current of 5 μA, vaporizer temperature of 350 °C and capillary temperature of 200 °C.

NMR ^1^H/^13^C spectra were recorded on JEOL ECX-500SS (500 MHz, JEOL Ltd., Tokyo, Japan) or JEOL ECA400II (400 MHz, JEOL Ltd., Tokyo, Japan) spectrometer at magnetic field strengths of 11.75 T (with operating frequencies 500.16 MHz for 1H and 125.77 MHz for 13C) and 9.39 T (with operating frequencies 399.78 MHz for ^1^H and 100.53 MHz for ^13^C) at ambient temperature (∼21 °C). Chemical shifts (δ) are reported in parts per million (ppm), and coupling constants (*J*) are reported in Hertz (Hz). NMR spectra are recorded at room temperature (21°C) and referenced to the residual signals of DMSO-*d_6._* All recorded ^1^H- and ^13^C-NMR spectra are available as Supplementary Materials online

HRMS analysis was performed on LC chromatograph (Dionex UltiMate 3000, Thermo Fischer Scientific, MA, USA) with mass spectrometer Exactive Plus Orbitrap high-resolution (Thermo Fischer Scientific, MA, USA) operating in positive scan mode in the range of 1000–1500 *m*/*z*. Electrospray was used as a source of ionization. Samples were diluted to a final concentration of 0.1 mg/mL in a solution of water and acetonitrile (50:50, *v*/*v*). The samples were injected into the mass spectrometer following HPLC separation on a Phenomenex Gemini column (C18, 50 × 2 mm, 3 µm particle) using an isocratic mobile phase of 0.01 M MeCN/ammonium acetate (80/20) at a flow rate of 0.3 mL/min.

### 3.1. Chemistry

#### 3.1.1. Acylation with Amino Acids

The Wang resin (loading 1.0 mmol/g, ~1 g) was washed three times with DCM. A solution consisting of amino acid (2 mmol), HOBt (2 mmol), DMAP (0.5 mmol), and DIC (2 mmol) in DMF/DCM (1:1, *v/v*, 10 mL) was added to the resin. The resin slurry was shaken at rt for 16 h. The resin was washed three times with DMF and three times with DCM. Next, the Fmoc protecting group was removed by exposure to 50% piperidine in DMF (*v/v* 10 mL) for 15 min, and then the resin was washed three times with DMF and three times with DCM.

#### 3.1.2. Reaction with 4,6-dichloro-5-nitropyrimidines and Amines (Resins **7**, **11,** and **15**)

Resins **5**, **10,** and **14** (~1 g) was washed three times with dry DMF and reacted with a solution consisting of 4,6-dichloro-5-nitropyrimidine (5 mmol) and DIEA (5 mmol) in dry DMF (10 mL) at rt for 16 h. The resin was washed five times with DMF and three times with DCM and reacted with a solution consisting of amine (1.25 mmol) and DIEA (1.25 mmol) in DMF (2.5 mL) at rt for 2 h. The resin was then washed three times with DMF and three times with DCM.

#### 3.1.3. Reduction of the Nitro Group on Solid-phase Support (Resins **8**)

Resins **7** (~250 mg) was washed three times with DCM. A solution of Na_2_S_2_O_4_ (2.5 mmol), K_2_CO_3_ (3.0 mmol), and ethyl viologen diiodide (0.25 mmol) in water (2.5 mL) and DCM (2.5 mL) was added to the resin. The resin slurry was shaken at rt for 16 h. The resin was then washed three times with each solvent: DCM/water (1:1, *v*/*v*), DMF, and DCM.

#### 3.1.4. Cleavage from Resin with TFA (Compounds **9**, **12**, and **16**)

Resins **7**, **11,** and **15** (~250 mg) were each treated with 2 mL of a solution consisting of TFA/DCM (1:1, *v*/*v*) for 1 h. The cleavage cocktail was collected, and the resin was washed three times with 50% TFA in DCM. The combined extracts were evaporated by a stream of nitrogen.

#### 3.1.5. Reduction with Simultaneous Cyclization and Isolation (Compounds **1**–**3**)

The oily nitro derivatives **9**, **12,** and **16** were dissolved in acetic acid (3 mL), and powdered zinc (0.5 g) was added. The reaction mixture was stirred at room temperature for 1 h to obtain dihydropteridinones **1**, at room temperature for 3 h to get tetrahydropyrrolopteridinones **2**, and at 80 °C for 3–13 h to obtain pyrimidodiazepinones **3**. The solution was filtered, evaporated to dryness, and purified by column chromatography.

### 3.2. Analytical Data of Individual Compounds

7-Isopropyl-4-(propylamino)-7,8-dihydropteridin-6(5*H*)-one (**1a**).Pale-yellow solid. Yield: 87% (27.3 mg). ^1^H-NMR (400 MHz, CDCl_3_) δ 10.86 (s, 1H), 7.96 (s, 1H), 5.88 (br. s, 1H), 5.64 (br. s, 1H), 4.07 (d, *J* = 3.2 Hz, 1H), 3.51–3.38 (m, 2H), 2.39–2.24 (m, 1H), 1.73–1.59 (m, 2H), 1.08 (d, *J* = 7.0 Hz, 3H), 1.04–0.97 (m, 6H). ^13^C-NMR (101 MHz, CDCl_3_) δ 168.07, 151.86, 149.49, 148.11, 98.10, 61.32, 43.50, 32.56, 22.88, 18.82, 17.12, 11.70. HRMS: *m*/*z*: calcd for C_12_H_20_N_5_O^+^: 250.1662 [M + H]^+^; found: 250.1663.


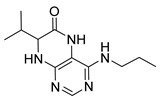


4-(Hexylamino)-7-isopropyl-7,8-dihydropteridin-6(5*H*)-one (**1b**). Pale-yellow solid. Yield: 67% (21.8 mg). ^1^H-NMR (400 MHz, DMSO-*d*_6_) δ 9.71 (s, 1H), 7.74 (s, 1H), 7.01 (br. s, 1H), 6.25 (t, *J* = 4.8 Hz, 1H), 3.79–3.75 (m, 1H), 2.12–1.97 (m, 1H), 1.60–1.45 (m, 2H), 1.41–1.21 (m, 8H), 0.98–0.78 (m, 9H). ^13^C-NMR (101 MHz, DMSO-*d*_6_) δ 165.65, 152.05, 149.29, 148.52, 98.67, 60.90, 41.00, 32.97, 31.59, 29.62, 26.67, 22.61, 18.81, 18.02, 14.45). HRMS: *m*/*z*: calcd for C_15_H_26_N_5_O^+^: 292.2132 [M + H]^+^; found: 292.2130.


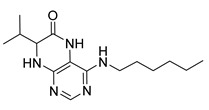


4-(Diethylamino)-7-isopropyl-7,8-dihydropteridin-6(5*H*)-one (**1c**). Pale-yellow solid. Yield: 74% (22.1 mg). ^1^H-NMR (400 MHz, DMSO-*d*_6_) δ 9.45 (s, 1H), 7.83 (s, 1H), 7.51 (d, *J* = 2.5 Hz, 1H), 3.59 (dd, *J* = 5.7, 2.9 Hz, 1H), 3.50–3.38 (m, 2H), 3.15 (dq, *J* = 14.0, 7.0 Hz, 2H), 1.91 (dq, *J* = 13.3, 6.6 Hz, 1H), 0.99 (t, *J* = 7.0 Hz, 6H), 0.91 (d, *J* = 6.9 Hz, 3H), 0.85 (d, *J* = 6.8 Hz, 3H). ^13^C-NMR (101 MHz, DMSO-*d*_6_) δ 164.77, 151.98, 151.34, 150.91, 103.66, 60.81, 42.74, 31.43, 18.35, 17.83, 12.84. HRMS: *m*/*z*: calcd for C_13_H_22_N_5_O^+^: 264.1819 [M + H]^+^; found: 264.1821.


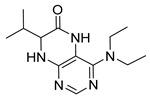


4-(Benzylamino)-7-isopropyl-7,8-dihydropteridin-6(5*H*)-one (**1d**). Pale-yellow solid. Yield: 75% (25.0 mg). ^1^H-NMR (400 MHz, DMSO-*d*_6_) δ 9.74 (s, 1H), 7.75 (s, 1H), 7.42–7.28 (m, 4H), 7.28–7.20 (m, 1H), 7.07 (br. s, 1H), 6.75 (t, *J* = 5.5 Hz, 1H), 4.66–4.47 (m, 2H), 3.79 (dd, *J* = 4.1, 2.3 Hz, 1H), 2.05 (dq, *J* = 11.2, 6.9 Hz, 1H), 0.92 (d, *J* = 7.0 Hz, 3H), 0.85 (d, *J* = 6.8 Hz, 3H). ^13^C-NMR (101 MHz, DMSO-*d*_6_) δ 165.07, 151.54, 149.08, 147.70, 139.89, 128.24, 127.43, 126.73, 98.40, 60.36, 43.82, 32.45, 18.27, 17.47. HRMS: *m*/*z*: calcd for C_16_H_20_N_5_O^+^: 298.1662 [M + H]^+^; found: 298.1662.


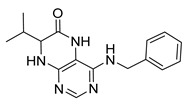


4-(Cyclohexylamino)-7-isopropyl-7,8-dihydropteridin-6(5*H*)-one (**1e**). Pale-yellow solid. Yield: 66% (21.5 mg). ^1^H-NMR (400 MHz, DMSO-*d*_6_) δ 9.75 (s, 1H), 7.72 (s, 1H), 6.95 (d, *J* = 2.1 Hz, 1H), 6.08 (d, *J* = 7.2 Hz, 1H), 3.88–3.77 (m, 1H), 3.75 (dd, *J* = 4.3, 2.3 Hz, 1H), 2.03 (dtd, *J* = 13.8, 6.9, 4.5 Hz, 1H), 1.93–1.84 (m, 2H), 1.77–1.64 (m, 2H), 1.62–1.53 (m, 1H), 1.37–1.08 (m, 5H), 0.91 (d, *J* = 7.0 Hz, 3H), 0.84 (d, *J* = 6.8 Hz, 3H). ^13^C-NMR (101 MHz, DMSO-*d*_6_) δ 165.13, 151.65, 148.94, 147.32, 97.95, 60.34, 48.79, 32.87, 32.32, 25.38, 24.68, 18.29, 17.48. HRMS: *m*/*z*: calcd for C_15_H_24_N_5_O^+^: 290.1975 [M + H]^+^; found: 290.1973.


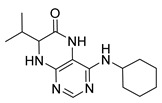


4-(Cyclooctylamino)-7-isopropyl-7,8-dihydropteridin-6(5*H*)-one (**1f**). Pale-yellow solid. Yield: 91% (32.2 mg). ^1^H-NMR (400 MHz, DMSO-*d*_6_) δ 9.79 (s, 1H), 7.72 (s, 1H), 6.93 (d, *J* = 2.0 Hz, 1H), 6.08 (d, *J* = 7.5 Hz, 1H), 4.16–4.06 (m, 1H), 3.75 (dd, *J* = 4.2, 2.3 Hz, 1H), 2.10–1.97 (m, 1H), 1.85–1.72 (m, 2H), 1.72–1.62 (m, 2H), 1.62–1.41 (m, 10H), 0.91 (d, *J* = 7.0 Hz, 3H), 0.83 (d, *J* = 6.8 Hz, 3H). ^13^C-NMR (101 MHz, DMSO-*d*_6_) δ 165.18, 151.67, 148.89, 147.15, 98.06, 60.32, 49.62, 32.33, 31.37, 27.07, 25.05, 23.22, 18.29, 17.48. HRMS: *m*/*z*: calcd for C_17_H_28_N_5_O^+^: 318.2288 [M + H]^+^; found: 318.2288.


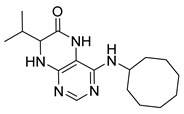


7-Isopropyl-4-(piperidin-1-yl)-7,8-dihydropteridin-6(5*H*)-one (**1g**). Pale-yellow solid. Yield: 90% (28.0 mg). ^1^H-NMR (400 MHz, DMSO-*d*_6_) δ 9.53 (s, 1H), 7.85 (s, 1H), 7.54 (d, *J* = 2.8 Hz, 1H), 3.63 (dd, *J* = 5.3, 3.0 Hz, 1H), 3.30–3.21 (m, 2H), 3.12–3.01 (m, 2H), 1.96 (dq, *J* = 13.5, 6.8 Hz, 1H), 1.77–1.65 (m, 2H), 1.58–1.41 (m, 4H), 0.91 (d, *J* = 6.9 Hz, 3H), 0.84 (d, *J* = 6.8 Hz, 3H). ^13^C-NMR (101 MHz, DMSO-*d*_6_) δ 164.69, 152.07, 151.88, 151.42, 103.70, 60.68, 48.03, 31.84, 24.91, 24.12, 18.39, 17.73. HRMS: *m*/*z*: calcd for C_14_H_22_N_5_O^+^: 276.1819 [M + H]^+^; found: 276.1821.


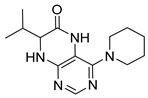


7-Isopropyl-4-morpholino-7,8-dihydropteridin-6(5*H*)-one (**1h**). Pale-yellow solid. Yield: 92% (28.9 mg). ^1^H-NMR (400 MHz, DMSO-*d*_6_) δ 9.79 (s, 1H), 7.88 (s, 1H), 7.64 (d, *J* = 2.5 Hz, 1H), 3.85–3.76 (m, 2H), 3.69–3.57 (m, 3H), 3.30–3.22 (m, 2H, overlapped with water), 3.06–2.97 (m, 2H), 2.03–1.92 (m, 1H), 0.91 (d, *J* = 6.9 Hz, 3H), 0.84 (d, *J* = 6.8 Hz, 3H). ^13^C-NMR (101 MHz, DMSO-*d*_6_) δ 164.81, 151.93, 151.39, 151.34, 104.34, 65.65, 60.63, 47.53, 31.97, 18.39, 17.72. HRMS: *m*/*z*: calcd for C_13_H_20_N_5_O_2_^+^: 278.1612 [M + H]^+^; found: 278.1609.


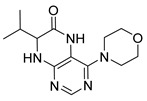


7-Benzyl-4-(propylamino)-7,8-dihydropteridin-6(5*H*)-one (**1i**). Yellow solid. Yield: 89% (29.8 mg). ^1^H-NMR (400 MHz, DMSO-*d*_6_) δ 9.61 (s, 1H), 7.68 (s, 1H), 7.24–7.09 (m, 5H), 6.83 (d, *J* = 1.4 Hz, 1H), 6.14 (t, *J* = 5.2 Hz, 1H), 4.28 (td, *J* = 5.1, 1.8 Hz, 1H), 3.26–3.16 (m, 2H), 3.03 (dd, *J* = 13.6, 5.1 Hz, 1H), 2.92 (dd, *J* = 13.6, 5.2 Hz, 1H), 1.54–1.40 (m, 2H), 0.87 (t, *J* = 7.4 Hz, 3H). ^13^C-NMR (101 MHz, DMSO-*d*_6_) δ 165.25, 151.54, 148.48, 148.02, 136.60, 129.86, 127.79, 126.31, 98.41, 56.17, 42.16, 38.38, 22.31, 11.42. HRMS: *m*/*z*: calcd for C_16_H_20_N_5_O^+^: 298.1662 [M + H]^+^; found: 298.1662.


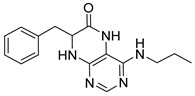


7-Benzyl-4-(hexylamino)-7,8-dihydropteridin-6(5*H*)-one (**1j**). Yellow solid. Yield: 76% (19.3 mg). ^1^H-NMR (400 MHz, DMSO-*d*_6_) δ 9.60 (s, 1H), 7.68 (s, 1H), 7.22–7.12 (m, 5H), 6.83 (d, *J* = 1.5 Hz, 1H), 6.10 (t, *J* = 5.2 Hz, 1H), 4.28 (td, *J* = 5.1, 1.8 Hz, 1H), 3.24 (qd, *J* = 6.7, 1.2 Hz, 2H), 3.02 (dd, *J* = 13.6, 5.1 Hz, 1H), 2.91 (dd, *J* = 13.6, 5.2 Hz, 1H), 1.49–1.40 (m, 2H), 1.32–1.22 (m, 6H), 0.89–0.84 (m, 3H). ^13^C-NMR (101 MHz, DMSO-*d*_6_) δ 165.25, 151.55, 148.46, 148.00, 136.60, 129.86, 127.78, 126.29, 98.40, 56.17, 40.30, 38.38, 31.05, 29.04, 26.09, 22.08, 13.91. HRMS: *m*/*z*: calcd for C_19_H_26_N_5_O^+^: 340.2132 [M + H]^+^; found: 340.2131.


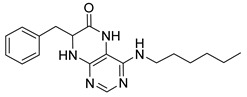


7-Benzyl-4-(diethylamino)-7,8-dihydropteridin-6(5*H*)-one (**1k**). Yellow solid. Yield: 91% (31.6 mg). ^1^H-NMR (400 MHz, DMSO-*d*_6_) δ 9.29 (s, 1H), 7.79 (s, 1H), 7.37 (d, *J* = 2.2 Hz, 1H), 7.20–7.11 (m, 5H), 4.22 (td, *J* = 5.3, 2.3 Hz, 1H), 3.18 (dq, *J* = 14.1, 7.0 Hz, 2H), 3.04 (dq, *J* = 14.1, 7.1 Hz, 2H), 2.93 (qd, *J* = 13.6, 5.5 Hz, 2H), 0.91 (t, *J* = 7.1 Hz, 6H). ^13^C-NMR (101 MHz, DMSO-*d*_6_) δ 164.66, 151.61, 151.17, 150.97, 136.26, 129.74, 127.90, 126.45, 104.21, 56.35, 42.55, 38.31, 12.68. HRMS: *m*/*z*: calcd for C_17_H_22_N_5_O^+^: 312.1819 [M + H]^+^; found: 312.1818.


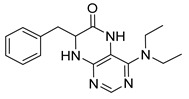


7-Benzyl-4-(benzylamino)-7,8-dihydropteridin-6(5*H*)-one (**1l**). Yellow solid. Yield: 80% (30.1 mg). ^1^H-NMR (400 MHz, DMSO-*d*_6_) δ 9.63 (s, 1H), 7.68 (s, 1H), 7.35–7.29 (m, 2H), 7.26–7.21 (m, 3H), 7.20–7.12 (m, 5H), 6.95 (br s, 1H), 6.63 (t, *J* = 5.6 Hz, 1H), 4.50 (qd, *J* = 15.2, 5.5 Hz, 2H), 4.31 (td, *J* = 4.9, 1.6 Hz, 1H), 3.04 (dd, *J* = 13.6, 4.9 Hz, 1H), 2.93 (dd, *J* = 13.6, 5.1 Hz, 1H). ^13^C-NMR (101 MHz, DMSO-*d*_6_) δ 165.22, 151.48, 148.78, 147.62, 139.91, 136.50, 129.90, 128.19, 127.78, 127.25, 126.67, 126.33, 98.53, 56.18, 43.58, 38.56. HRMS: *m*/*z*: calcd for C_20_H_20_N_5_O^+^: 346.1662 [M + H]^+^; found: 346.1662.


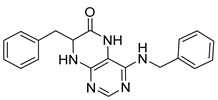


7-Benzyl-4-(cyclohexylamino)-7,8-dihydropteridin-6(5*H*)-one (**1m**). Yellow solid. Yield: 82% (30.2 mg). ^1^H-NMR (400 MHz, DMSO-*d*_6_) δ 9.66 (s, 1H), 7.67 (s, 1H), 7.22–7.11 (m, 5H), 6.81 (br s, 1H), 5.98 (d, *J* = 7.3 Hz, 1H), 4.28 (td, *J* = 5.0, 1.6 Hz, 1H), 3.85–3.70 (m, 1H), 3.03 (dd, *J* = 13.6, 5.0 Hz, 1H), 2.91 (dd, *J* = 13.6, 5.2 Hz, 1H), 1.83 (br s, 2H), 1.73–1.65 (m, 2H), 1.61–1.52 (m, 1H), 1.35–1.21 (m, 2H), 1.21–1.05 (m, 3H). ^13^C-NMR (101 MHz, DMSO-*d*_6_) δ 165.30, 151.53, 148.60, 147.28, 136.62, 129.86, 127.77, 126.32, 98.24, 56.12, 48.65, 38.32, 32.79, 25.35, 24.60. HRMS: *m*/*z*: calcd for C_19_H_24_N_5_O^+^: 338.1975 [M + H]^+^; found: 338.1974.


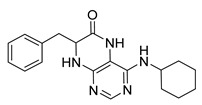


7-Benzyl-4-(cyclooctylamino)-7,8-dihydropteridin-6(5*H*)-one (**1n**). Yellow solid. Yield: 92% (36.3 mg). ^1^H-NMR (400 MHz, DMSO-*d*_6_) δ 9.70 (s, 1H), 7.67 (s, 1H), 7.24–7.08 (m, 5H), 6.80 (s, 1H), 5.97 (d, *J* = 7.4 Hz, 1H), 4.28 (br s, 1H), 4.05 (br s, 1H), 3.03 (dd, *J* = 13.5, 4.9 Hz, 1H), 2.92 (dd, *J* = 13.5, 5.0 Hz, 1H), 1.76–1.39 (m, 14H). ^13^C-NMR (101 MHz, DMSO-*d*_6_) δ 165.34, 151.53, 148.54, 147.10, 136.60, 129.87, 127.76, 126.30, 98.34, 56.12, 49.51, 38.36, 31.24, 31.16, 27.09, 27.06, 25.02, 23.14. HRMS: *m*/*z*: calcd for C_21_H_28_N_5_O^+^: 366.2288 [M + H]^+^; found: 366.2288.


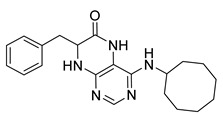


7-Benzyl-4-(piperidin-1-yl)-7,8-dihydropteridin-6(5*H*)-one (**1o**). Yellow solid. Yield: 89% (36.3 mg). ^1^H-NMR (400 MHz, DMSO-*d*) δ 9.35 (s, 1H), 7.77 (s, 1H), 7.40 (d, *J* = 1.8 Hz, 1H), 7.17–7.08 (m, 5H), 4.28–4.22 (m, 1H), 3.08–2.80 (m, 6H), 1.67–1.57 (m, 2H), 1.49 (dt, *J* = 10.6, 5.2 Hz, 2H), 1.45–1.36 (m, 2H). ^13^C-NMR (101 MHz, DMSO-*d*_6_) δ 164.60, 151.88, 151.51, 151.23, 136.11, 129.80, 127.85, 126.39, 103.62, 56.36, 48.04, 38.57, 24.87, 24.06. HRMS: *m*/*z*: calcd for C_18_H_22_N_5_O^+^: 324.1819 [M + H]^+^; found: 324.1818.


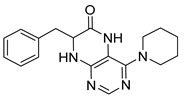


7-Benzyl-4-morpholino-7,8-dihydropteridin-6(5*H*)-one (**1p**). Yellow solid. Yield: 70% (22.8 mg). ^1^H-NMR (400 MHz, DMSO-*d*_6_) δ 9.62 (s, 1H), 7.79 (s, 1H), 7.52 (d, *J* = 1.6 Hz, 1H), 7.16–7.06 (m, 5H), 4.31 –4.24 (m, 1H), 3.76–3.66 (m, 2H), 3.58–3.49 (m, 2H), 3.07–2.96 (m, 3H), 2.90 (dd, *J* = 13.5, 5.0 Hz, 1H), 2.83–2.74 (m, 2H). ^13^C-NMR (101 MHz, DMSO-*d*_6_) δ 164.80, 151.66, 151.18, 151.01, 136.03, 129.86, 127.87, 126.41, 104.09, 65.56, 56.34, 47.54, one carbon overlapped with DMSO (according to HMQC). HRMS: *m*/*z*: calcd for C_17_H_20_N_5_O_2_^+^: 326.1612 [M + H]^+^; found: 326.1611.


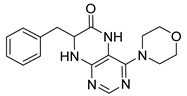


7-(2-(Methylthio)ethyl)-4-(propylamino)-7,8-dihydropteridin-6(5*H*)-one (**1q**). Yellow solid. Yield: 68% (19.6 mg). ^1^H-NMR (500 MHz, DMSO-*d*_6_) δ 9.71 (s, 1H), 7.75 (s, 1H), 7.01 (d, *J* = 1.3 Hz, 1H), 6.29 (t, *J* = 5.2 Hz, 1H), 4.05 (td, *J* = 5.6, 1.7 Hz, 1H), 3.30–3.23 (m, 2H), 2.64–2.49 (m, 2H, overlapped with DMSO), 2.03 (s, 3H), 1.96–1.86 (m, 2H), 1.51 (dt, *J* = 14.5, 7.2 Hz, 2H), 0.90 (t, *J* = 7.4 Hz, 3H). ^13^C-NMR (126 MHz, DMSO-*d*_6_) δ 165.72, 151.71, 148.71, 148.34, 98.70, 53.85, 42.27, 32.00, 28.71, 22.35, 14.47, 11.47. HRMS: *m*/*z*: calcd for C_12_H_20_N_5_OS^+^: 282.1383 [M + H]^+^; found: 282.1382.


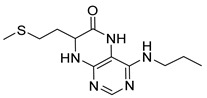


4-(Hexylamino)-7-(2-(methylthio)ethyl)-7,8-dihydropteridin-6(5*H*)-one (**1r**). Yellow solid. Yield: 66% (21.4 mg). ^1^H-NMR (400 MHz, DMSO-*d*_6_) δ 9.71 (s, 1H), 7.75 (s, 1H), 7.00 (d, *J* = 1.3 Hz, 1H), 6.26 (t, *J* = 5.1 Hz, 1H), 4.05 (td, *J* = 5.6, 1.7 Hz, 1H), 3.34–3.27 (m, 2H, overlapped with water), 2.64–2.49 (m, 2H, overlapped with DMSO), 2.03 (s, 3H), 1.97–1.87 (m, 2H), 1.54–1.46 (m, 2H), 1.35–1.25 (m, 6H), 0.87 (t, *J* = 6.9 Hz, 3H). ^13^C-NMR (101 MHz, DMSO-*d*_6_) δ 165.71, 151.71, 148.69, 148.32, 98.68, 53.84, 40.42, 32.00, 31.05, 29.07, 28.70, 26.13, 22.07, 14.46, 13.91. HRMS: *m*/*z*: calcd for C_15_H_26_N_5_OS^+^: 324.1853 [M + H]^+^; found: 324.1852.


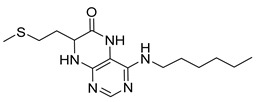


4-(Diethylamino)-7-(2-(methylthio)ethyl)-7,8-dihydropteridin-6(5*H*)-one (**1s**). Yellow solid. Yield: 76% (22.8 mg). ^1^H-NMR (500 MHz, DMSO-*d*_6_) δ 9.47 (s, 1H), 7.86 (s, 1H), 7.45 (d, *J* = 2.0 Hz, 1H), 3.96–3.91 (m, 1H), 3.42–3.34 (m, 2H), 3.33–3.23 (m, 2H), 2.62–2.52 (m, 2H), 2.03 (s, 3H), 1.95–1.87 (m, 1H), 1.86–1.78 (m, 1H), 1.01 (t, *J* = 7.1 Hz, 6H). ^13^C-NMR (101MHz, DMSO-*d*_6_) δ 165.34, 152.15, 151.40, 151.25, 103.67, 53.73, 42.76, 30.79, 28.79, 14.45, 12.85. HRMS: *m*/*z*: calcd for C_13_H_22_N_5_OS^+^: 296.1540 [M + H]^+^; found: 296.1539.


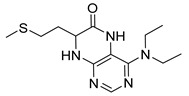


4-(Benzylamino)-7-(2-(methylthio)ethyl)-7,8-dihydropteridin-6(5*H*)-one (**1t**). Yellow solid. Yield: 41% (13.4 mg). ^1^H-NMR (400 MHz, DMSO-*d*_6_) δ 9.71 (s, 1H), 7.73 (s, 1H), 7.32–7.25 (m, 4H), 7.23–7.17 (m, 1H), 7.05 (s, 1H), 6.75 (t, *J* = 5.4 Hz, 1H), 4.59–4.46 (m, 2H), 4.07–4.00 (m, 1H), 2.60–2.49 (m, 2H, overlapped with DMSO), 1.98 (s, 3H), 1.95–1.85 (m, 2H). ^13^C-NMR (101 MHz, DMSO-*d*_6_): δ 165.70, 151.70, 148.99, 148.02, 139.86, 128.27, 127.47, 126.78, 98.94, 53.87, 43.84, 32.07, 28.73, 14.50. HRMS: *m*/*z*: calcd for C_16_H_20_N_5_OS^+^: 330.1383 [M + H]^+^; found: 330.1382.


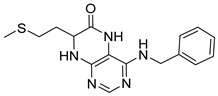


4-(Cyclohexylamino)-7-(2-(methylthio)ethyl)-7,8-dihydropteridin-6(5*H*)-one (**1u**). Yellow solid. Yield: 79% (25.7 mg). ^1^H-NMR (400 MHz, DMSO-*d*_6_) δ 9.77 (s, 1H), 7.74 (s, 1H), 7.00 (d, *J* = 1.4 Hz, 1H), 6.13 (d, *J* = 7.2 Hz, 1H), 4.05 (td, *J* = 5.6, 1.7 Hz, 1H), 3.91–3.78 (m, 1H), 2.64–2.48 (m, 2H, overlapped with DMSO), 2.03 (s, 3H), 1.98–1.83 (m, 4H), 1.75–1.67 (m, *2*H), 1.62–1.54 (m, 1H), 1.36–1.09 (m, 5H). ^13^C-NMR (101 MHz, DMSO-*d*_6_) δ 165.72, 151.71, 148.81, 147.57, 98.48, 53.81, 48.79, 32.85, 31.99, 28.71, 25.36, 24.64, 14.47. HRMS: *m*/*z*: calcd for C_15_H_24_N_5_OS^+^: 322.1696 [M + H]^+^; found: 322.1694.


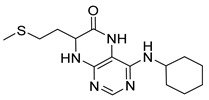


4-(Cyclooctylamino)-7-(2-(methylthio)ethyl)-7,8-dihydropteridin-6(5*H*)-one (**1v**). Yellow solid. Yield: 60% (20.9 mg). ^1^H-NMR (400 MHz, DMSO-*d*_6_) δ 9.81 (s, 1H), 7.75 (s, 1H), 6.98 (d, *J* = 1.4 Hz, 1H), 6.13 (d, *J* = 7.5 Hz, 1H), 4.18–4.08 (m, 1H), 4.05 (td, *J* = 5.6, 1.6 Hz, 1H), 2.65–2.50 (m, 2H), 2.03 (s, 3H), 1.99–1.84 (m, 2H), 1.83–1.73 (m, 2H), 1.72–1.63 (m, 2H), 1.61–1.40 (m, 10H). ^13^C-NMR (101 MHz, DMSO-*d*_6_) δ 166.31, 152.29, 149.30, 147.95, 99.13, 54.36, 50.18, 32.53, 31.85, 29.27, 27.63, 25.59, 23.74, 15.02. HRMS: *m*/*z*: calcd for C_17_H_28_N_5_OS^+^: 350.2009 [M + H]^+^; found: 350.2011.


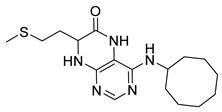


7-(2-(Methylthio)ethyl)-4-(piperidin-1-yl)-7,8-dihydropteridin-6(5*H*)-one (**1w**). Yellow solid. Yield: 63% (19.5 mg). ^1^H-NMR (500 MHz, DMSO-*d*_6_) δ 9.55 (s, 1H), 7.88 (s, 1H), 7.49 (d, *J* = 1.9 Hz, 1H), 3.98–3.93 (m, 1H), 3.26–3.20 (m, 2H), 3.19–3.12 (m, 2H), 2.62–2.52 (m, 2H), 2.03 (s, 3H), 1.97–1.89 (m, 1H), 1.88–1.80 (m, 1H), 1.69–1.60 (m, 2H), 1.59–1.51 (m, 4H). ^13^C-NMR (126 MHz, DMSO-*d*_6_) δ 165.21, 152.41, 151.94, 151.46, 103.96, 53.75, 48.09, 30.77, 28.79, 24.94, 24.10, 14.45. HRMS: *m*/*z*: calcd for C_14_H_22_N_5_OS^+^: 308.1540 [M + H]^+^; found: 308.1538.


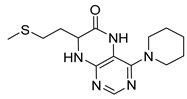


7-(2-(Methylthio)ethyl)-4-morpholino-7,8-dihydropteridin-6(5*H*)-one (**1x**). Yellow solid. Yield: 65% (20.5 mg). ^1^H-NMR (500 MHz, DMSO-*d*_6_) δ 9.81 (s, 1H), 7.91 (s, 1H), 7.59 (d, *J* = 1.9 Hz, 1H), 4.01–3.96 (m, 1H), 3.76–3.72 (m, 2H), 3.71–3.65 (m, 2H), 3.24–3.18 (m, 2H), 3.16–3.10 (m, 2H), 2.62–2.51 (m, 2H), 2.03 (s, 3H), 1.97–1.82 (m, 2H). ^13^C-NMR (126 MHz, DMSO-*d*_6_) δ 165.36, 151.97, 151.66, 151.41, 104.57, 65.67, 53.72, 47.58, 30.96, 28.77, 14.46. HRMS: *m*/*z*: calcd for C_13_H_20_N_5_OS^+^: 310.1332 [M + H]^+^; found: 310.1331.


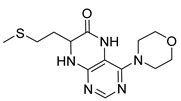


4-(Propylamino)-6a,7,8,9-tetrahydropyrrolo [2,1-h]pteridin-6(5*H*)-one (**2a**). Dark-yellow solid. Yield: 75% (21.3 mg). ^1^H-NMR (500 MHz, DMSO-*d*_6_) δ 9.70 (s, 1H), 7.84 (s, 1H), 6.26 (t, *J* = 5.2 Hz, 1H), 3.99–3.92 (m, 1H), 3.63–3.54 (m, 1H), 3.43–3.36 (m, 1H), 3.33–3.25 (m, 2H, overlapped with water), 2.23–2.11 (m, 1H), 1.99–1.83 (m, 3H), 1.52 (sx, *J* = 7.3 Hz, 2H), 0.90 (t, *J* = 7.4 Hz, 3H). ^13^C-NMR (126 MHz, DMSO-*d*_6_) 165.24, 151.59, 148.16, 148.11, 100.69, 58.62, 45.09, 42.30, 27.68, 22.39, 21.80, 11.44. HRMS: *m*/*z*: calcd for C_12_H_18_N_5_O^+^: 248.1506 [M + H]^+^; found: 248.1507.


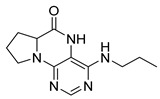


4-(Hexylamino)-6a,7,8,9-tetrahydropyrrolo[2,1-h]pteridin-6(5*H*)-one ***(2*b**). Dark-yellow solid. Yield: 47% (15.3 mg). ^1^H-NMR (400 MHz, DMSO-*d*_6_) δ 9.66 (s, 1H), 7.80 (s, 1H), 6.19 (t, *J* = 5.1 Hz, 1H), 3.95–3.88 (m, 1H), 3.58–3.49 (m, 1H), 3.39–3.32 (m, 1H), 3.31–3.24 (m, 2H), 2.18–2.05 (m, 1H), 1.94–1.79 (m, 3H), 1.52–1.41 (m, 2H), 1.34–1.16 (m, 6H), 0.83 (t, *J* = 6.9 Hz, 3H). ^13^C-NMR (126 MHz, DMSO-*d*_6_) δ 165.24, 151.59, 148.14, 148.09, 100.68, 58.62, 45.08, 40.44, 31.05, 29.12, 27.68, 26.12, 22.07, 21.80, 13.90. HRMS: *m*/*z*: calcd for C_15_H_24_N_5_O^+^: 290.1975 [M + H]^+^; found: 290.1974.


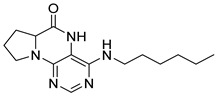


4-(Diethylamino)-6a,7,8,9-tetrahydropyrrolo[2,1-h]pteridin-6(5*H*)-one (**2c**). Dark-yellow solid. Yield: 87% (25.8 mg). ^1^H-NMR (400 MHz, DMSO-*d*_6_) δ 9.46 (s, 1H), 7.92 (s, 1H), 3.95 (dd, *J* = 8.9, 6.9 Hz, 1H), 3.60–3.44 (m, 4H), 3.24–313 (m, 2H), 2.23–2.12 (m, 1H), 2.09–1.91 (m, 3H), 1.03 (t, *J* = 7.1 Hz, 6H). ^13^C-NMR (126 MHz, DMSO-*d*_6_) δ 165.34, 151.78, 151.66, 151.36, 105.65, 58.80, 45.74, 43.21, 27.54, 22.99, 13.37. HRMS: *m*/*z*: calcd for C_13_H_20_N_5_O^+^: 262.1662 [M + H]^+^; found: 262.1663.


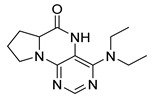


4-(Benzylamino)-6a,7,8,9-tetrahydropyrrolo[2,1-h]pteridin-6(5*H*)-one (**2d**). Dark-yellow solid. Yield: 81% (26.9 mg). ^1^H-NMR (500 MHz, DMSO-*d*_6_) δ 9.75 (s, 1H), 7.86 (s, 1H), 7.35–7.29 (m, 4H), 7.26–7.21 (m, 1H), 6.78 (t, *J* = 5.6 Hz, 1H), 4.58 (d, *J* = 5.6 Hz, 2H), 4.02–3.95 (m, 1H), 3.63–3.55 (m, 1H), 3.45–3.37 (m, 1H), 2.22–2.13 (m, 1H), 1.98–1.84 (m, 3H). ^13^C-NMR (126 MHz, DMSO-*d*_6_) δ 165.18, 151.54, 148.34, 147.74, 139.94, 128.22, 127.40, 126.71, 100.92, 58.63, 45.14, 43.84, 27.73, 21.81. HRMS: *m*/*z*: calcd for C_16_H_18_N_5_O^+^: 296.1506 [M + H]^+^; found: 296.1505.


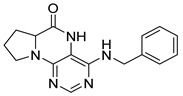


4-(Cyclohexylamino)-6a,7,8,9-tetrahydropyrrolo[2,1-h]pteridin-6(5*H*)-one (**2e**). Dark-yellow solid. Yield: 80% (25.8 mg). ^1^H-NMR (400 MHz, DMSO-*d*_6_) δ 9.76 (s, 1H), 7.83 (s, 1H), 6.10 (d, *J* = 7.3 Hz, 1H), 3.98–3.91 (m, 1H), 3.91–3.80 (m, 1H), 3.62–3.53 (m, 1H), 3.43–3.34 (m, 1H), 2.21–2.10 (m, 1H), 1.98–1.82 (m, 5H), 1.77–1.66 (m, 2H), 1.62–1.53 (m, 1H), 1.38–1.10 (m, 5H). ^13^C-NMR (101 MHz, DMSO-*d*_6_) δ 165.25, 151.59, 148.26, 147.36, 100.51, 58.60, 48.84, 45.10, 32.94, 32.76, 27.70, 25.37, 24.64, 21.80. HRMS: *m*/*z*: calcd for C_15_H_22_N_5_O^+^: 288.1819 [M + H]^+^; found: 288.1818.


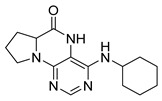


4-(Cyclooctylamino)-6a,7,8,9-tetrahydropyrrolo[2,1-h]pteridin-6(5*H*)-one **(2f).** Dark-yellow solid. Yield: 34% (12.0 mg). ^1^H-NMR (400 MHz, DMSO-*d*_6_) δ 9.81 (s, 1H), 7.84 (s, 1H), 6.10 (d, *J* = 7.5 Hz, 1H), 4.20–4.09 (m, 1H), 4.00–3.91 (m, 1H), 3.61–3.53 (m, 1H), 3.43–3.34 (m, 1H), 2.21–2.10 (m, 1H), 1.98–1.85 (m, 3H), 1.85–1.73 (m, 2H), 1.71–1.62 (m, 2H), 1.62–1.42 (m, 10H). ^13^C-NMR (126 MHz, DMSO-*d*_6_) δ 165.29, 151.61, 148.19, 147.18, 100.59, 58.60, 49.68, 45.12, 31.44, 31.21, 27.70, 27.07, 25.05, 23.20, 23.16, 21.80. HRMS: *m*/*z*: calcd for C_17_H_26_N_5_O^+^: 316.2132 [M + H]^+^; found: 316.2130.


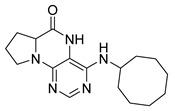


4-(Piperidin-1-yl)-6a,7,8,9-tetrahydropyrrolo[2,1-h]pteridin-6(5*H*)-one (**2g**). Dark-yellow solid. Yield: 60% (18.6 mg). ^1^H-NMR (400 MHz, DMSO-*d*_6_) δ 9.54 (s, 1H), 7.93 (s, 1H), 3.97 (dd, *J* = 8.9, 6.9 Hz, 1H), 3.61–3.45 (m, 2H), 3.43–3.34 (m, 2H), 3.15–3.04 (m, 2H), 2.23–2.12 (m, 1H), 2.09–1.91 (m, 3H), 1.80–1.69 (m, 2H), 1.55 (dt, *J* = 11.2, 5.6 Hz, 2H), 1.50–1.40 (m, 2H). ^13^C-NMR (126 MHz, DMSO-*d*_6_) δ 164.66, 151.86, 151.31, 150.83, 105.35, 58.37, 48.08, 45.19, 27.10, 24.99, 24.14, 22.35. HRMS: *m*/*z*: calcd for C_14_H_20_N_5_O^+^: 274.1662 [M + H]^+^; found: 274.1661.


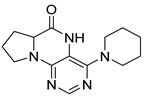


4-Morpholino-6a,7,8,9-tetrahydropyrrolo[2,1-h]pteridin-6(5*H*)-one (**2h**). Dark-yellow solid. Yield: 57% (17.9 mg). ^1^H-NMR (400 MHz, DMSO-*d*_6_) δ 9.75 (s, 1H), 7.92 (s, 1H), 3.95 (dd, *J* = 8.9, 6.9 Hz, 1H), 3.82–3.74 (m, 2H), 3.60–3.43 (m, 4H), 3.39–3.31 (m, 2H), 3.04–2.97 (m, 2H), 2.20–2.09 (m, 1H), 2.06–1.83 (m, 3H). ^13^C-NMR (126 MHz, DMSO-*d*_6_) δ 164.83, 151.26, 151.12, 150.82, 105.96, 65.70, 58.35, 47.56, 45.25, 27.17, 22.29. HRMS: *m*/*z*: calcd for C_13_H_18_N_5_O_2_^+^: 276.1455 [M + H]^+^; found: 276.1456.


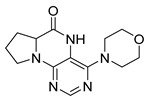


4-(Propylamino)-5,7,8,9-tetrahydro-6*H*-pyrimido[4,5-b][1,4]diazepin-6-one (**3a**). White solid. Yield: 45% (11.7 mg). ^1^H-NMR (500 MHz, DMSO-*d*_6_) δ 8.41 (s, 1H), 7.75 (s, 1H), 6.67 (t, *J* = 4.2 Hz, 1H), 6.36 (t, *J* = 5.3 Hz, 1H), 3.51 (td, *J* = 4.6, 2.2 Hz, 2H), 3.28–3.22 (m, 2H), 2.49–2.47 (m, 2H), 1.58–1.47 (m, 2H), 0.89 (t, *J* = 7.4 Hz, 3H). ^13^C-NMR (126 MHz, DMSO-*d*_6_) δ 172.69, 155.18, 153.13, 152.94, 96.95, 43.17, 42.66, 36.46, 22.26, 11.48. HRMS: *m*/*z*: calcd for C_10_H_16_N_5_O^+^: 222.1349 [M + H]^+^; found: 222.1349.


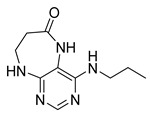


4-(Hexylamino)-5,7,8,9-tetrahydro-6*H*-pyrimido[4,5-b][1,4]diazepin-6-one (**3b**). White solid. Yield: 42% (12.5 mg). ^1^H-NMR (500 MHz, DMSO-*d*_6_) δ 8.40 (s, 1H), 7.75 (s, 1H), 6.67 (t, *J* = 4.2 Hz, 1H), 6.33 (t, *J* = 5.3 Hz, 1H), 3.50 (td, *J* = 4.7, 2.2 Hz, 2H), 3.27 (td, *J* = 7.1, 5.7 Hz, 2H), 2.49–2.46 (m, 2H, overlapped with DMSO), 1.55–1.47 (m, 2H), 1.35–1.25 (m, 6H), 0.86 (t, *J* = 6.9 Hz, 3H). ^13^C-NMR (126 MHz, DMSO-*d*_6_) δ 172.71, 155.16, 153.12, 152.96, 96.96, 43.18, 40.86, 36.47, 31.12, 29.02, 26.18, 22.08, 13.92. HRMS: *m*/*z*: calcd for C_13_H_22_N_5_O^+^: 264.1819 [M + H]^+^; found: 264.1816.


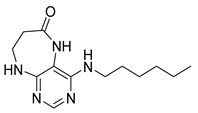


4-(Diethylamino)-5,7,8,9-tetrahydro-6*H*-pyrimido[4,5-b][1,4]diazepin-6-one (**3c**). White solid. Yield: 55% (15.1 mg). ^1^H-NMR (500 MHz, DMSO-*d*_6_) δ 8.24 (s, 1H), 7.84 (s, 1H), 6.92 (t, *J* = 3.3 Hz, 1H), 3.58–3.52 (m, 2H), 3.34–3.26 (m, 4H, overlapped with residual water), 2.59–2.53 (m, 2H), 1.06 (t, *J* = 7.0 Hz, 6H). ^13^C-NMR (126 MHz, DMSO-*d*_6_) δ 171.73, 158.17, 156.14, 152.79, 101.02, 43.56, 43.29, 35.09, 13.19. HRMS: *m*/*z*: calcd for C_11_H_18_N_5_O^+^: 236.1506 [M + H]^+^; found: 236.1505.


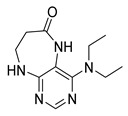


4-(Benzylamino)-5,7,8,9-tetrahydro-6*H*-pyrimido[4,5-b][1,4]diazepin-6-one (**3d**). White solid. Yield: 45% (13.8 mg). ^1^H-NMR (500 MHz, DMSO-*d*_6_) δ 8.49 (s, 1H), 7.75 (s, 1H), 7.36–7.32 (m, 2H), 7.32–7.28 (m, 2H), 7.24–7.20 (m, 1H), 6.92 (t, *J* = 5.7 Hz, 1H), 6.76 (t, *J* = 4.2 Hz, 1H), 4.52 (d, *J* = 5.7 Hz, 2H), 3.54–3.50 (m, 2H), 2.53–2.50 (m, 2H, overlapped with DMSO). ^13^C-NMR (126 MHz, DMSO-*d*_6_) δ 172.70, 155.02, 153.34, 152.93, 140.09, 128.06, 127.44, 126.52, 97.15, 44.16, 43.12, 36.46. HRMS: *m*/*z*: calcd for C_14_H_16_N_5_O^+^: 270.1349 [M + H]^+^; found: 270.1346.


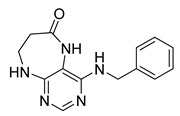


4-(Cyclohexylamino)-5,7,8,9-tetrahydro-6*H*-pyrimido[4,5-b][1,4]diazepin-6-one (**3e**). White solid. Yield: 40% (11.8 mg). ^1^H-NMR (500 MHz, DMSO-*d*_6_) δ 8.53 (s, 1H), 7.74 (s, 1H), 6.68 (t, *J* = 4.2 Hz, 1H), 6.06 (d, *J* = 7.4 Hz, 1H), 3.82 (dtd, *J* = 10.6, 7.0, 3.8 Hz, 1H), 3.50 (td, *J* = 4.7, 2.1 Hz, 2H), 2.50–2.47 (m, 2H, overlapped with DMSO), 1.93–1.83 (m, 2H), 1.78–1.67 (m, 2H), 1.62–1.55 (m, 1H), 1.33–1.08 (m, 5H). ^13^C-NMR (126 MHz, DMSO-*d*_6_) δ 172.78, 154.23, 153.21, 152.87, 96.87, 49.28, 43.13, 36.60, 32.59, 25.41, 24.82. HRMS: *m*/*z*: calcd for C_13_H_20_N_5_O^+^: 262.1662 [M + H]^+^; found: 262.1663.


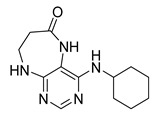


4-(Cyclooctylamino)-5,7,8,9-tetrahydro-6*H*-pyrimido[4,5-b][1,4]diazepin-6-one (**3f**). White solid. Yield: 38% (12.3 mg). ^1^H-NMR (500 MHz, DMSO-*d*_6_) δ 8.57 (s, 1H), 7.75 (s, 1H), 6.66 (t, *J* = 4.2 Hz, 1H), 6.07 (d, *J* = 7.5 Hz, 1H), 4.14–4.04 (m, 1H), 3.53–3.47 (m, 2H), 2.50–2.46 (m, 2H, overlapped with DMSO), 1.82–1.73 (m, 2H), 1.72–1.42 (m, 12H). ^13^C-NMR (126 MHz, DMSO-*d*_6_) δ 172.81, 153.98, 153.15, 152.89, 96.97, 50.13, 43.16, 36.63, 31.44, 26.97, 25.05, 23.34. HRMS: *m*/*z*: calcd for C_15_H_24_N_5_O^+^: 290.1975 [M + H]^+^; found: 290.1974.


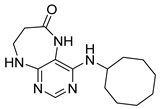


4-(Piperidin-1-yl)-5,7,8,9-tetrahydro-6*H*-pyrimido[4,5-b][1,4]diazepin-6-one (**3g**). White solid. Yield: 46% (13.2 mg). ^1^H-NMR (500 MHz, DMSO-*d*_6_) δ 8.14 (s, 1H), 7.87 (s, 1H), 7.05 (t, *J* = 3.2 Hz, 1H), 3.56–3.50 (m, 2H), 3.20–3.14 (m, 4H), 2.58–2.54 (m, 2H), 1.62–1.53 (m, 6H). ^13^C-NMR (126 MHz, DMSO-*d*_6_) δ 171.75, 159.07, 155.65, 152.81, 102.31, 48.65, 42.67, 35.64, 25.24, 24.14. HRMS: *m*/*z*: calcd for C_12_H_18_N_5_O^+^: 248.1506 [M + H]^+^; found: 248.1506.


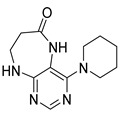


4-Morpholino-5,7,8,9-tetrahydro-6*H*-pyrimido[4,5-b][1,4]diazepin-6-one (**3h**). White solid. Yield: 51% (14.7 mg). ^1^H-NMR (500 MHz, DMSO-*d*_6_) δ 8.39 (s, 1H), 7.89 (s, 1H), 7.13 (t, *J* = 3.2 Hz, 1H), 3.72–3.66 (m, 4H), 3.58–3.51 (m, 2H), 3.26–3.19 (m, 4H), 2.59–2.53 (m, 2H). ^13^C-NMR (126 MHz, DMSO-*d*_6_) δ 171.88, 158.27, 155.88, 152.84, 102.30, 65.80, 47.87, 42.81, 35.57. HRMS: *m*/*z*: calcd for C_11_H_16_N_5_O_2_^+^: 250.1299 [M + H]^+^; found: 250.1297.


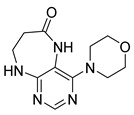


## 4. Conclusions

In conclusion, we have developed an efficient solid-phase synthetic approach leading to various dihydropteridinones, tetrahydropyrrolopteridinones, or pyrimidodiazepinones using one versatile building block. The reduction and cyclization were performed after cleavage from polymer support; however, crude products were obtained after simple filtration of powdered zinc and evaporation of solvents. Final cyclization leading to dihydropteridinones and tetrahydropyrrolopteridinones proceeded smoothly at room temperature. On the other hand, the cyclization of β-alanine precursors had to be accelerated by heating to 80 °C. In summary, we prepared forty heterocycles utilizing one versatile building block modified with other distinct substituents. All derivatives were fully characterized and might be used for future SAR studies.

## Figures and Tables

**Table 1:** Overview of synthesized dihydropteridinones **1**.

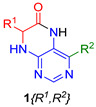					
Product	R^1^	R^2^	Crude Purity ^a^ [%]	Final Purity ^b^ [%]	Overall Yield ^c^ [%]
**1a**	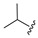	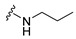	92	98	87
**1b**	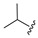	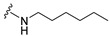	89	99	67
**1c**	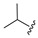	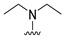	78	99	74
**1d**	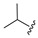	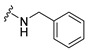	84	98	75
**1e**	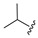	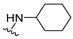	85	97	66
**1f**	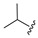	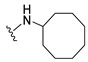	88	96	91
**1g**	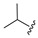	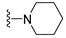	75	97	90
**1h**	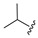	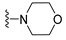	88	98	92
**1i**	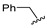	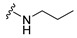	98	98	89
**1j**	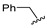	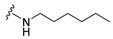	74	99	76
**1k**	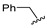	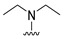	78	99	91
**1l**	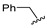	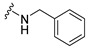	84	99	80
**1m**	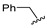	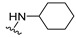	77	98	82
**1n**	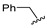	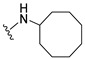	78	98	92
**1o**	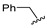	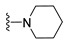	77	98	89
**1p**	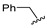	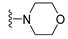	80	98	70
**1q**	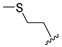	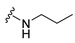	95	97	68
**1r**	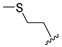	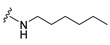	72	98	66
**1s**	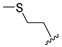	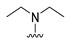	69	99	76
**1t**	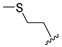	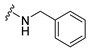	90	98	41
**1u**	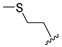	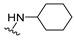	80	97	79
**1v**	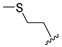	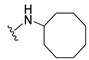	88	97	60
**1w**	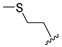	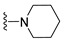	82	98	63
**1x**	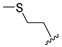	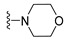	73	97	65

^a^ Overall purity of crude products estimated from LC-MS traces at 210–400 nm; ^b^ Determined from the HPLC-UV traces after purification (210−400 nm); and ^c^ Isolated yield after purification.

**Table 2:** Overview of synthesized tetrahydropyrrolopteridinones **2**.

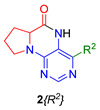				
Product	R^2^	Crude Purity ^a^ [%]	Final Purity ^b^ [%]	Overall Yield ^c^ [%]
**2a**	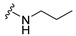	68	98	75
**2b**	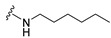	63	99	47
**2c**	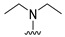	65	98	87
**2d**	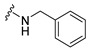	95	98	81
**2e**	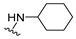	79	97	80
**2f**	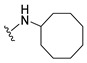	61	97	34
**2g**	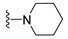	71	98	60
**2h**	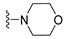	74	99	57

^a^ Overall purity of crude products estimated from LC-MS traces at 210–400 nm; ^b^ Determined from the HPLC-UV traces after purification (210−400 nm); and ^c^ Isolated yield after purification.

**Table 3:** Summary of reaction conditions for cyclization leading to pyrimidodiazepinones **3**.

Entry	R^2^	The Ratio of 17:3 (%) 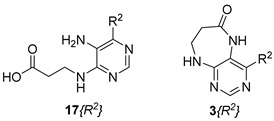		
		rt	50 °C	80 °C
**a**	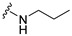	79:21, 16 h	61:39, 16 h	0:100, 3h
**b**	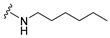	100:0, 16 h	52:48, 16 h	0:100, 3h
**c**	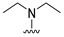	100:0, 16 h	78:22, 16 h	66:34, 3h 0:100, 6h
**d**	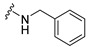	100:0, 16 h	50:50, 16 h	0:100, 3h
**e**	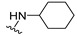	100:0, 16 h	69:31, 16 h	8:92, 3h 0:100, 4h
**f**	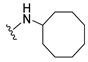	100:0, 16 h	56:44, 16 h	0:100, 3h
**g**	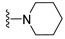	100:0, 16 h	100:0, 16 h	22:78, 3h 0:100, 13h
**h**	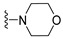	100:0, 16 h	79:21, 16 h	54:46, 3h 0:100, 6h

**Table 4:** Overview of synthesized pyrimidodiazepinones **3**.

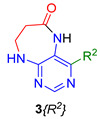				
Product	R^2^	Crude Purity ^a^ [%]	Final Purity ^b^ [%]	Overall Yield ^c^ [%]
**3a**	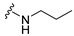	49	98	45
**3b**	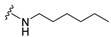	58	98	42
**3c**	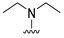	69	99	55
**3d**	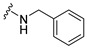	49	97	45
**3e**	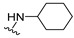	52	98	40
**3f**	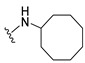	51	98	38
**3g**	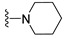	69	99	46
**3h**	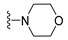	61	99	51

^a^ Overall purity of crude products estimated from LC-MS traces at 210–400 nm; ^b^ Determined from the HPLC-UV traces after purification (210−400 nm); and ^c^ Isolated yield after purification.

## Data Availability

Data is contained within the article.

## Supplementary Materials

The following are available online—copies of ^1^H and ^13^C-NMR.

## Sample Availability

Samples of the compounds are available from the authors.
