# Supplementary material for: Polymer-Supported Synthesis of Various Pteridinones and Pyrimidodiazepinones
Source: Molecules. 2021 Mar 14;26(6):1603. doi: 10.3390/molecules26061603 (PMC8000973; doi:10.3390/molecules26061603)

SI - Copies of ^{1}H and ^{13}C NMR spectra

Polymer-supported synthesis of various pteridinones and pyrimidodiazepinones

Jan Chasák and Lucie Brulíková*
Department of Organic Chemistry, Faculty of Science, Palacký University, 17. listopadu 12, 771 46, Olomouc, Czech Republic
*email: lucie.brulikova@upol.cz

^{1}H and ^{13}C NMR spectra of 7-Isopropyl-4-(propylamino)-7,8-dihydropteridin-6(5H)-one (1a)

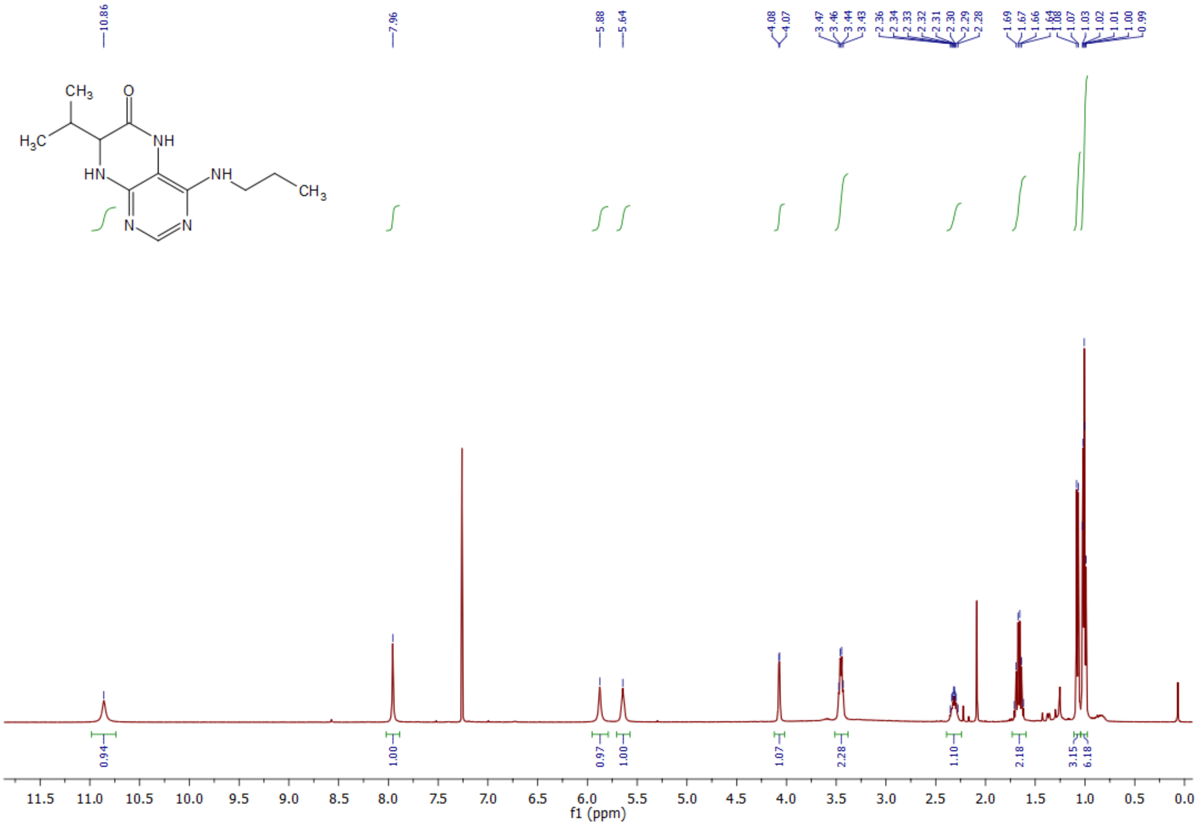

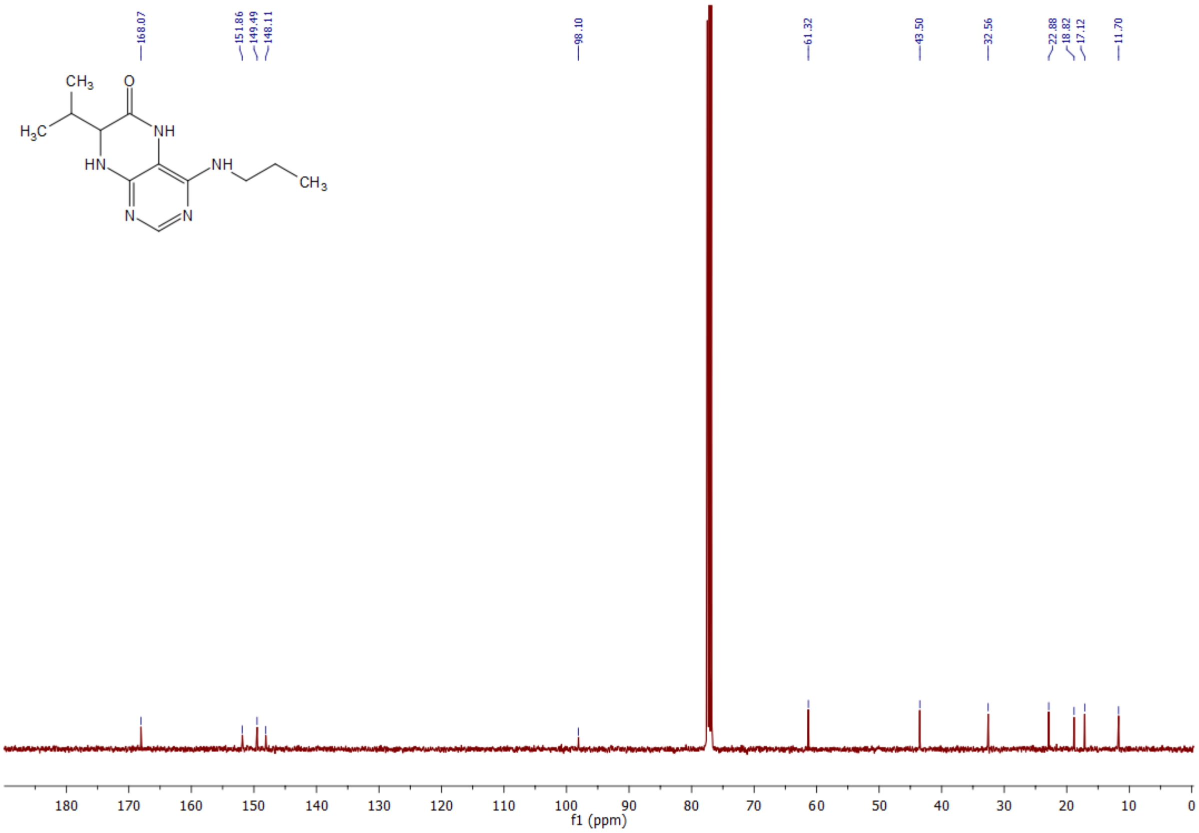

^{1}H and ^{13}C NMR spectra of 4-(Hexylamino)-7-isopropyl-7,8-dihydropteridin-6(5H)-one (1b)

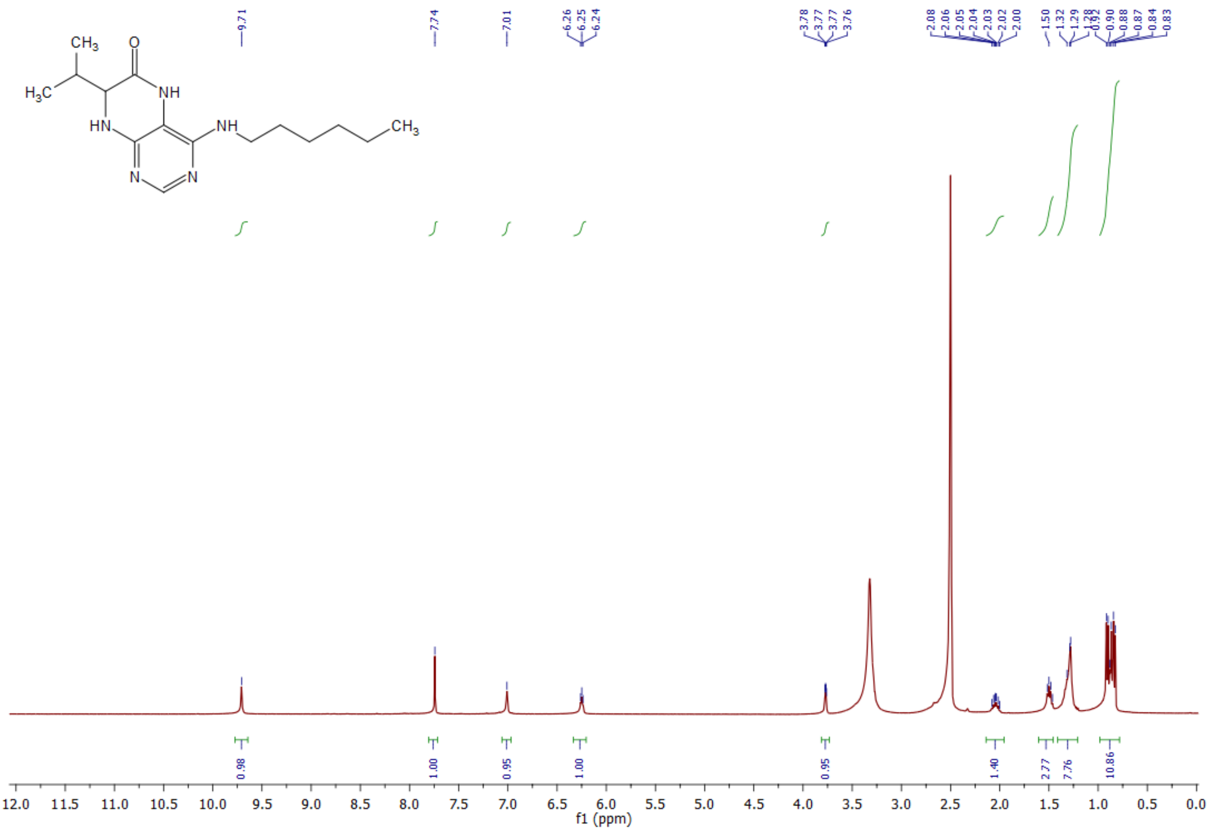

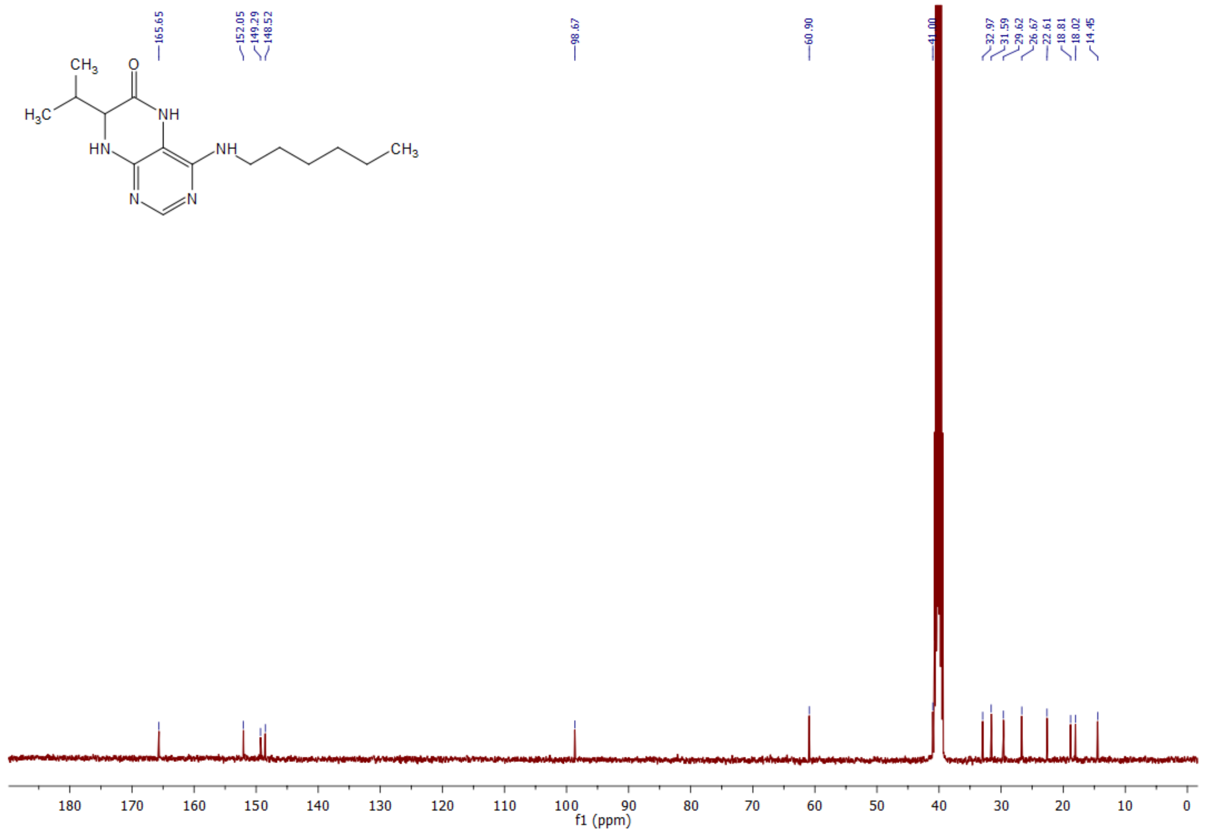

^{1}H and ^{13}C NMR spectra of 4-(Diethylamino)-7-isopropyl-7,8-dihydropteridin-6(5H)-one (1c)

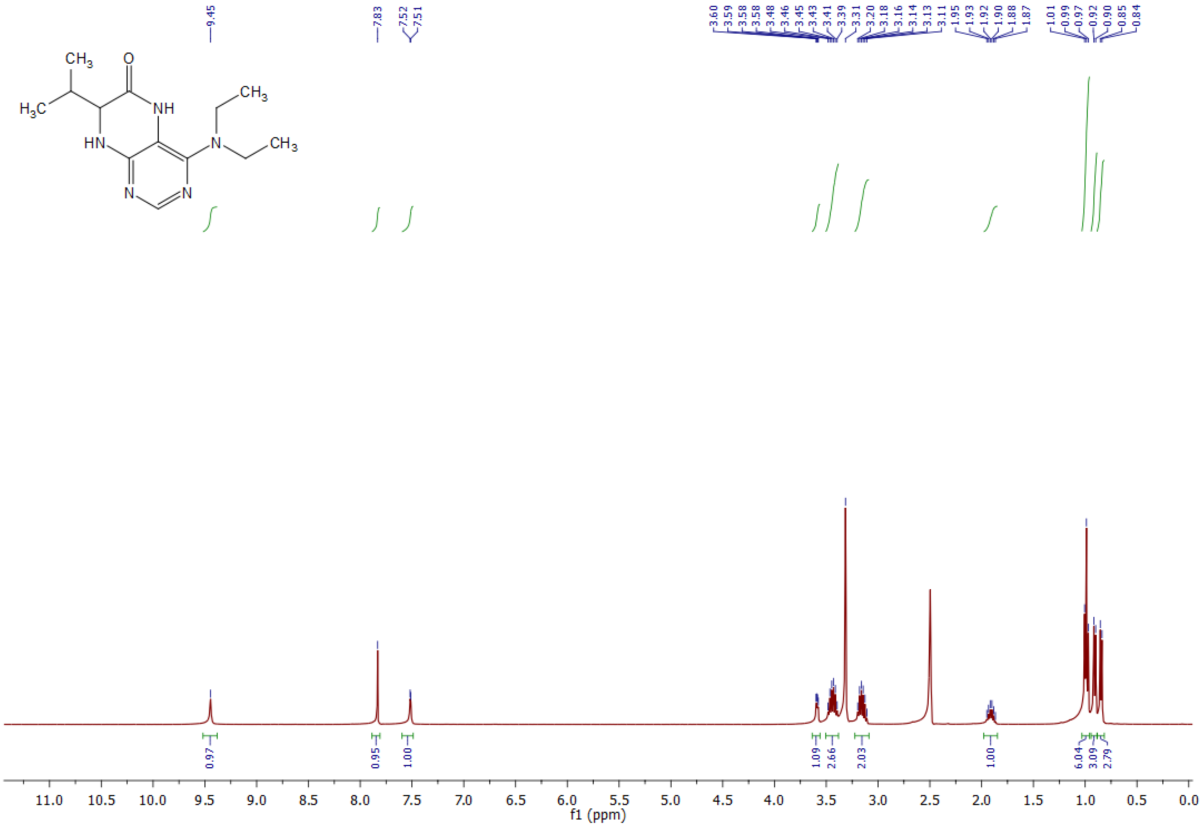

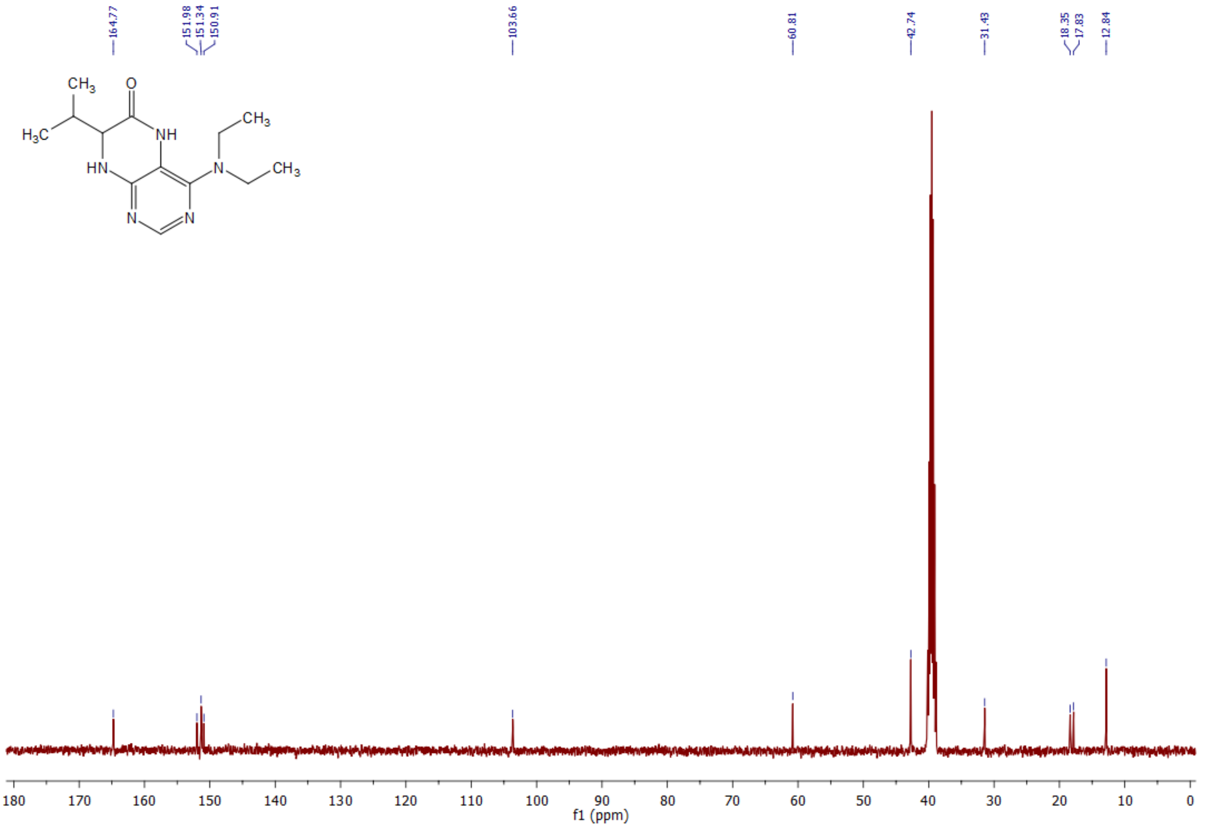

^{1}H and ^{13}C NMR spectra of 4-(Benzylamino)-7-isopropyl-7,8-dihydropteridin-6(5H)-one (1d)

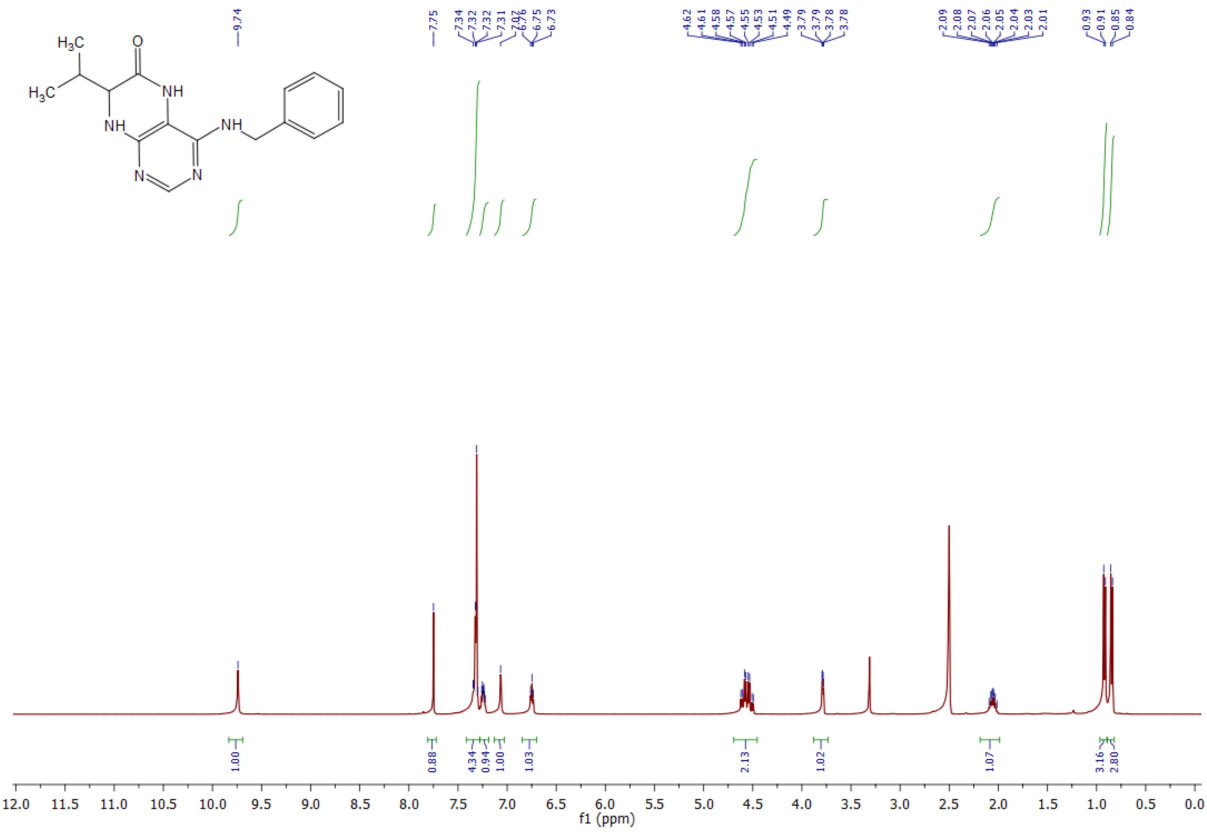

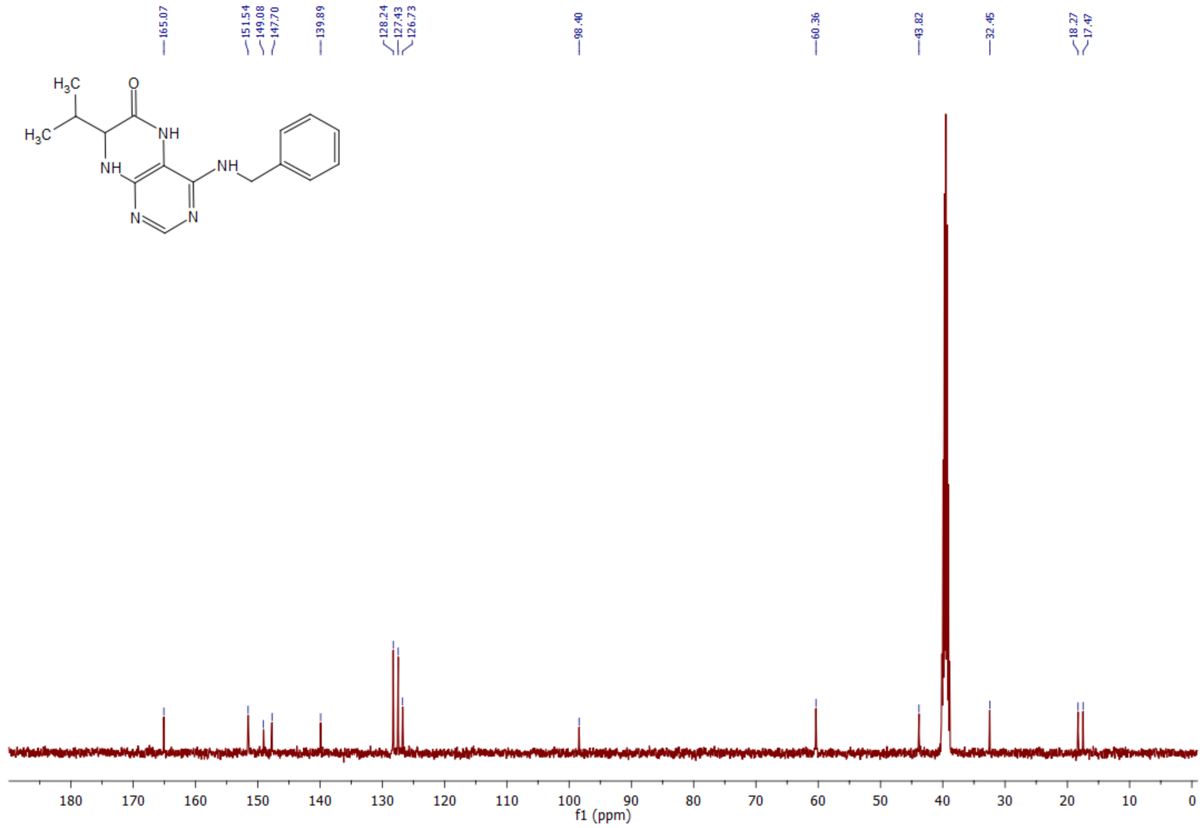

^{1}H and ^{13}C NMR spectra of 4-(Cyclohexylamino)-7-isopropyl-7,8-dihydropteridin-6(5H)-one (1e)

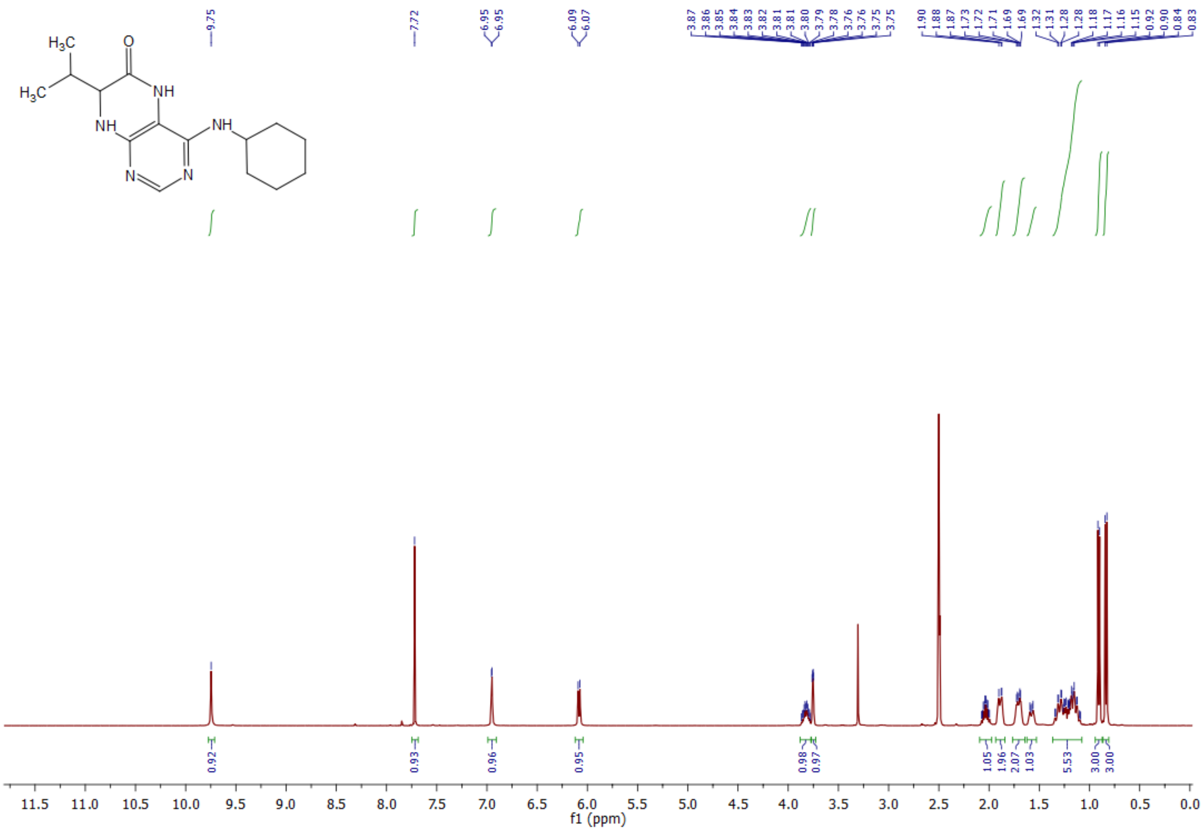

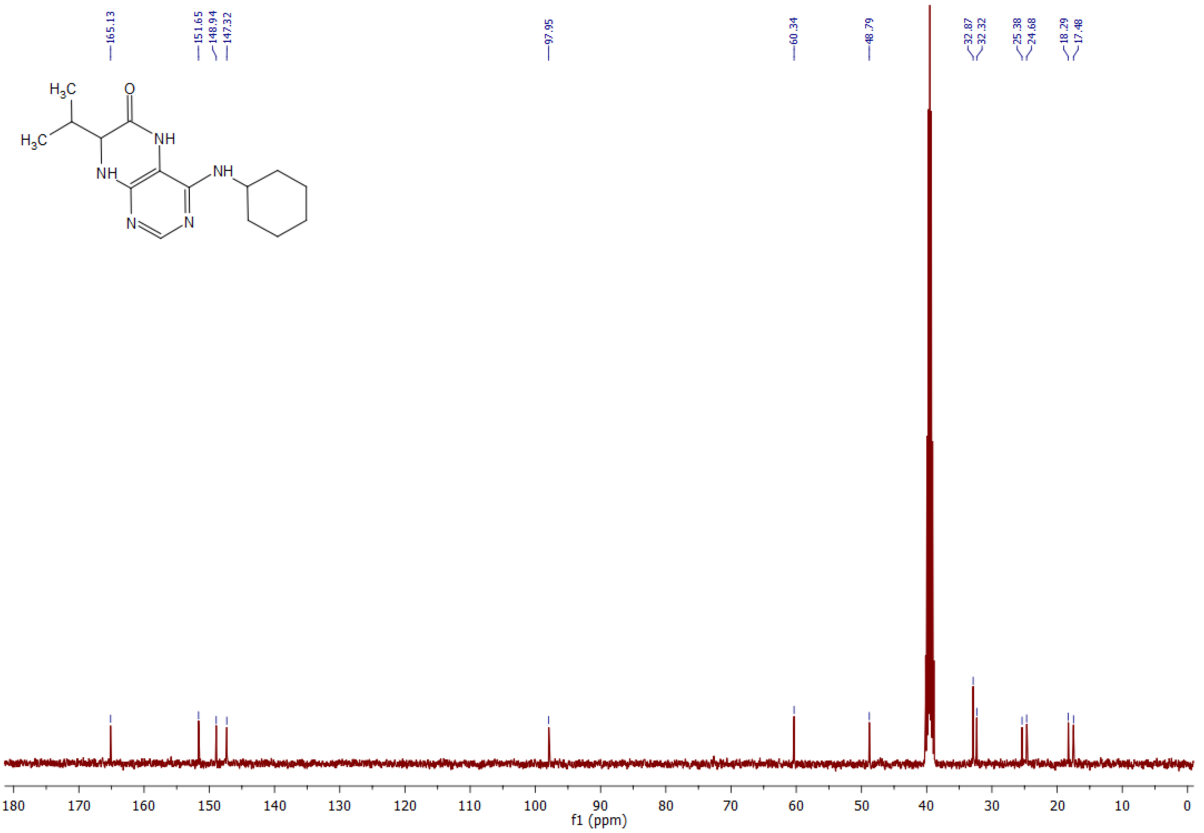

^{1}H and ^{13}C NMR spectra of 4-(Cyclooctylamino)-7-isopropyl-7,8-dihydropteridin-6(5H)-one (1f)

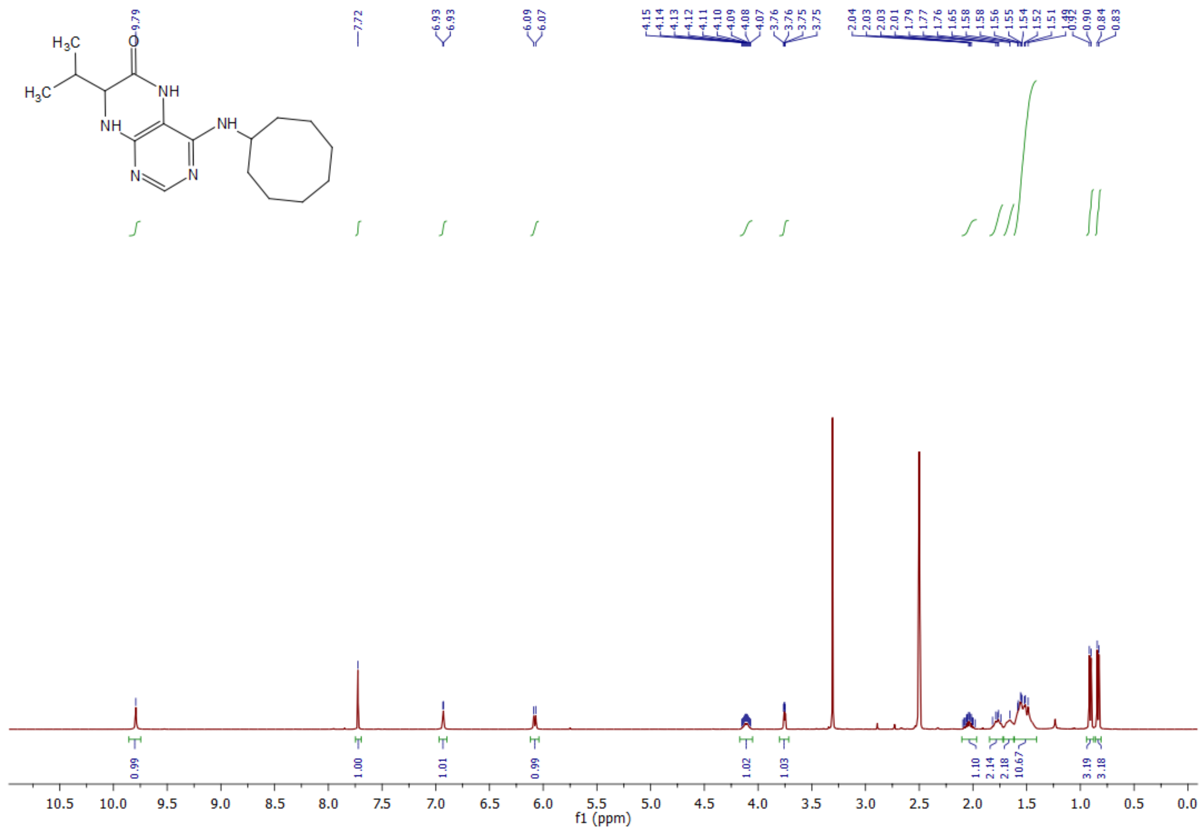

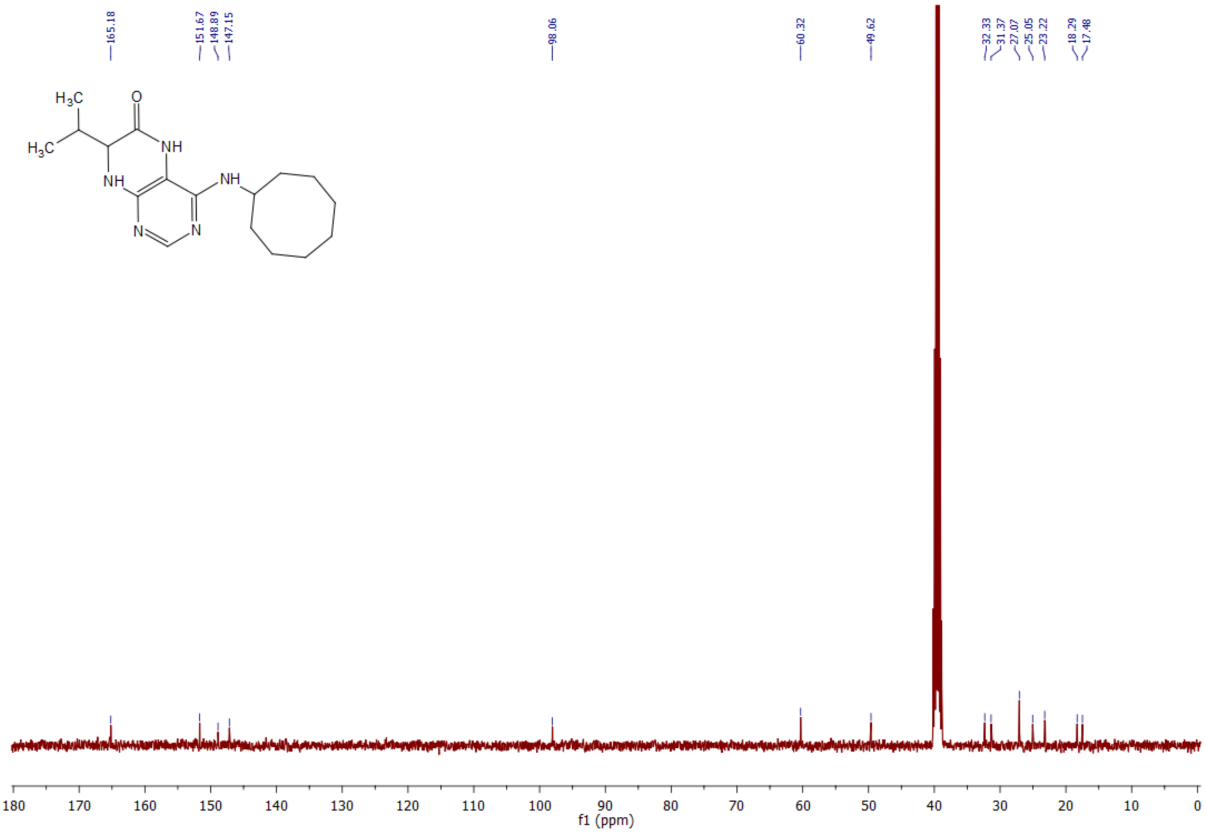

^{1}H and ^{13}C NMR spectra of 7-Isopropyl-4-(piperidin-1-yl)-7,8-dihydropteridin-6(5H)-one (1g)

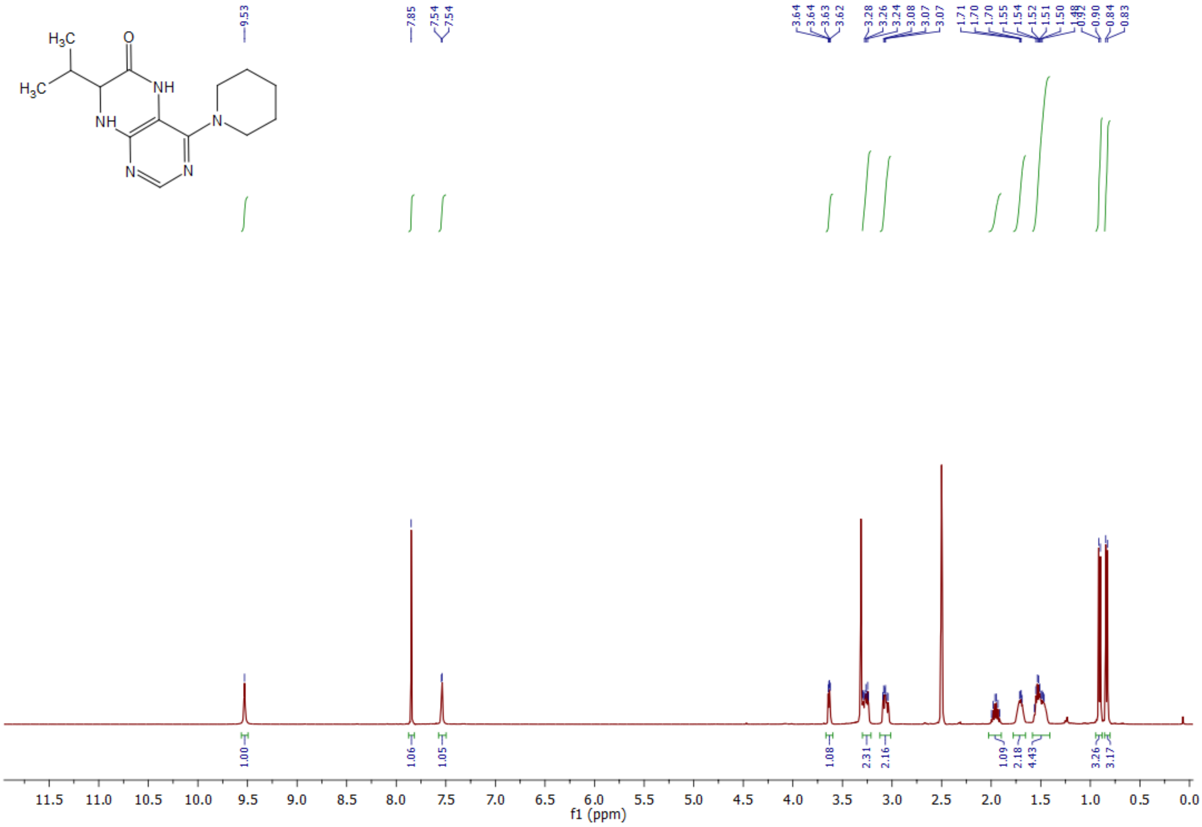

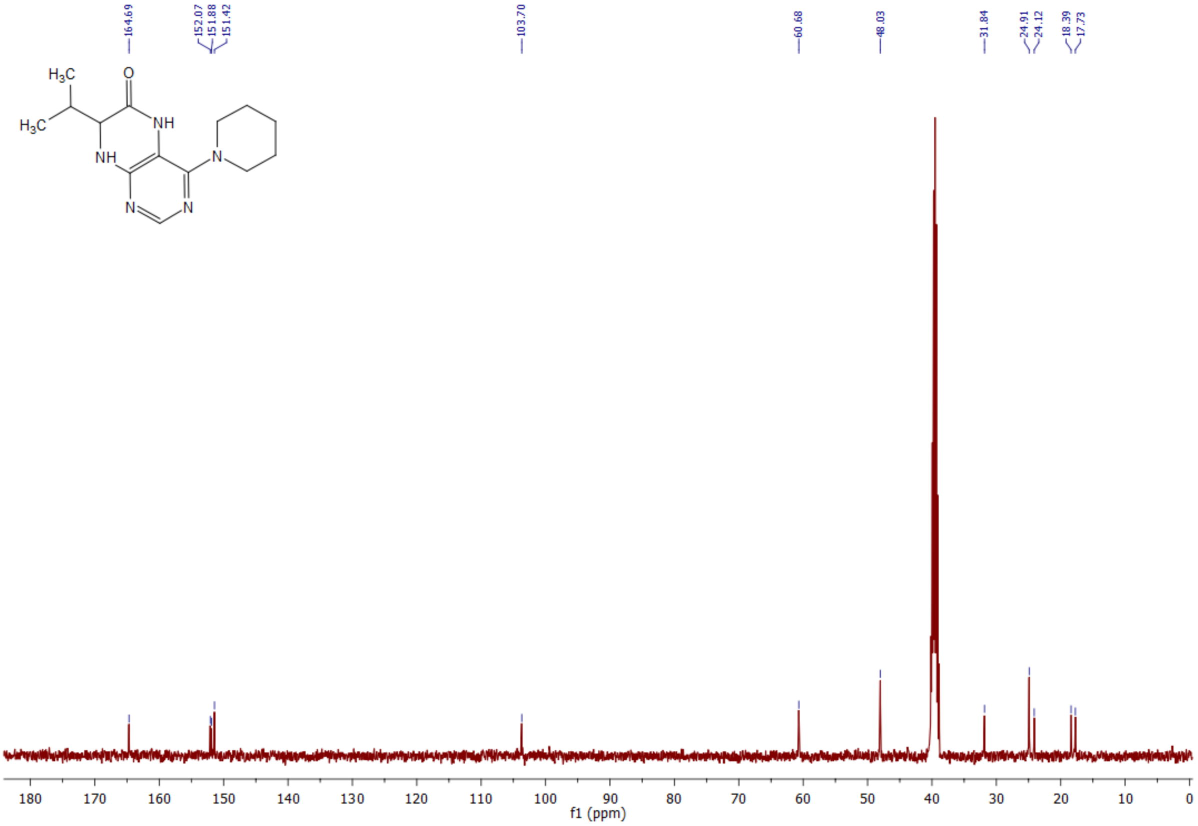

¹H and ¹³C NMR spectra of 7-Isopropyl-4-morpholino-7,8-dihydropteridin-6(5H)-one (1h)

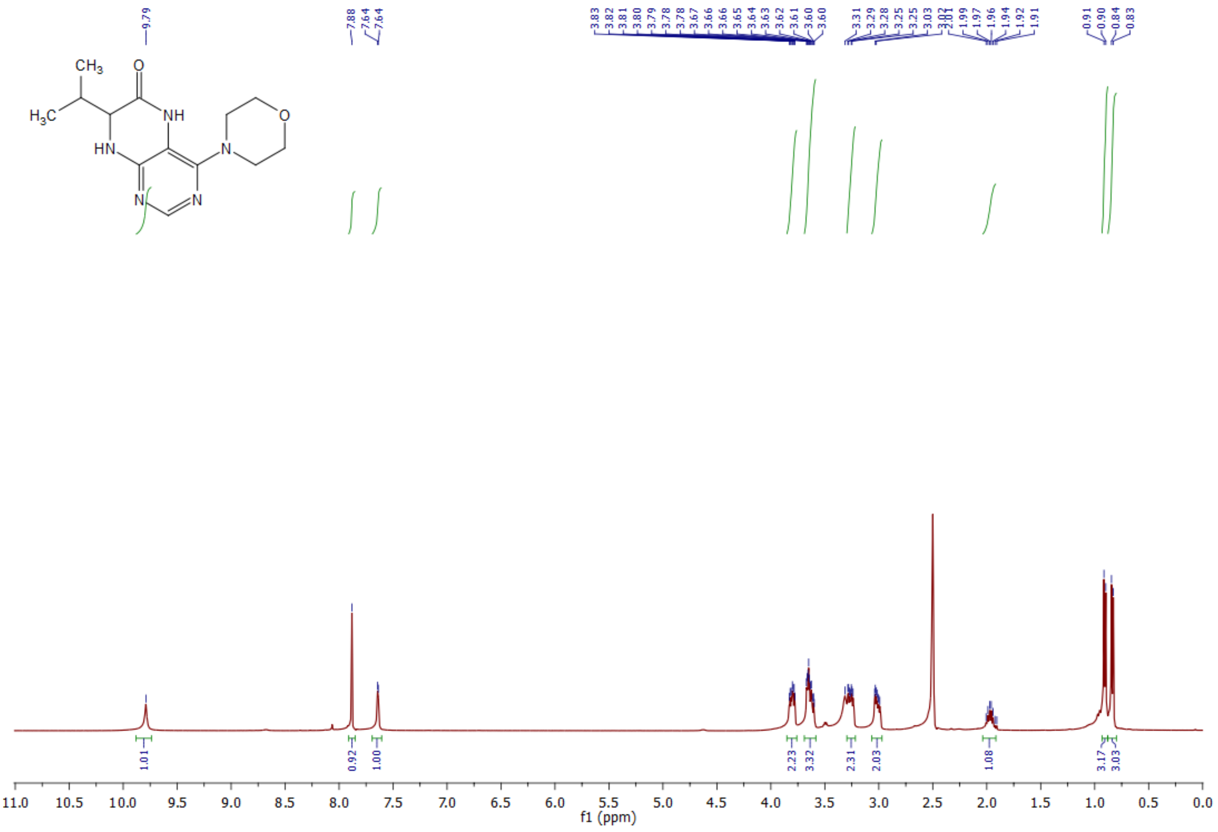

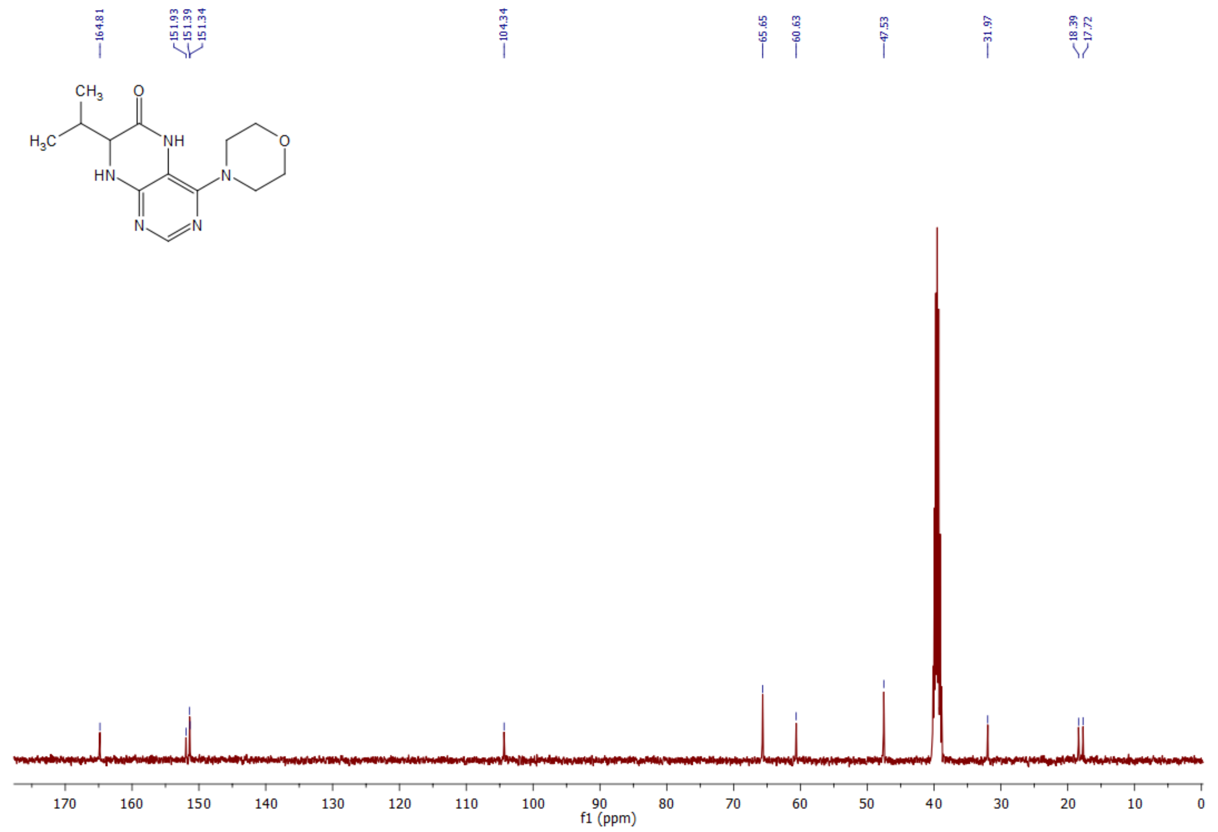

^{1}H and ^{13}C NMR spectra of 7-Benzyl-4-(propylamino)-7,8-dihydropteridin-6(5H)-one (1i)

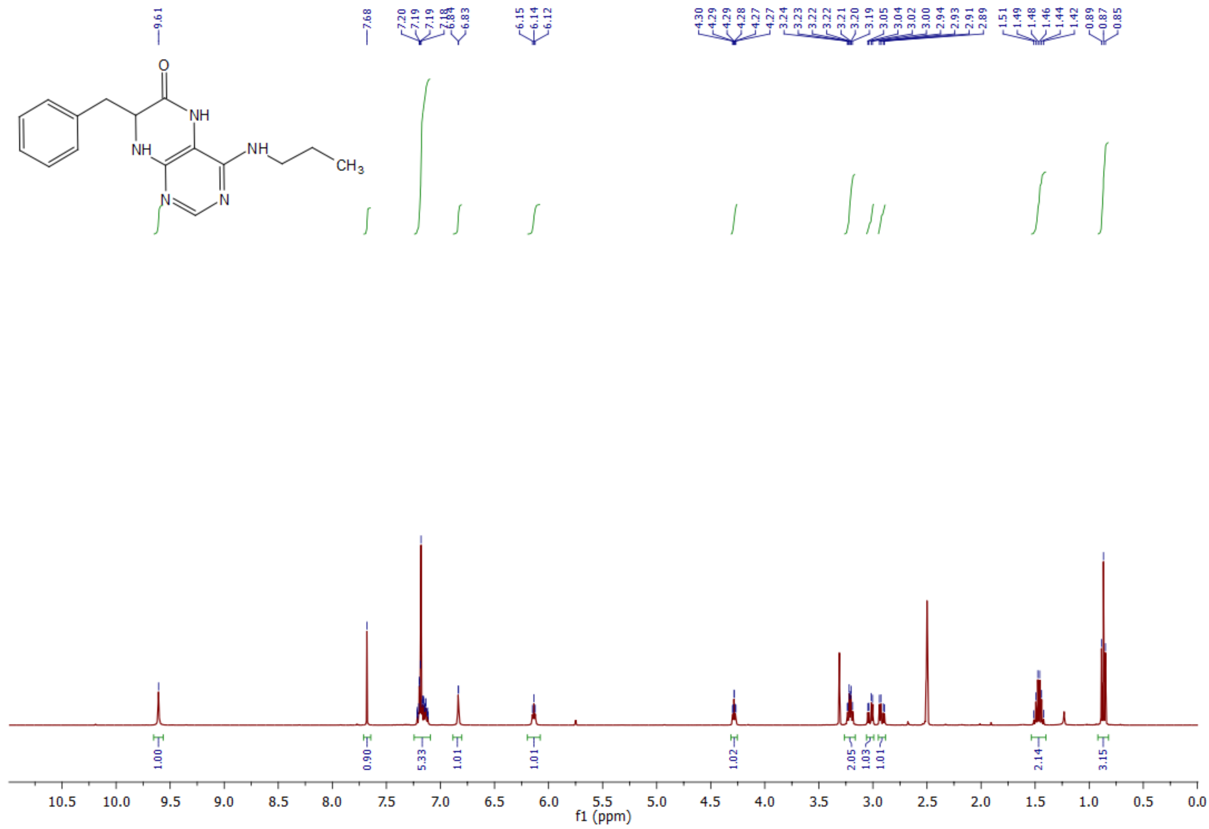

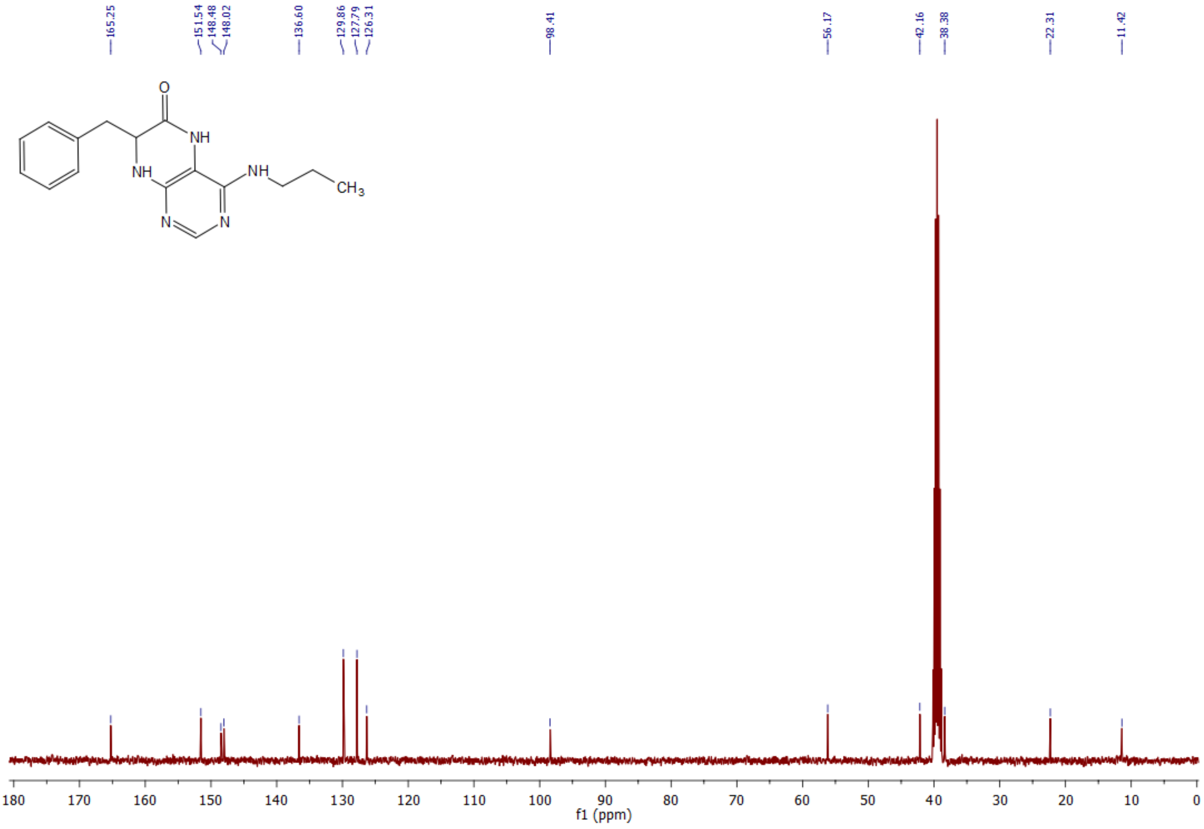

^{1}H and ^{13}C NMR spectra of 7-Benzyl-4-(hexylamino)-7,8-dihydropteridin-6(5H)-one (1j)

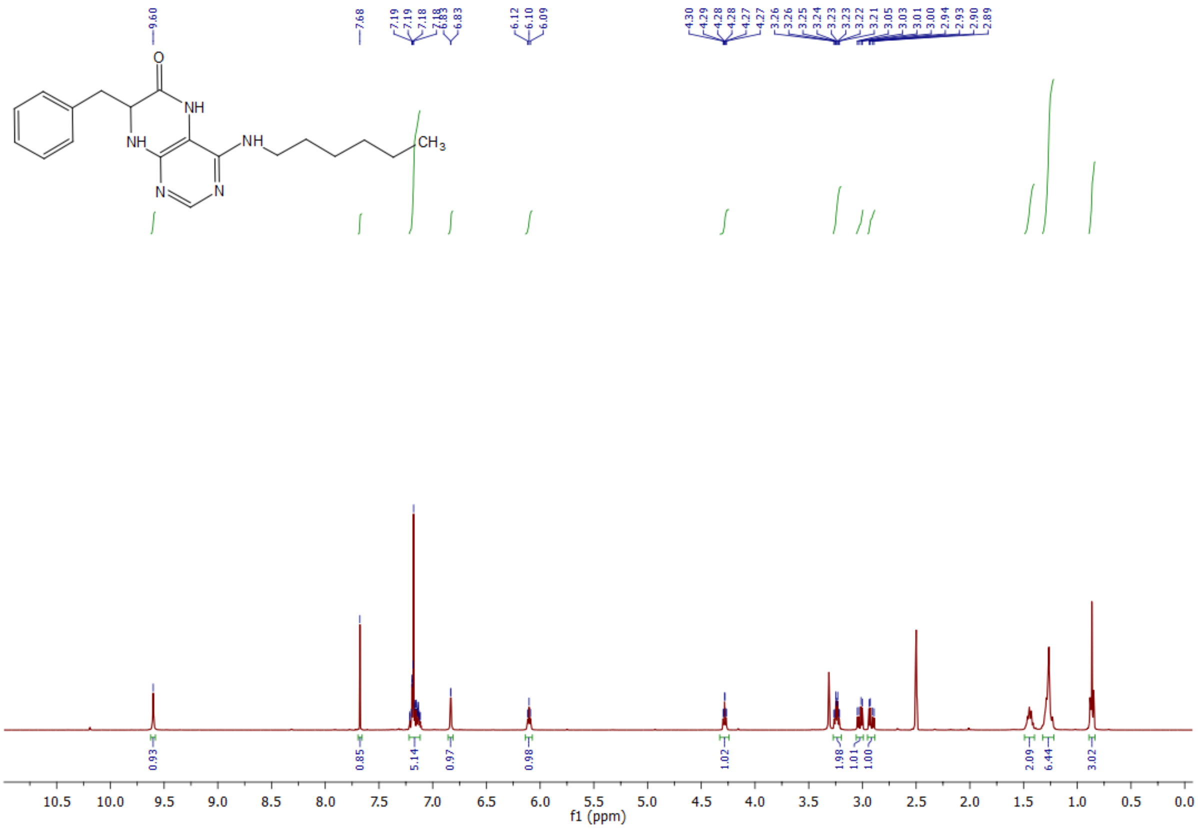

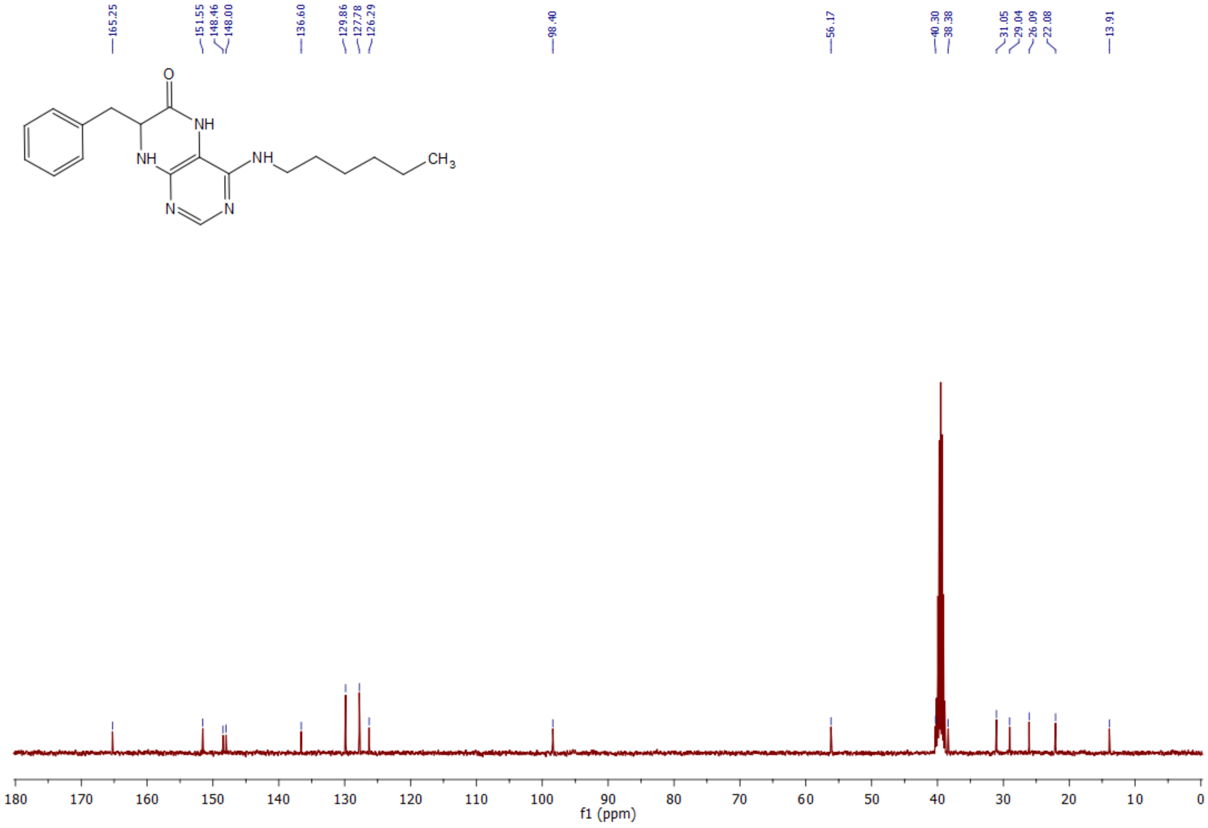

^{1}H and ^{13}C NMR spectra of 7-Benzyl-4-(diethylamino)-7,8-dihydropteridin-6(5H)-one (1k)

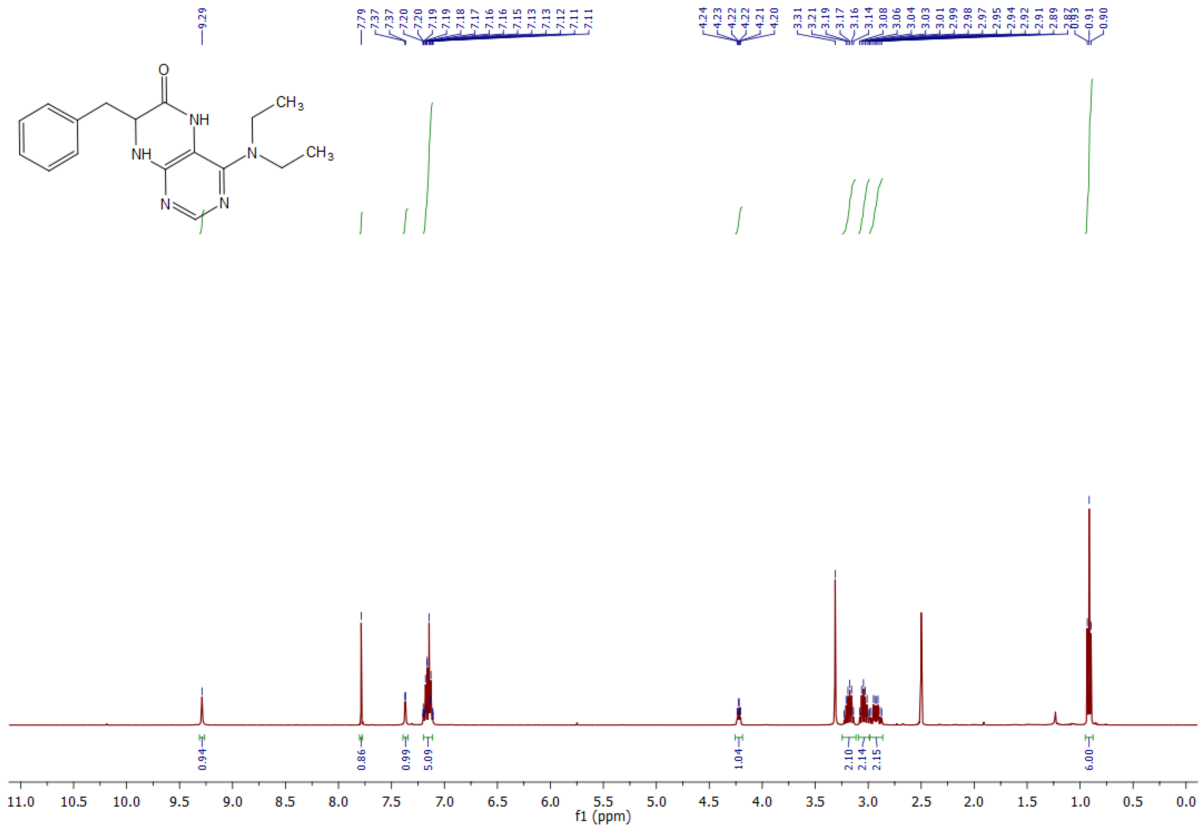

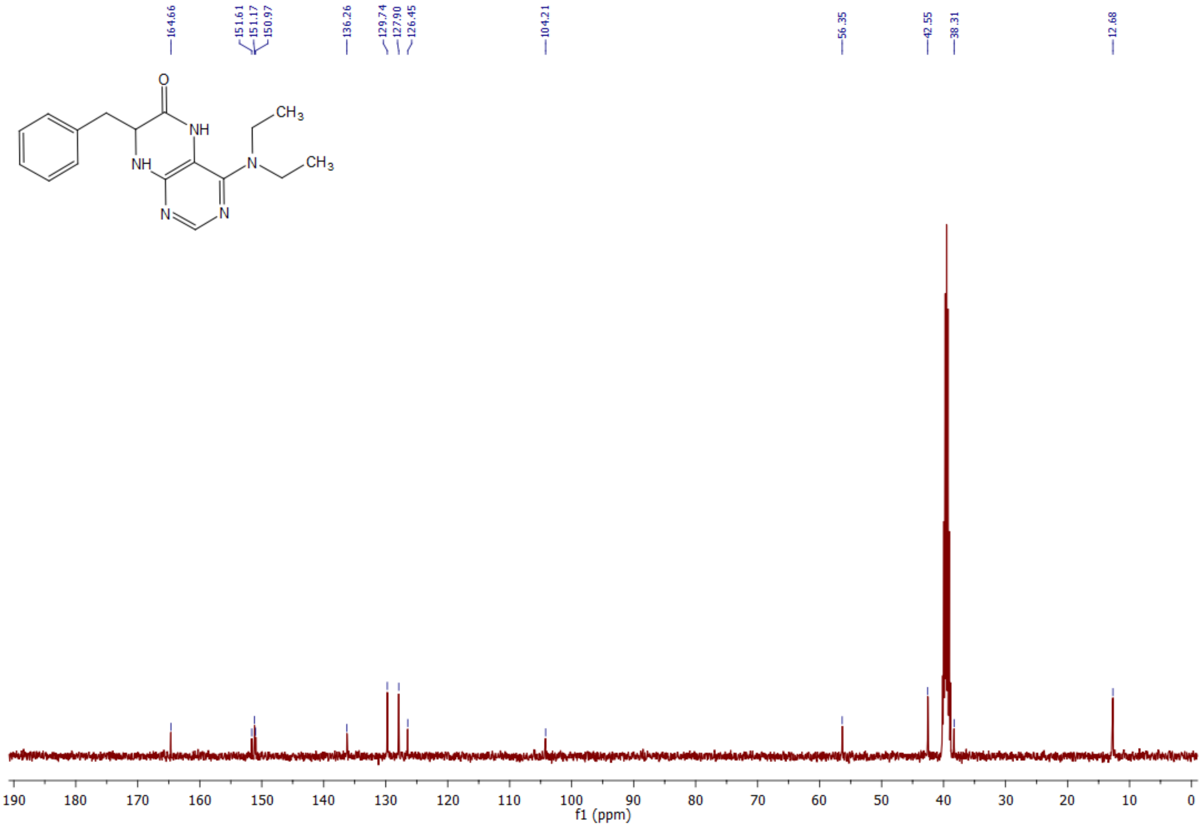

^{1}H and ^{13}C NMR spectra of 7-Benzyl-4-(benzylamino)-7,8-dihydropteridin-6(5H)-one (1l)

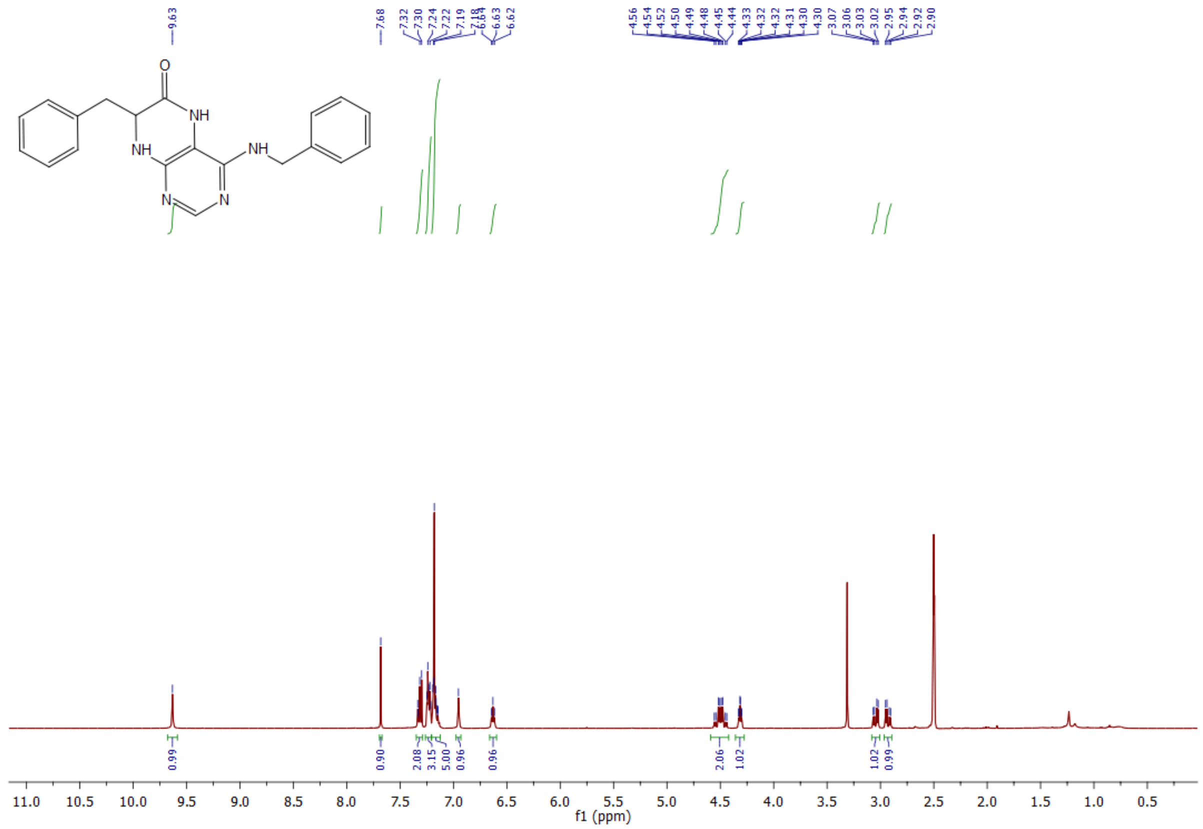

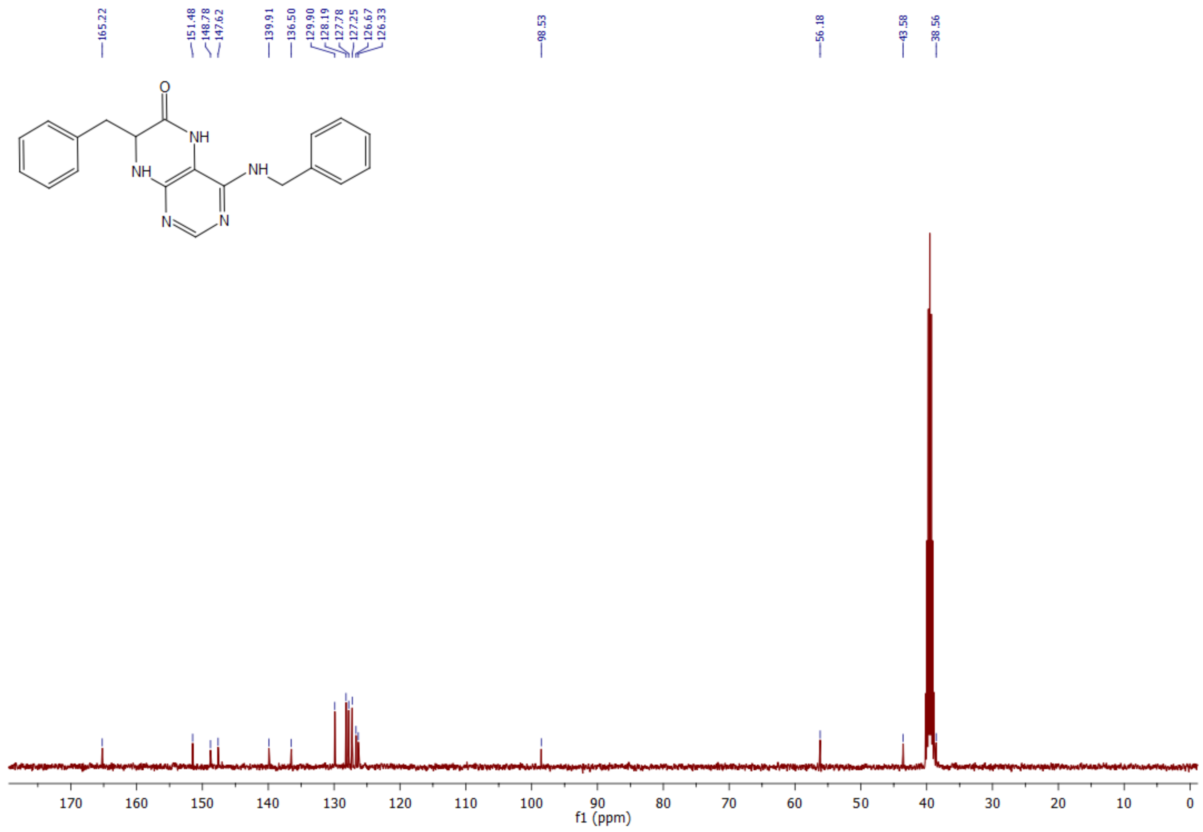

^{1}H and ^{13}C NMR spectra of 7-Benzyl-4-(cyclohexylamino)-7,8-dihydropteridin-6(5H)-one (1m)

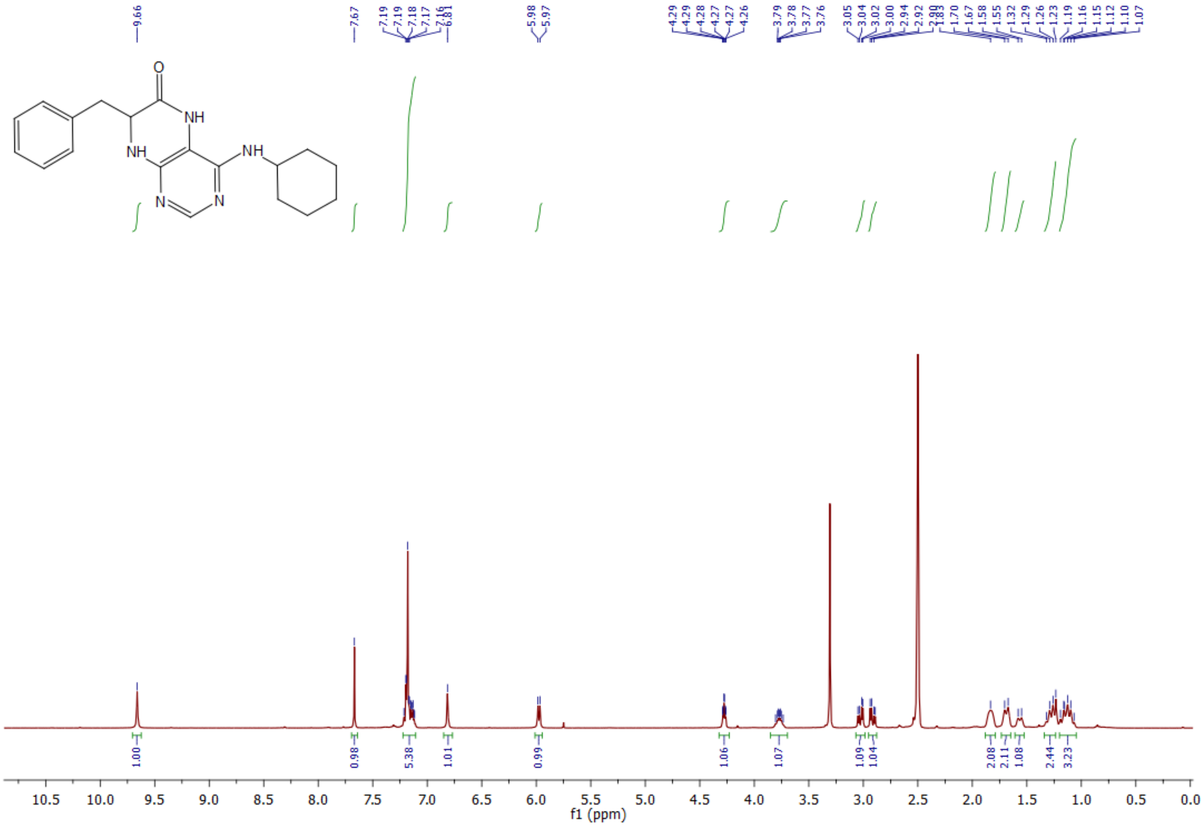

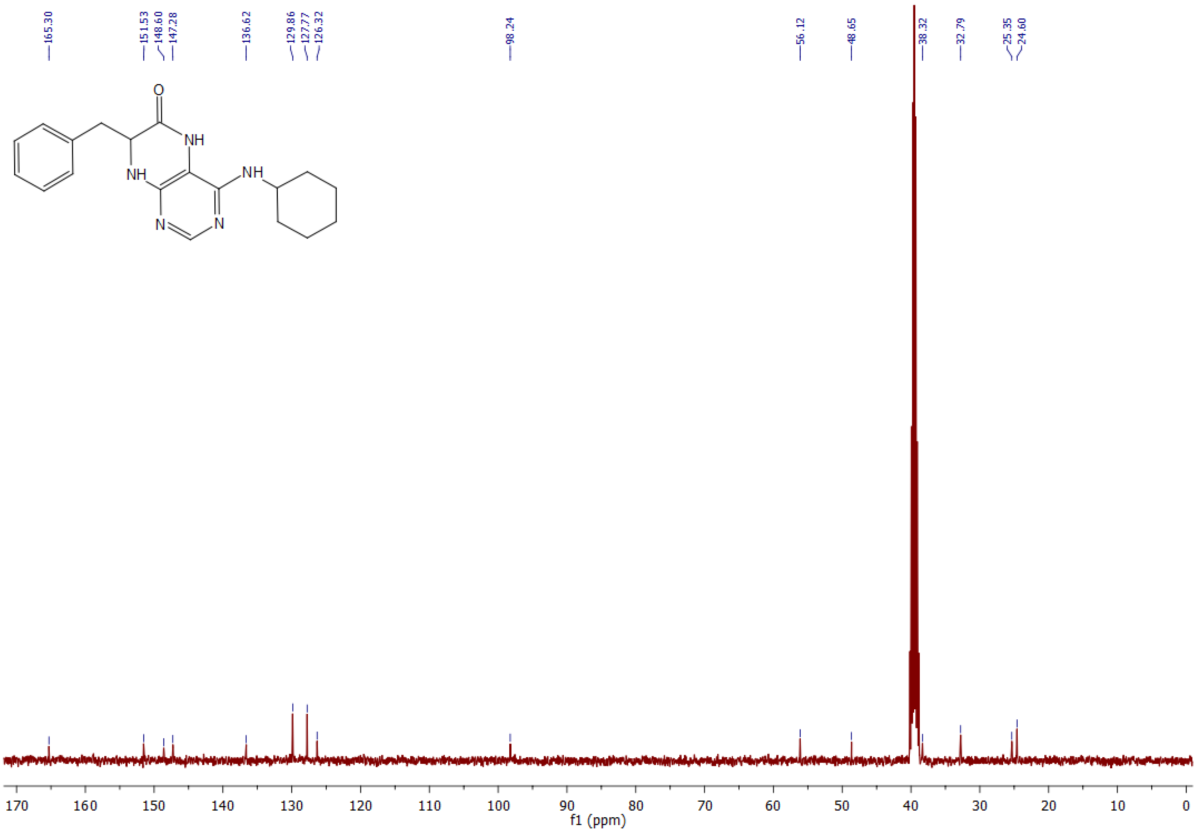

^{1}H and ^{13}C NMR spectra of 7-Benzyl-4-(cyclooctylamino)-7,8-dihydropteridin-6(5H)-one (1n)

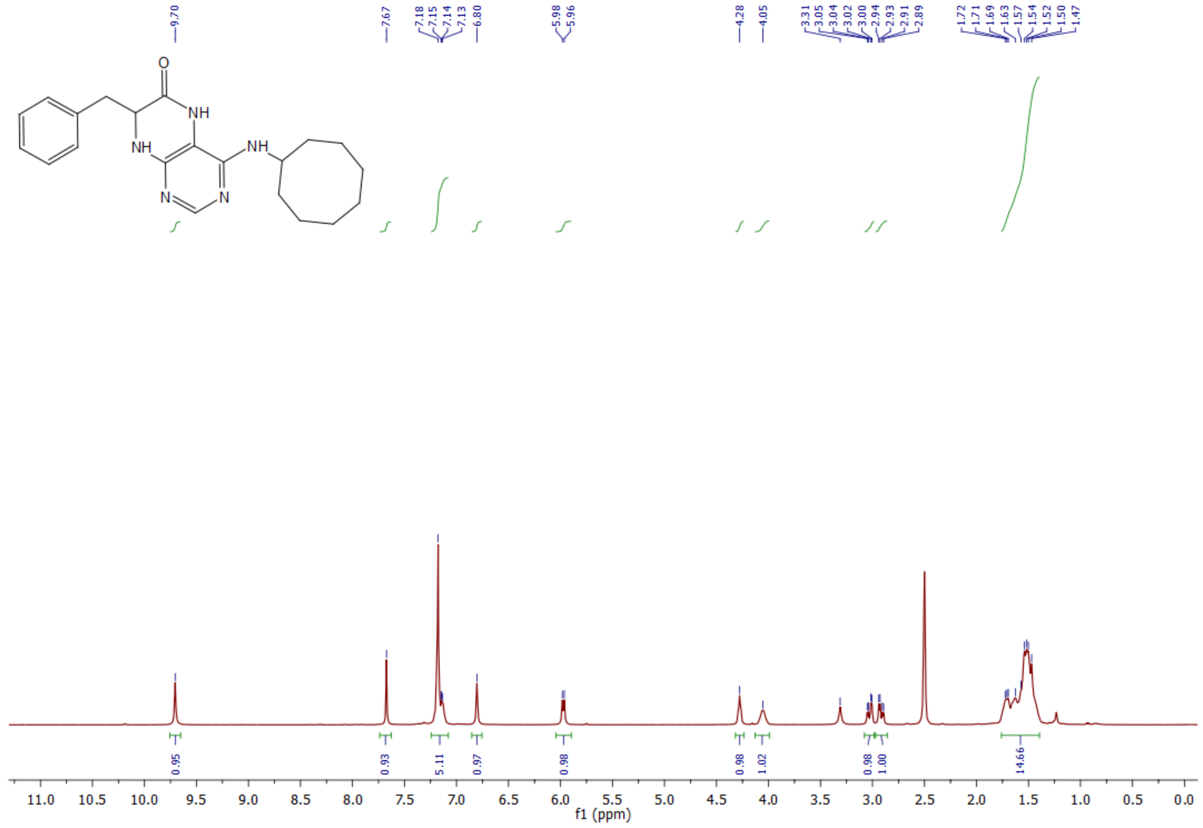

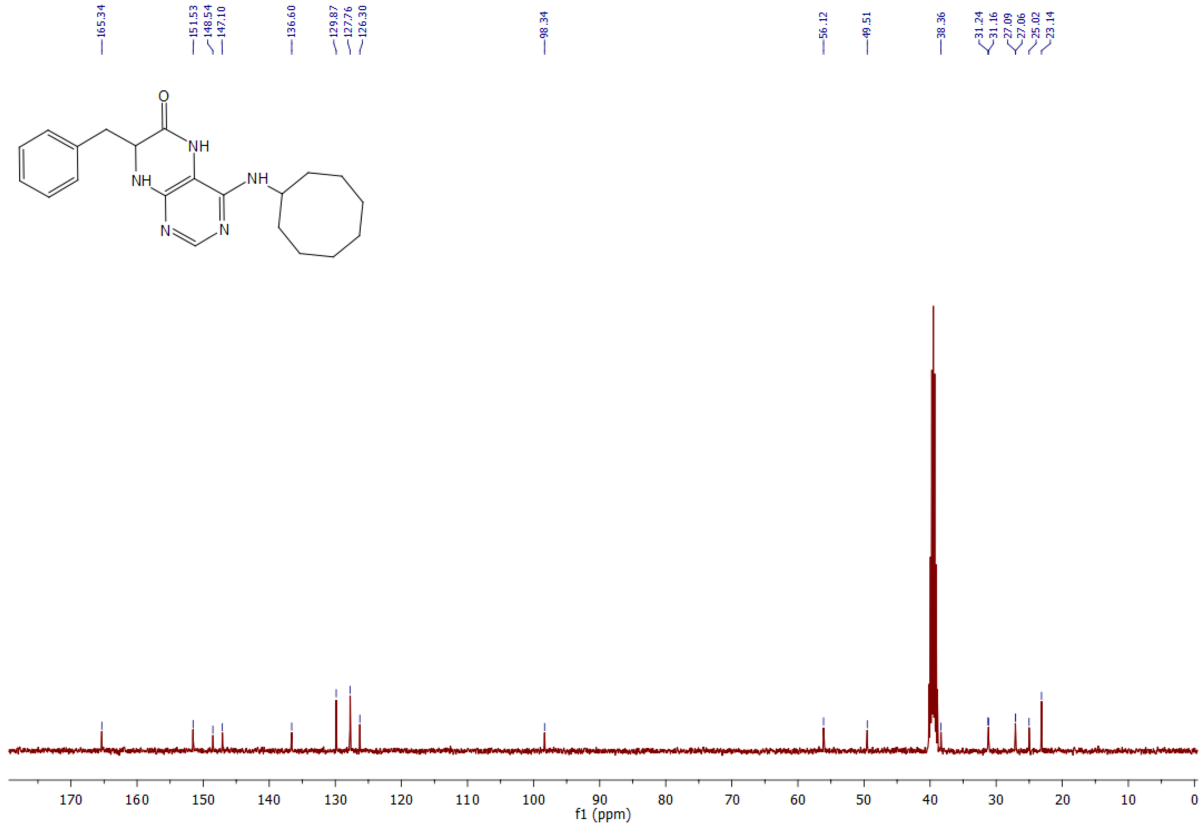

^{1}H and ^{13}C NMR spectra of 7-Benzyl-4-(piperidin-1-yl)-7,8-dihydropteridin-6(5H)-one (1o)

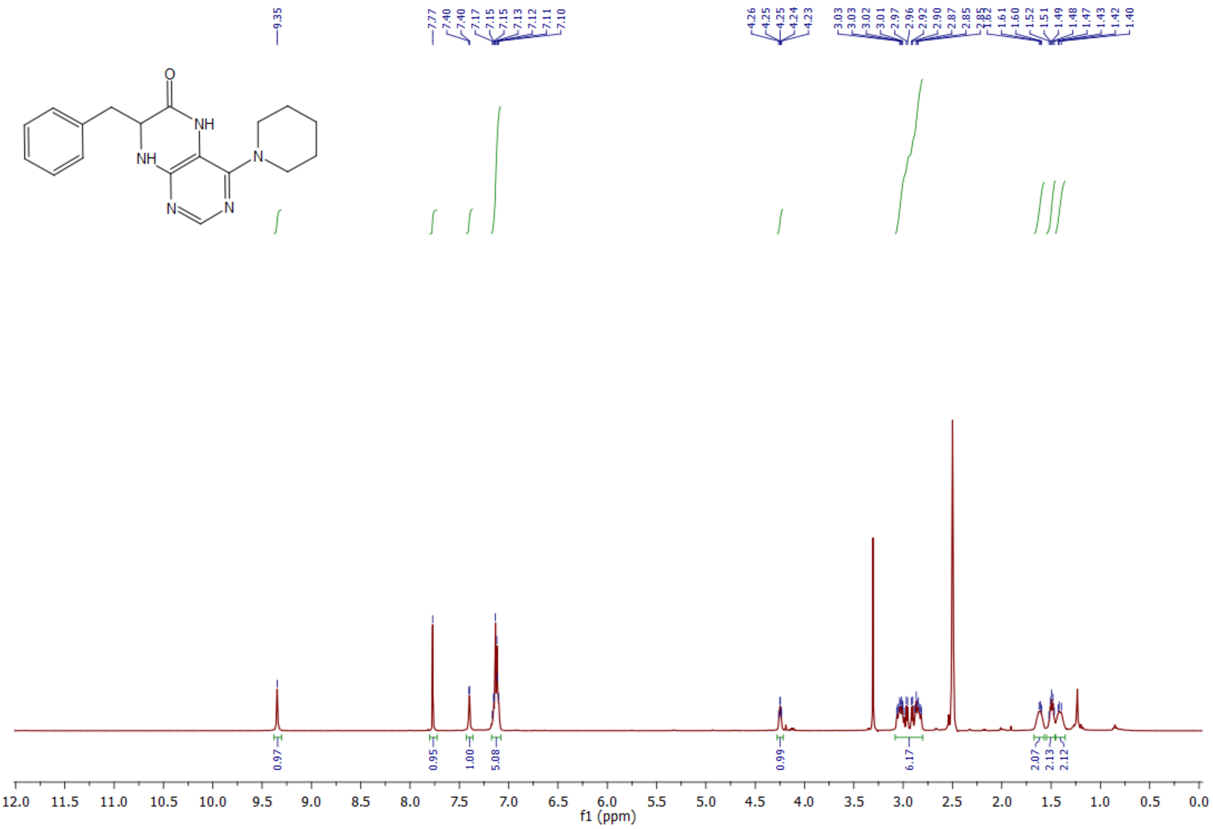

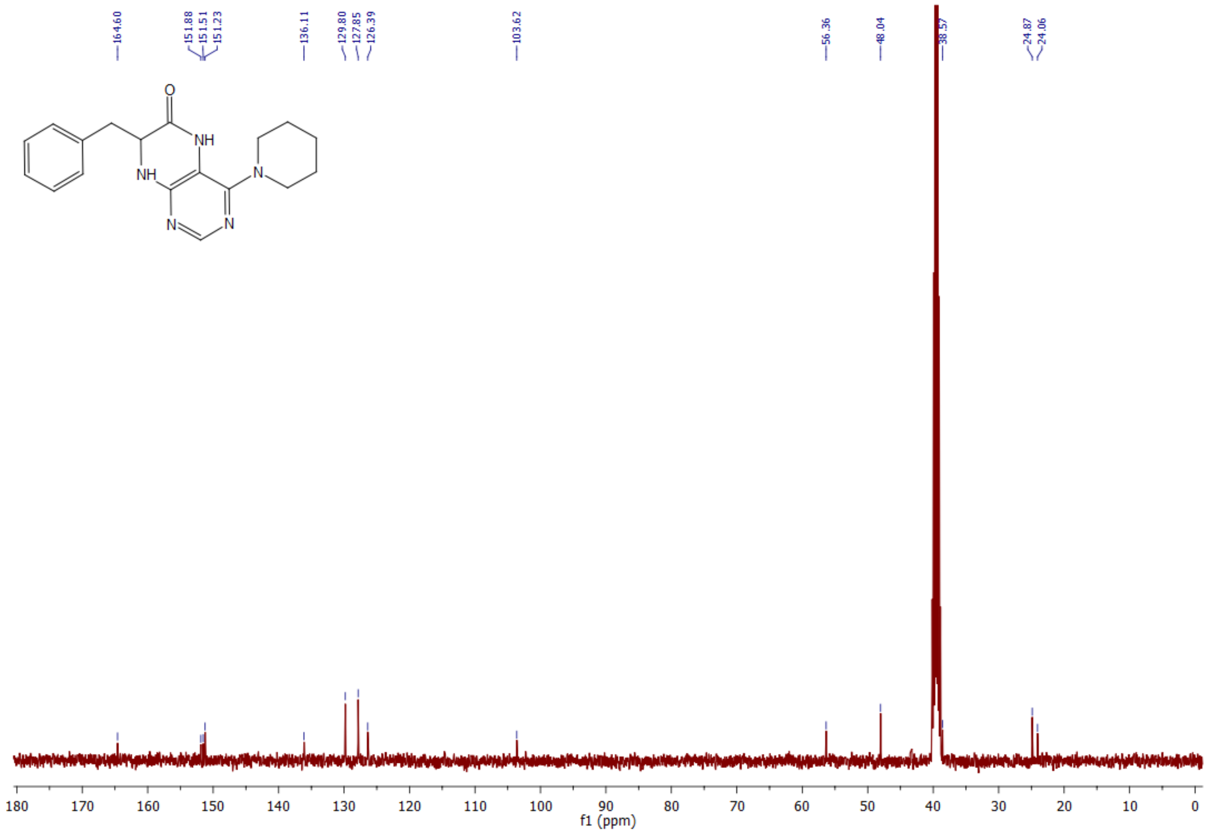

^{1}H and ^{13}C NMR spectra of 7-Benzyl-4-morpholino-7,8-dihydropteridin-6(5H)-one (1p)

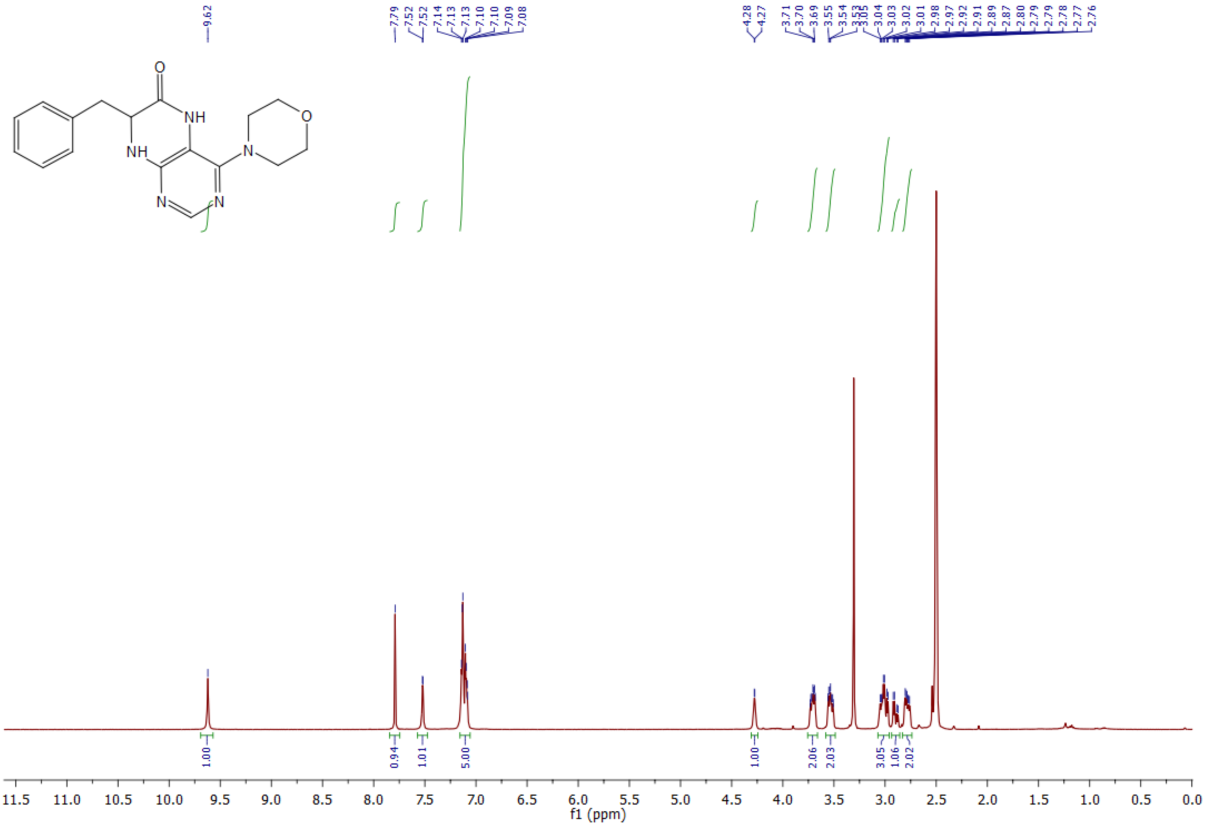

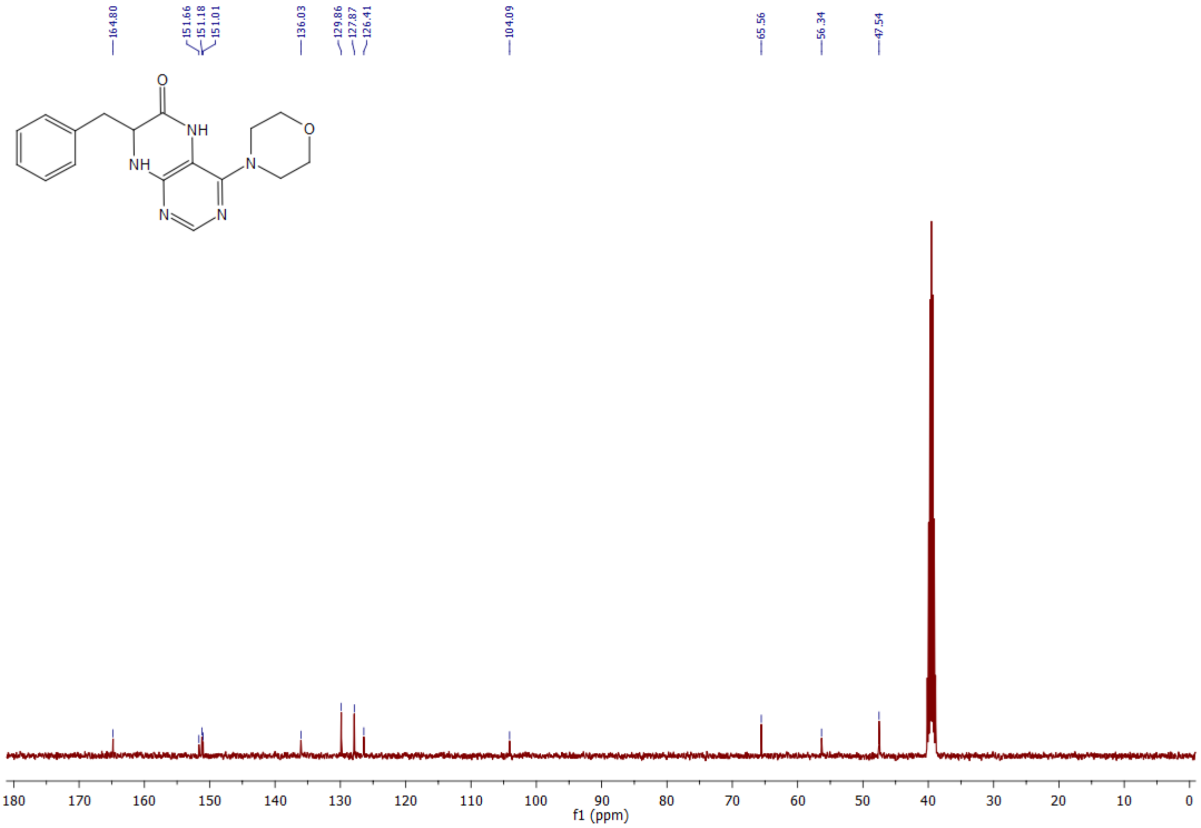

^{1}H and ^{13}C NMR spectra of 7-(2-(Methylthio)ethyl)-4-(propylamino)-7,8-dihydropteridin-6(5H)-one (1q)

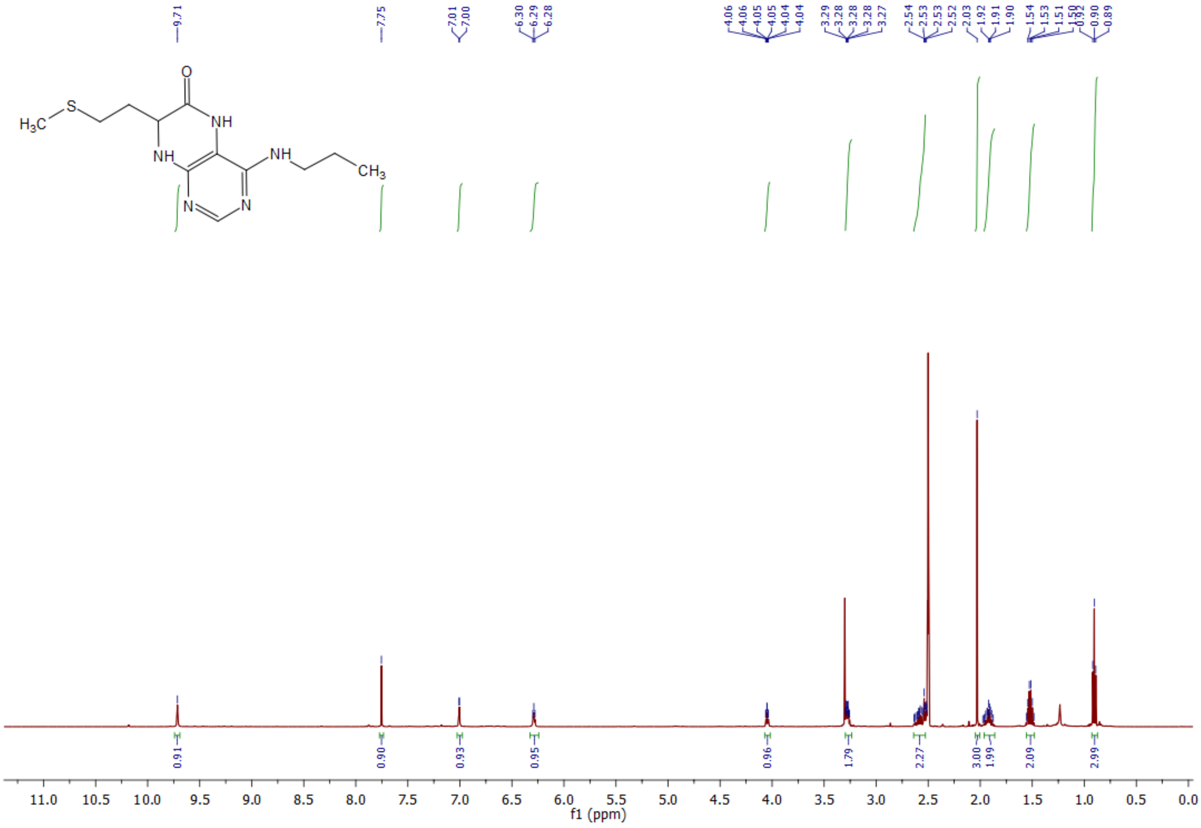

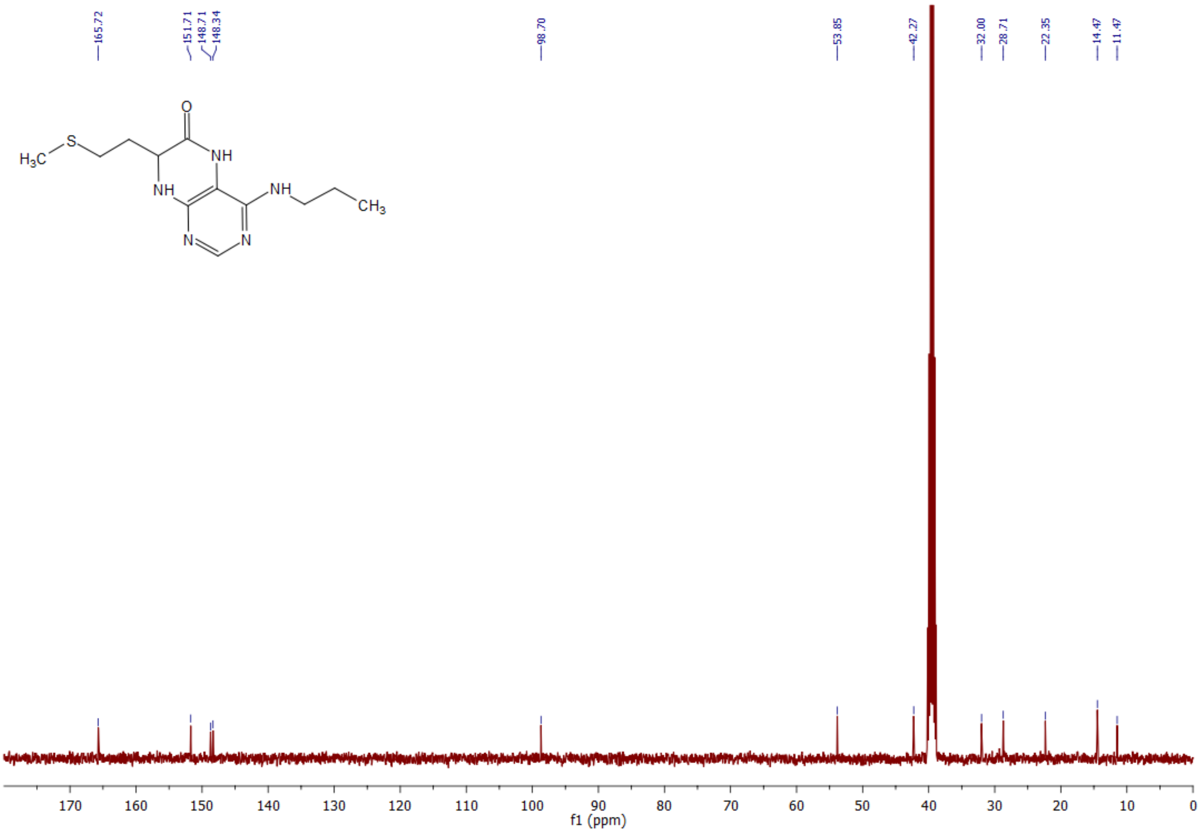

^{1}H and ^{13}C NMR spectra of 4-(Hexylamino)-7-(2-(methylthio)ethyl)-7,8-dihydropteridin-6(5H)-one (1r)

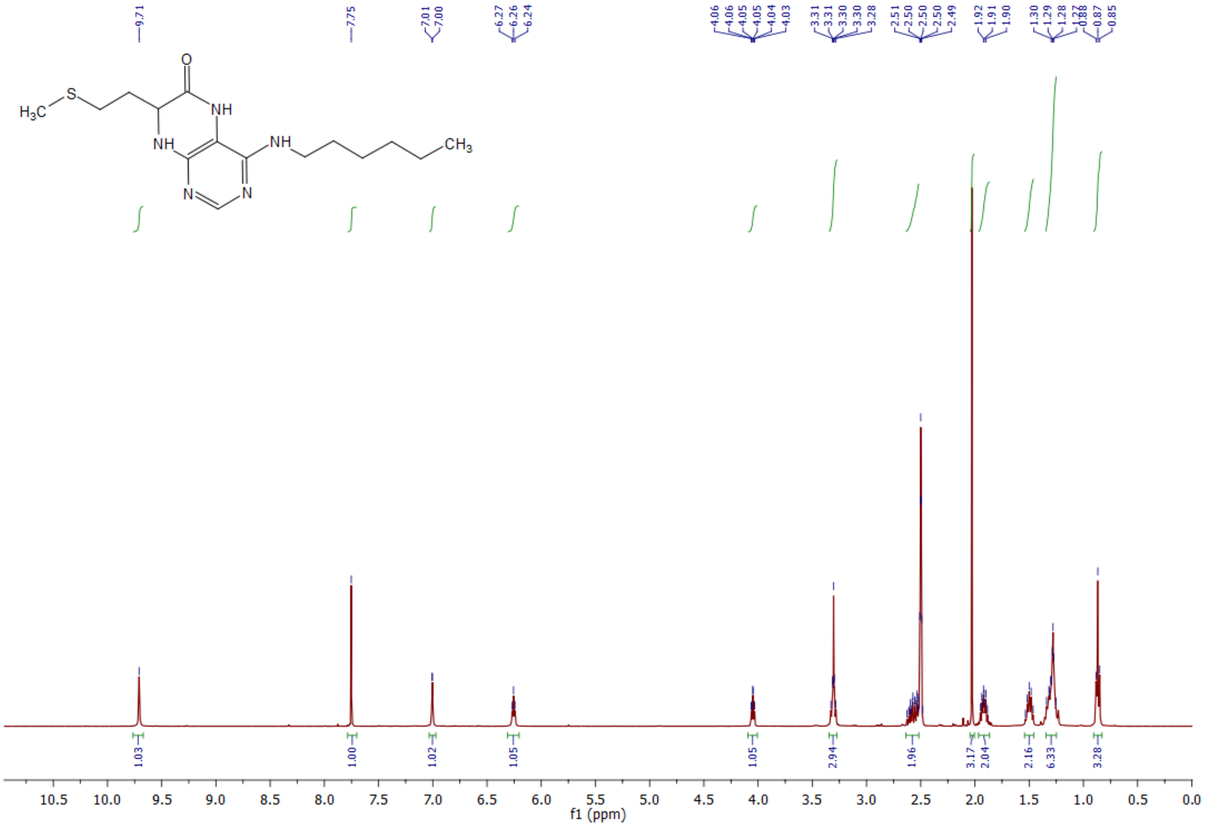

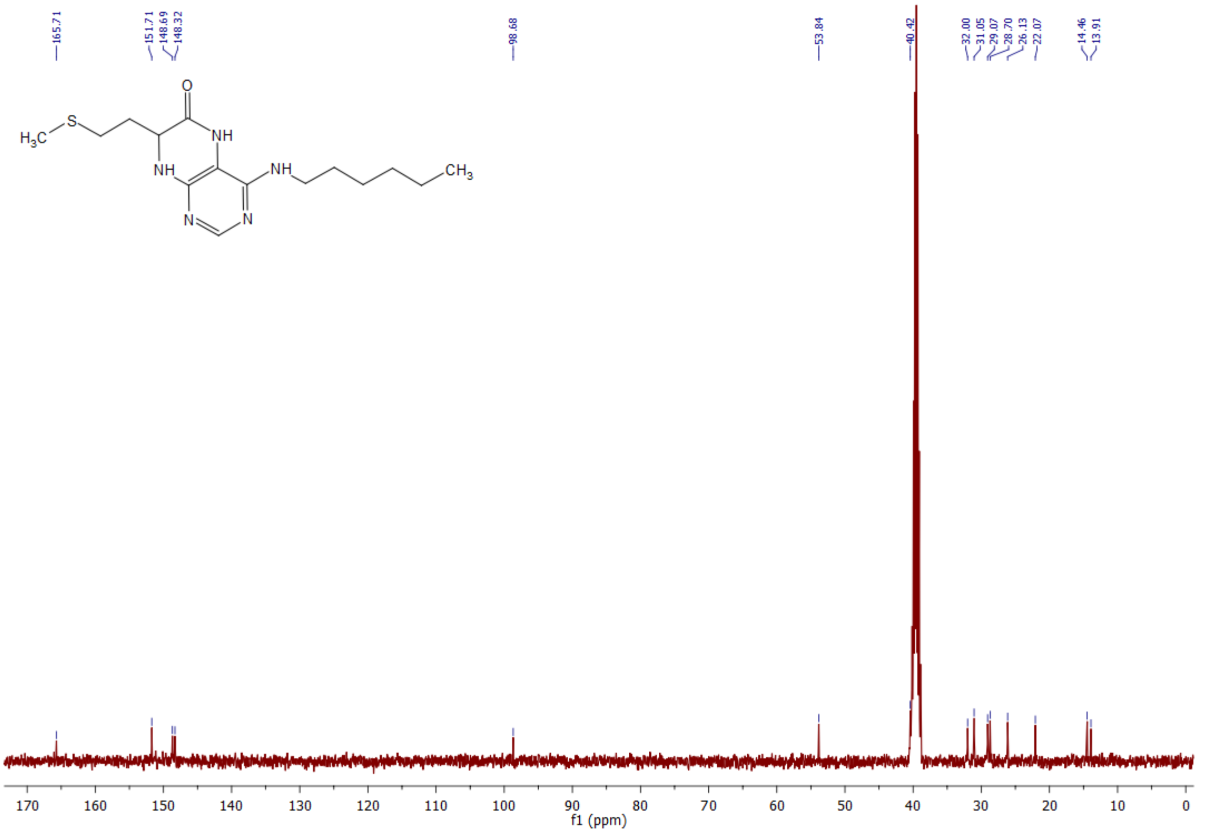

^{1}H and ^{13}C NMR spectra of 4-(Diethylamino)-7-(2-(methylthio)ethyl)-7,8-dihydropteridin-6(5H)-one (1s)

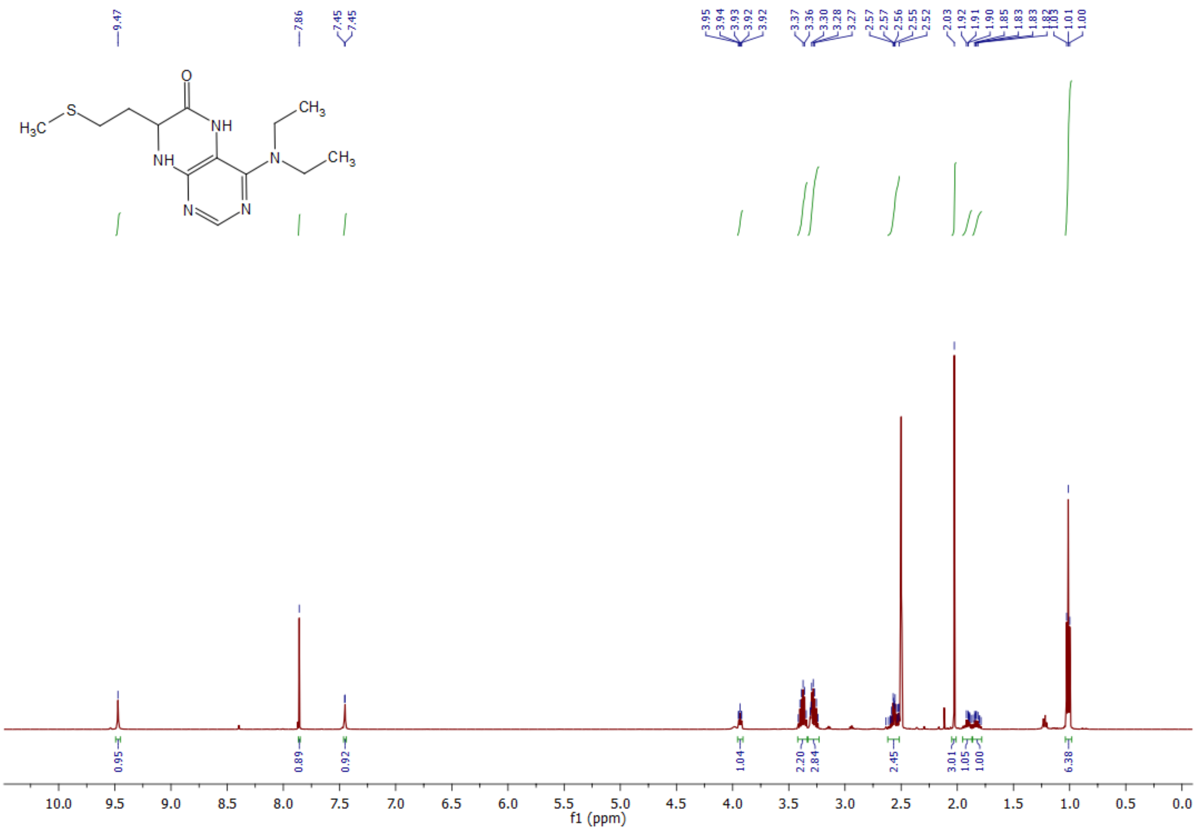

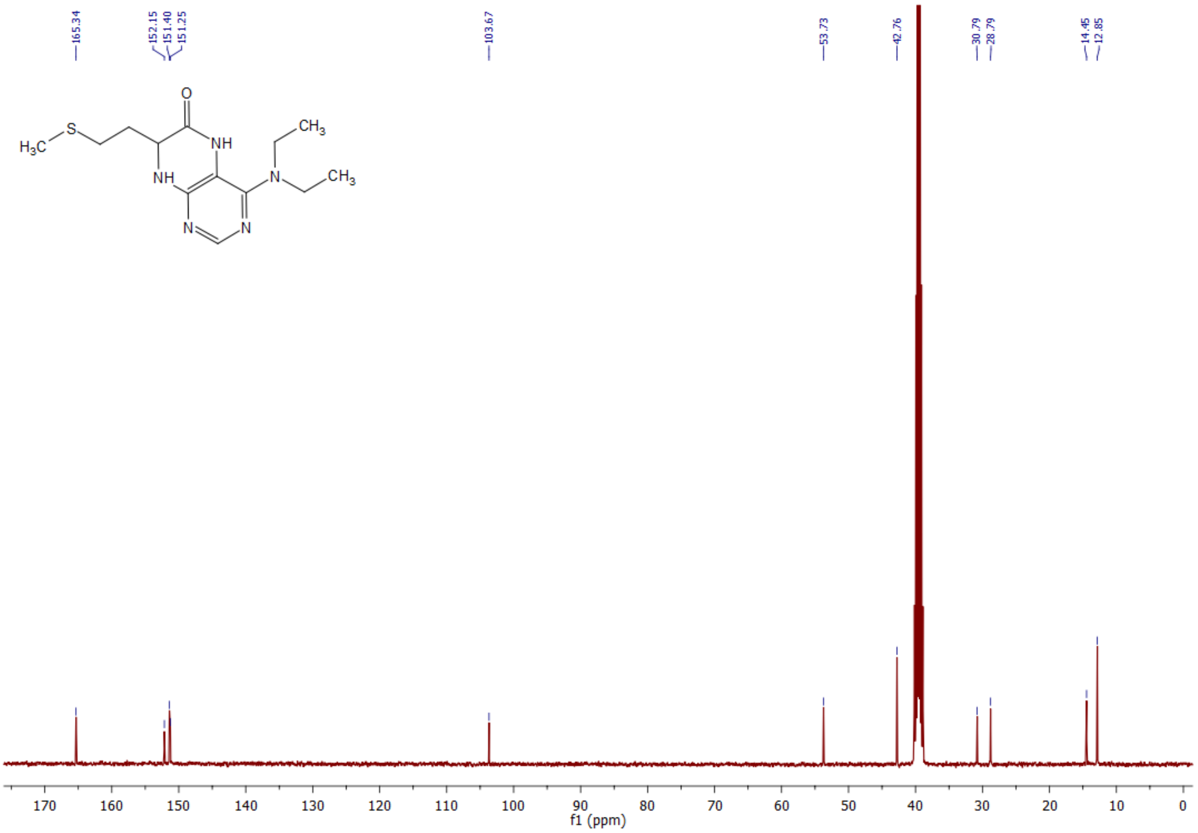

^{1}H and ^{13}C NMR spectra of 4-(Benzylamino)-7-(2-(methylthio)ethyl)-7,8-dihydropteridin-6(5H)-one (1t)

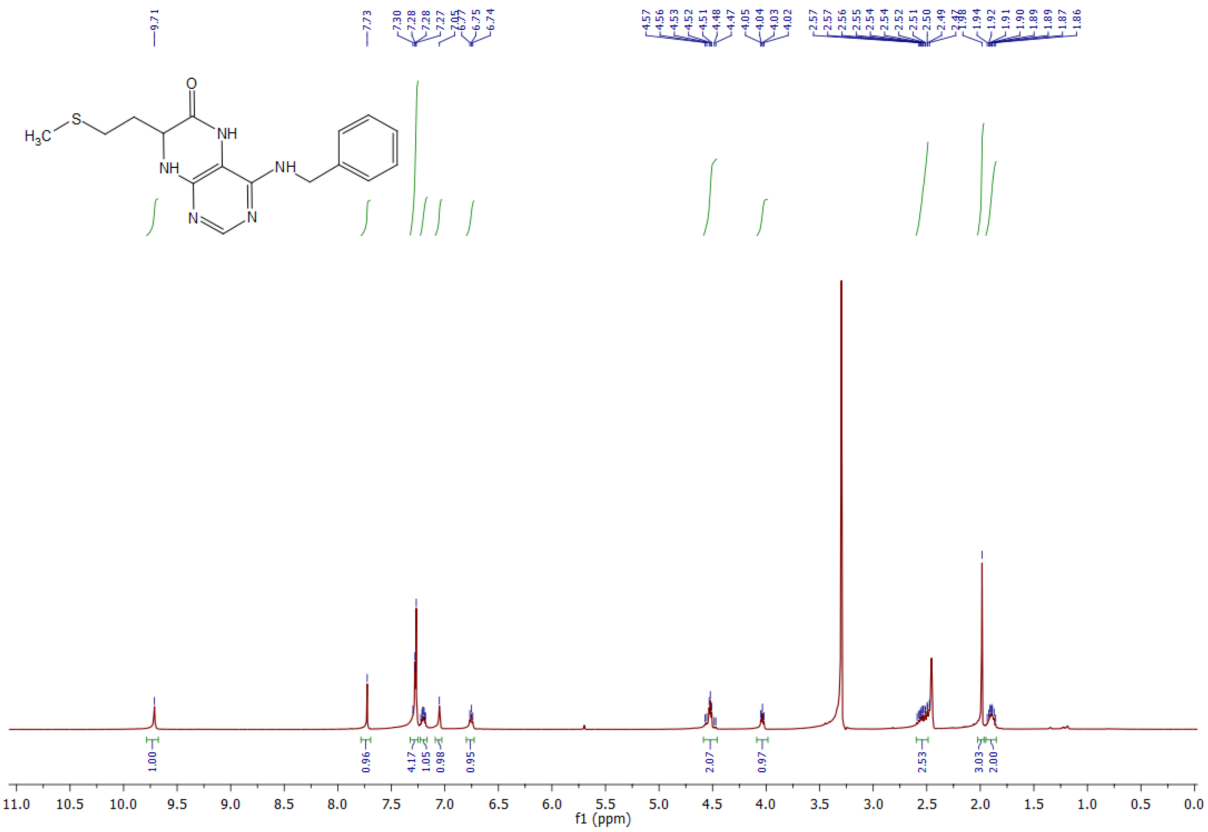

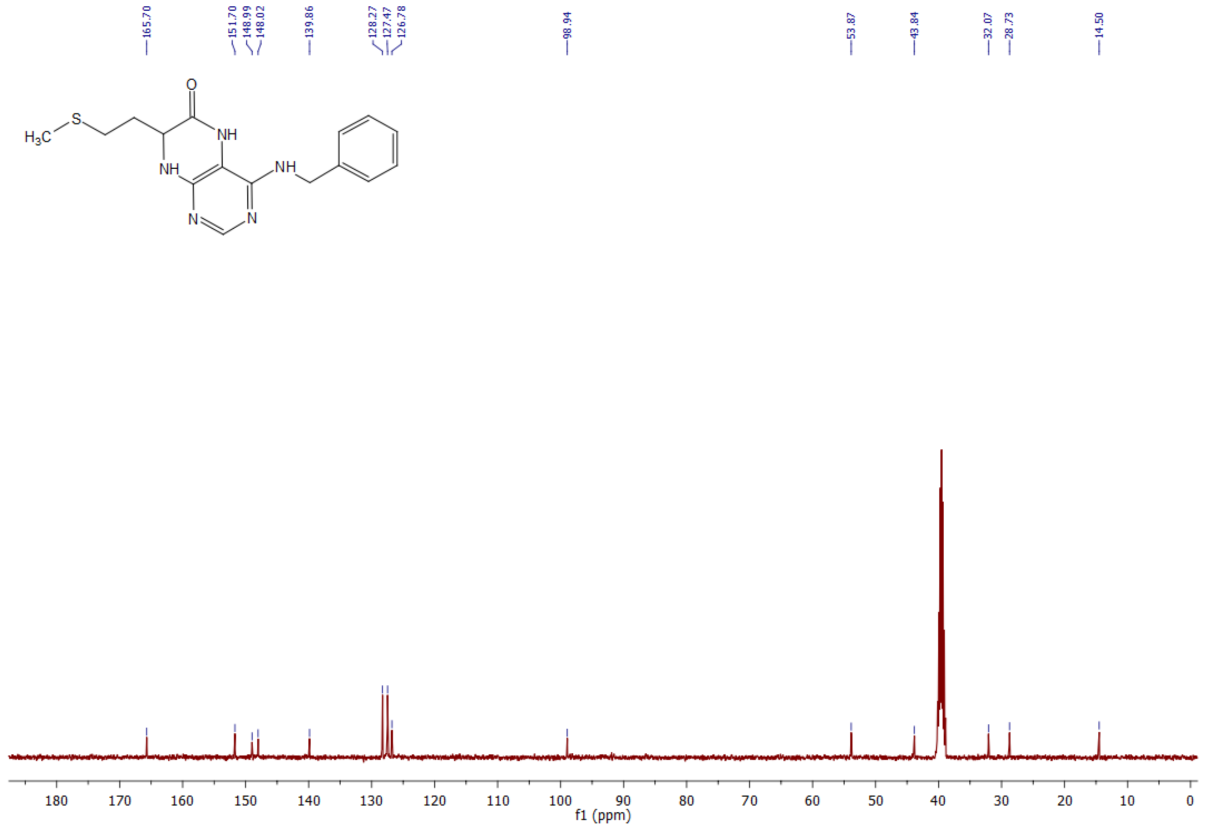

^{1}H and ^{13}C NMR spectra of 4-(Cyclohexylamino)-7-(2-(methylthio)ethyl)-7,8-dihydropteridin-6(5H)-one (1u)

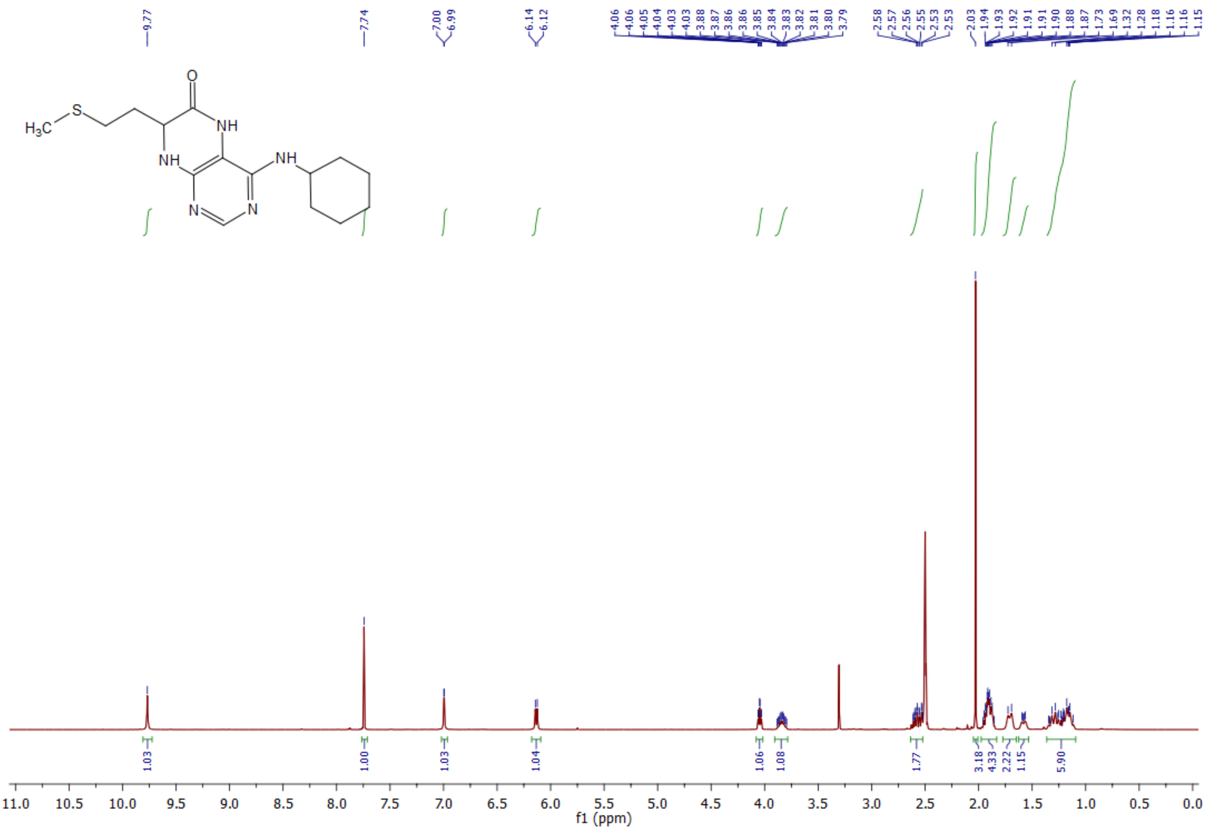

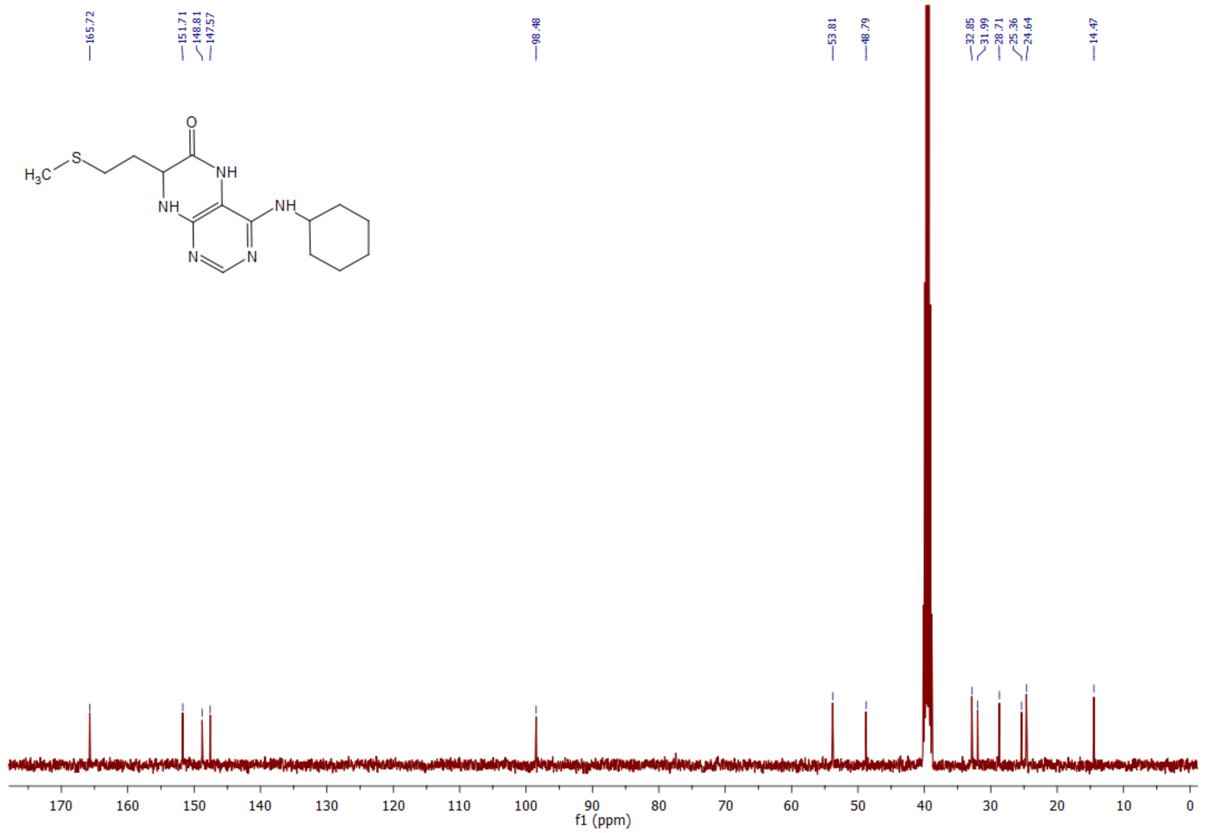

^{1}H and ^{13}C NMR spectra of 4-(Cyclooctylamino)-7-(2-(methylthio)ethyl)-7,8-dihydropteridin-6(5H)-one (1v)

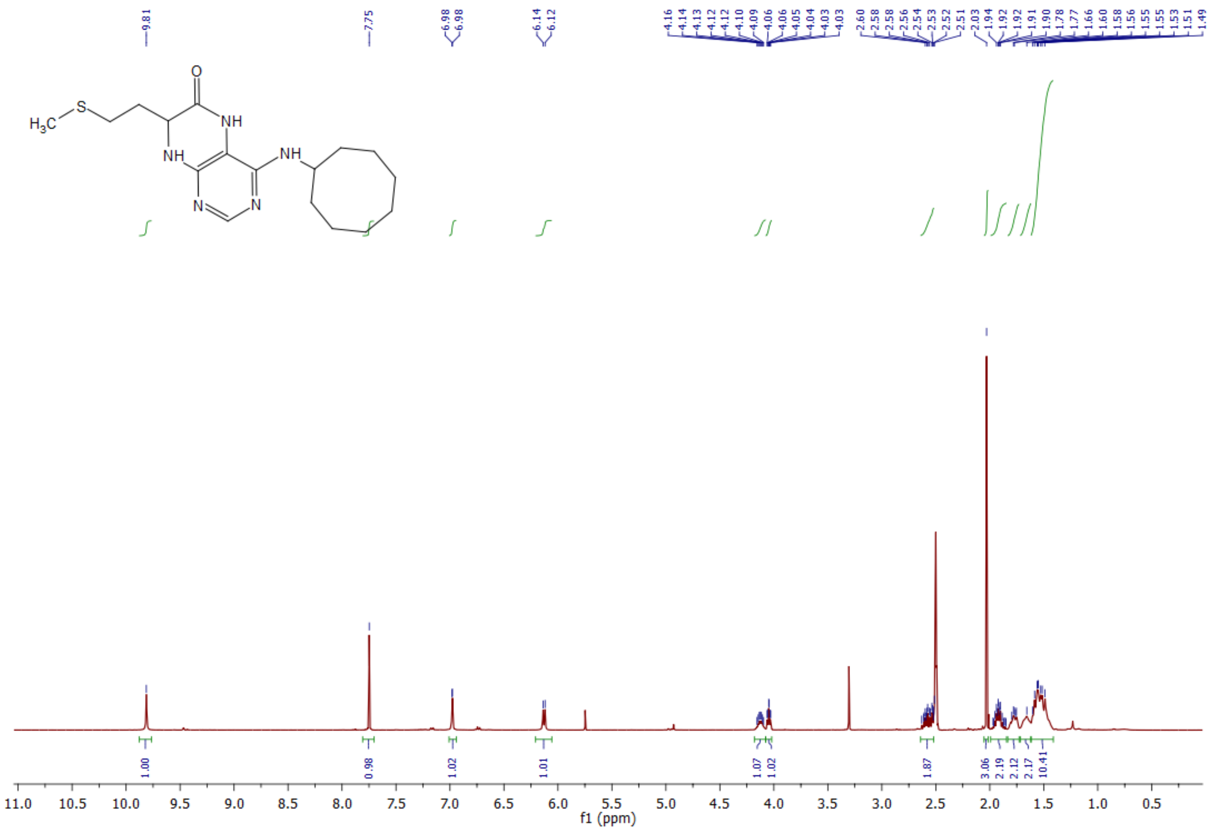

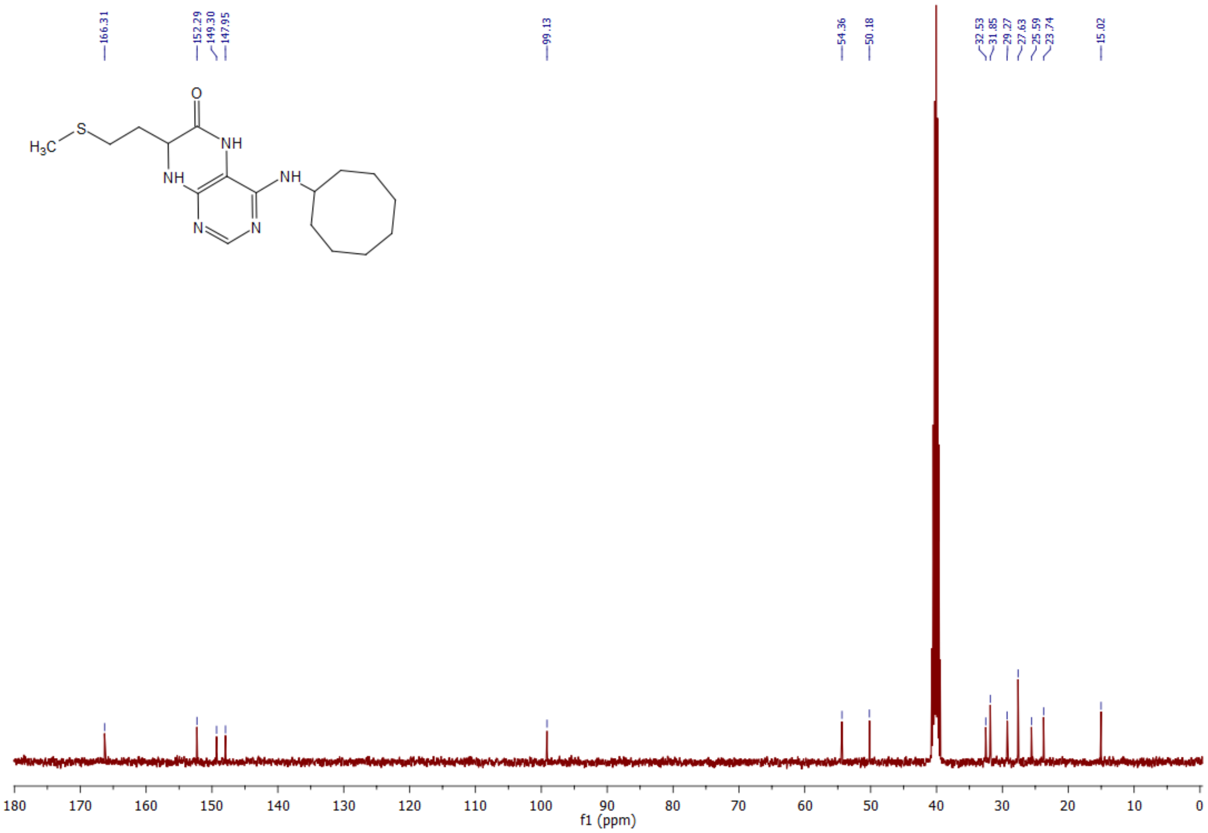

¹H and ¹³C NMR spectra of 7-(2-(Methylthio)ethyl)-4-(piperidin-1-yl)-7,8-dihydropteridin-6(5H)-one (1w)

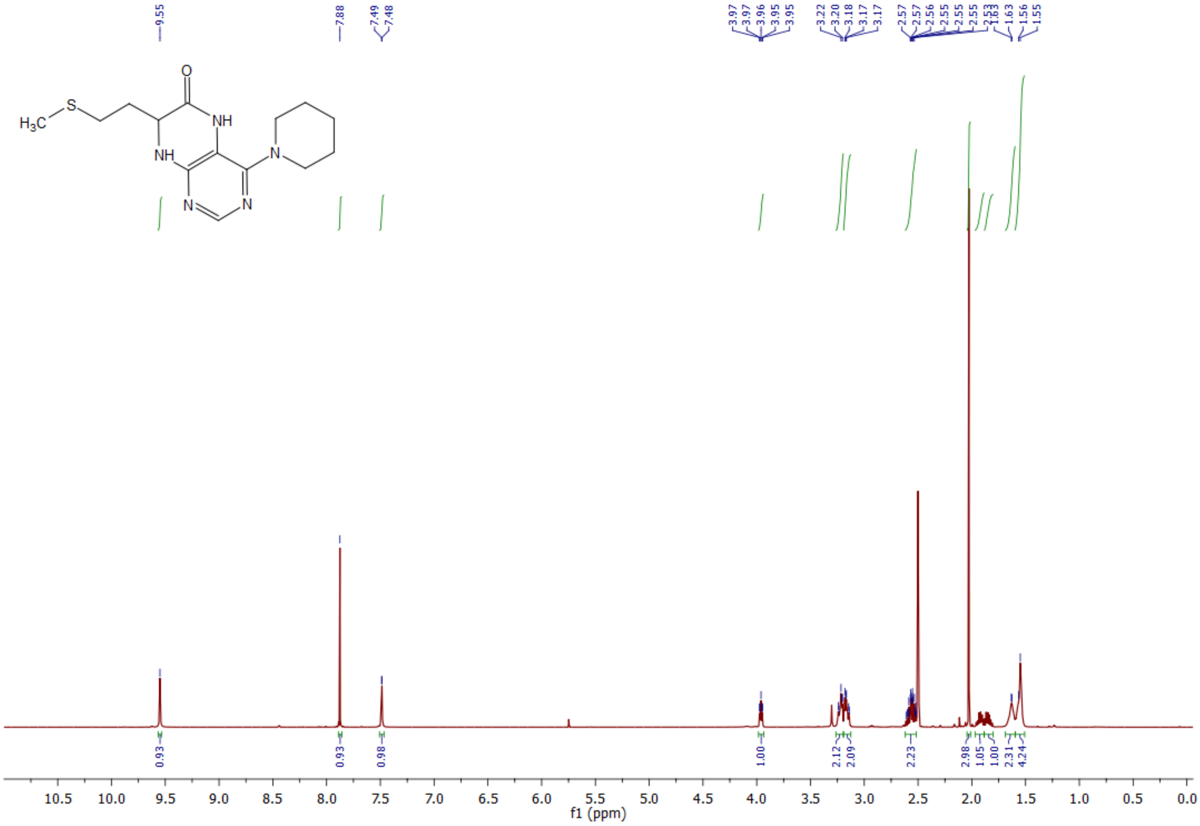

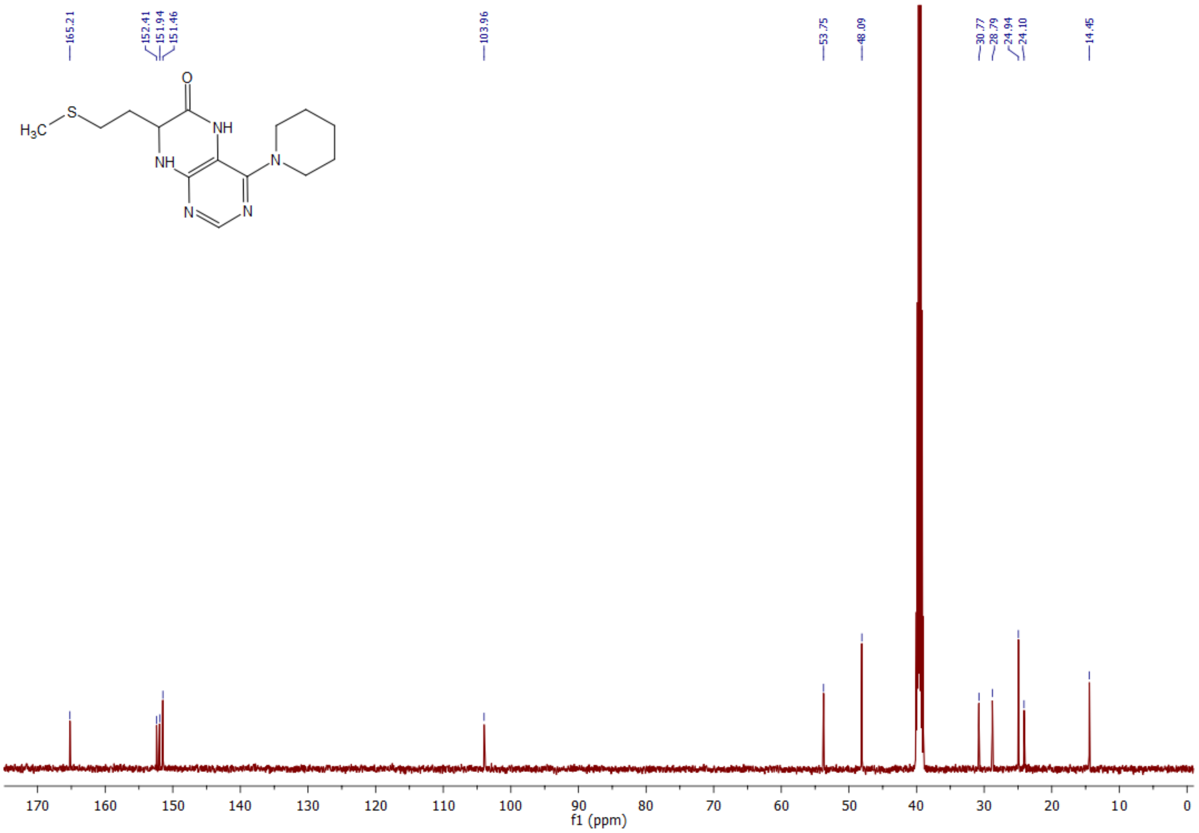

^{1}H and ^{13}C NMR spectra of 7-(2-(Methylthio)ethyl)-4-morpholino-7,8-dihydropteridin-6(5H)-one (1x)

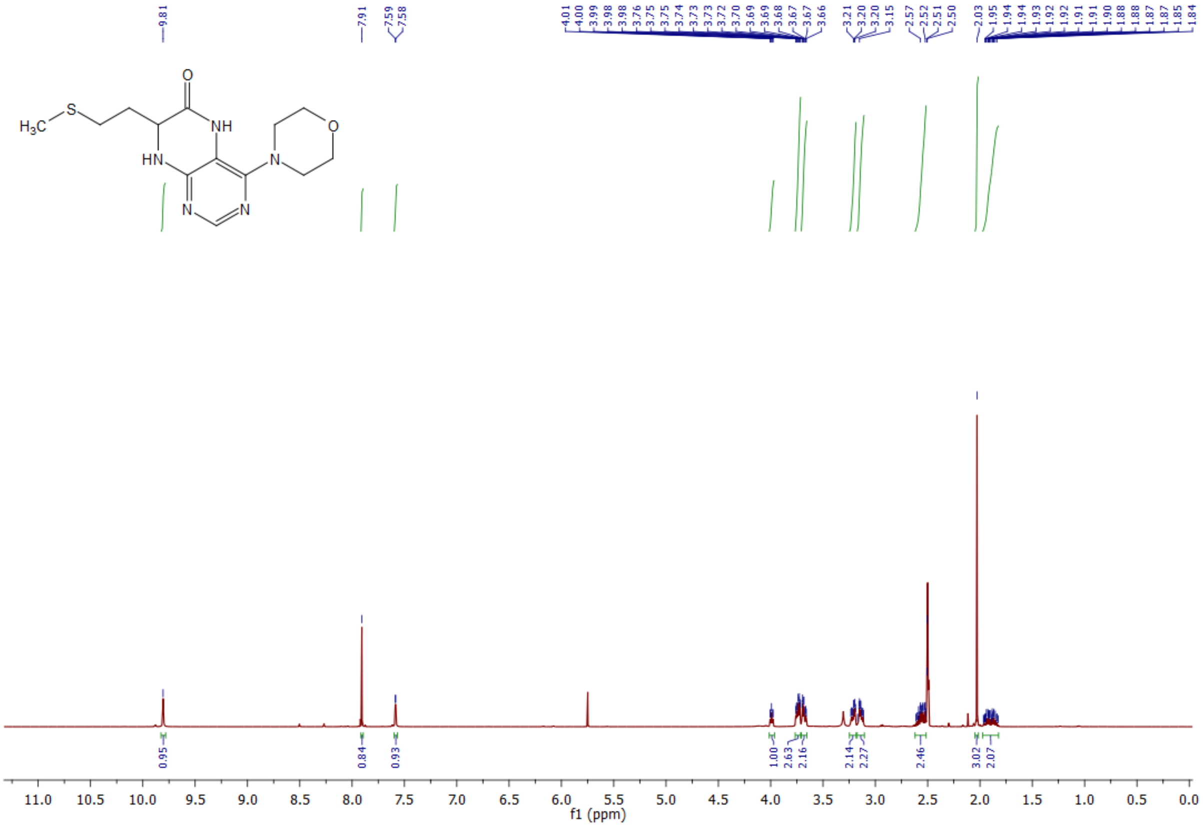

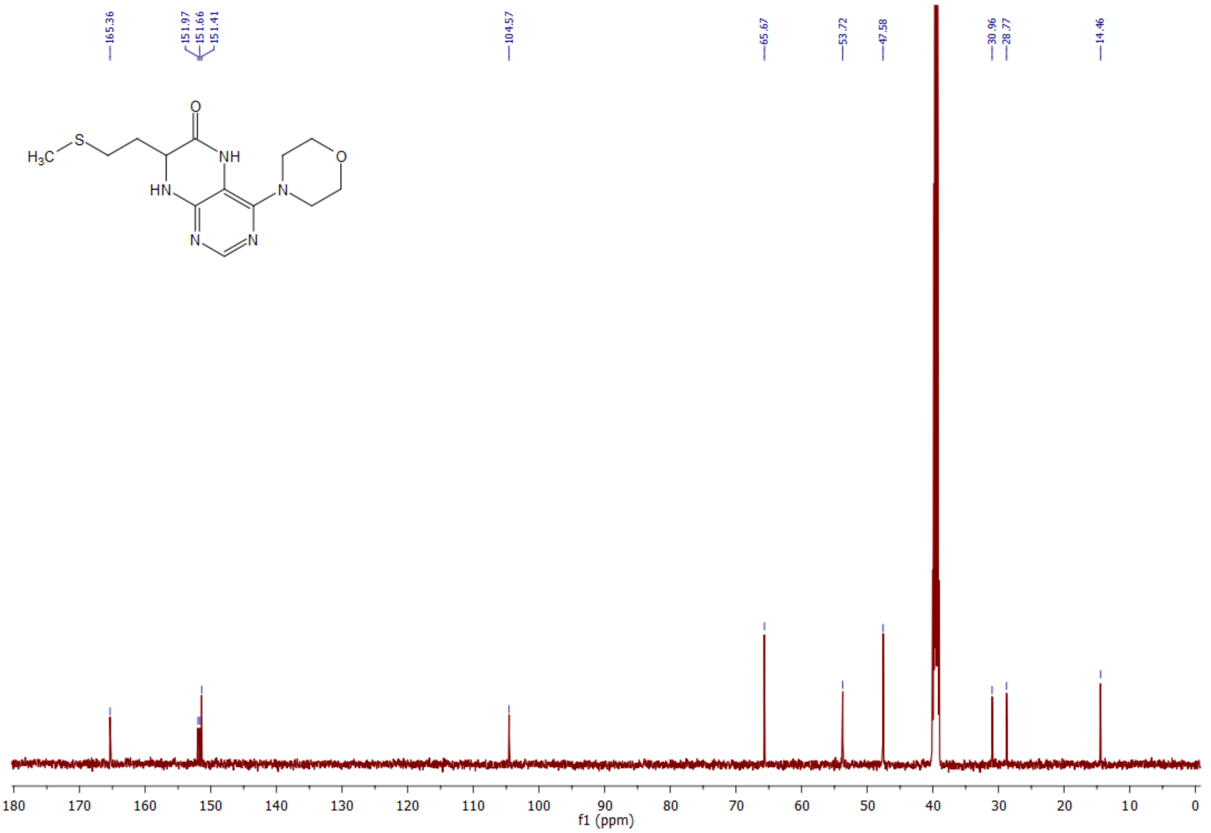

^{1}H and ^{13}C NMR spectra of 4-(Propylamino)-6a,7,8,9-tetrahydropyrrolo[2,1-h]pteridin-6(5H)-one (2a)

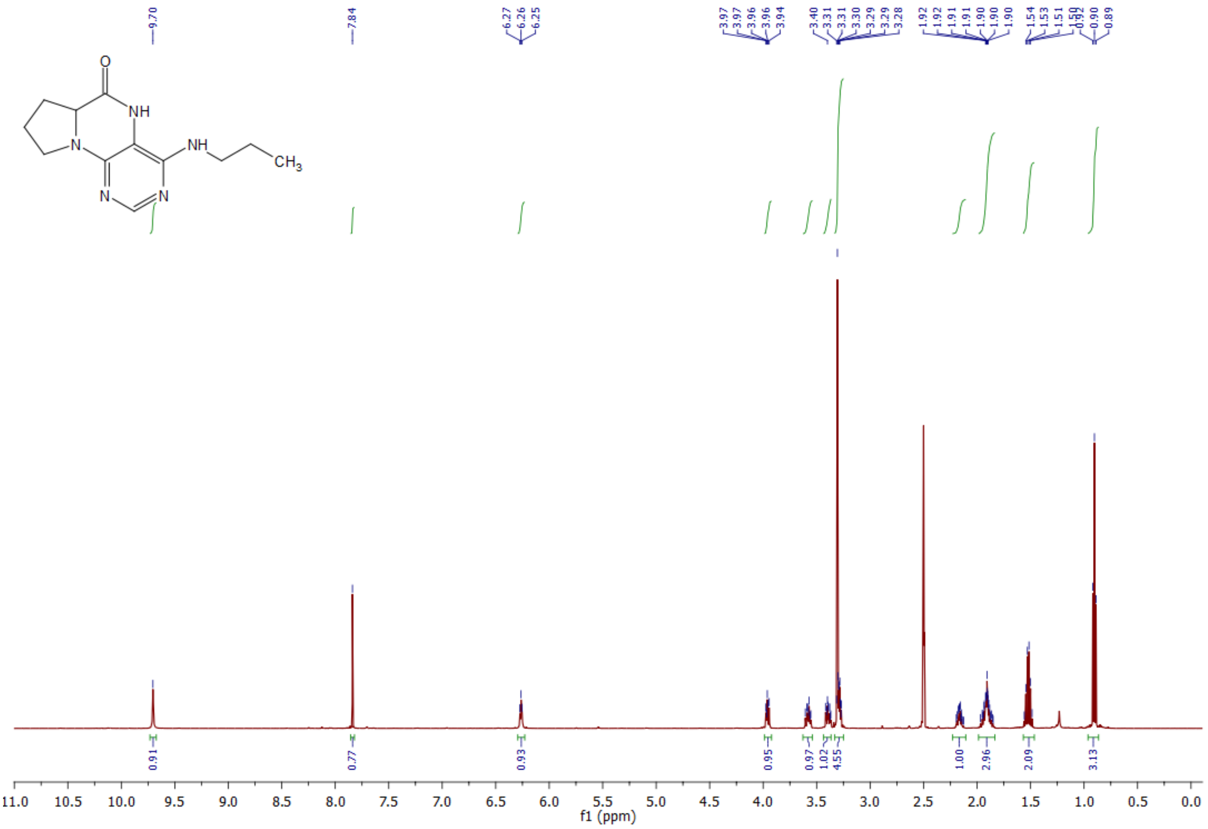

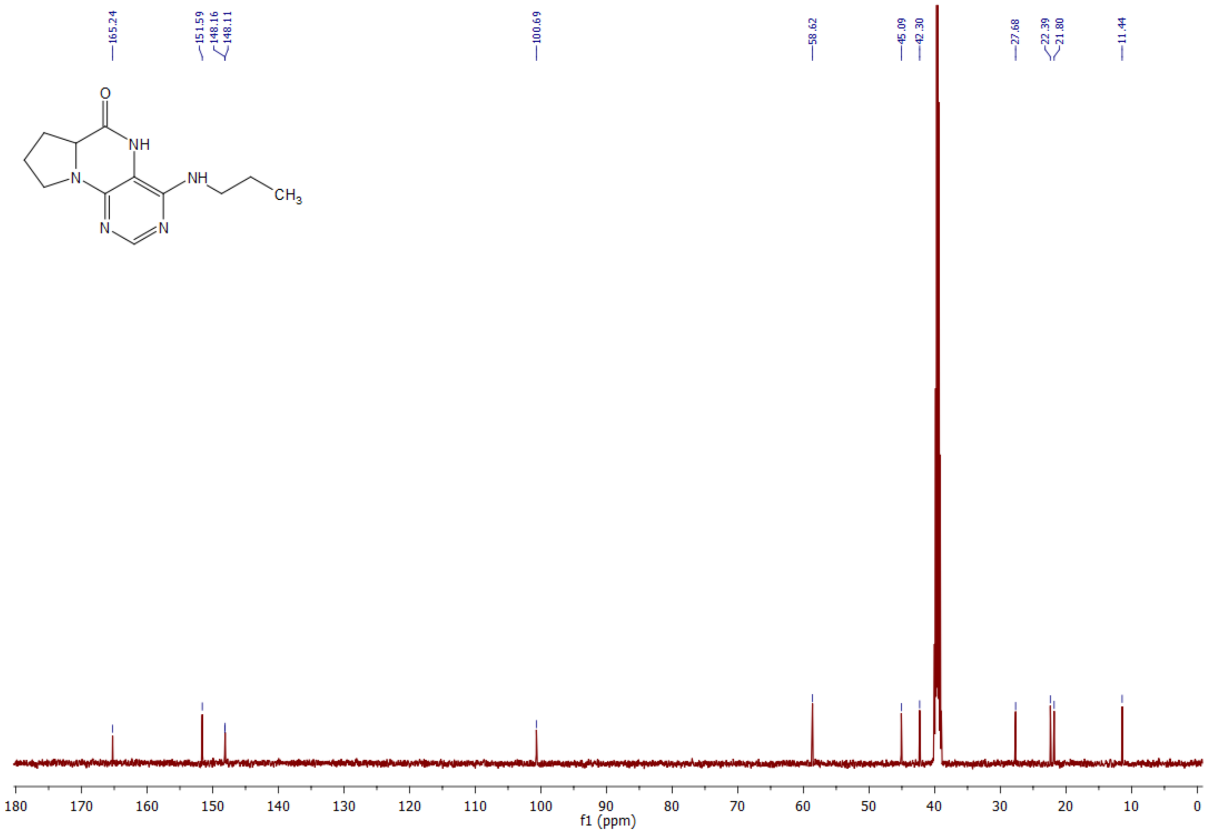

^{1}H and ^{13}C NMR spectra of 4-(Hexylamino)-6a,7,8,9-tetrahydropyrrolo[2,1-h]pteridin-6(5H)-one (2b)

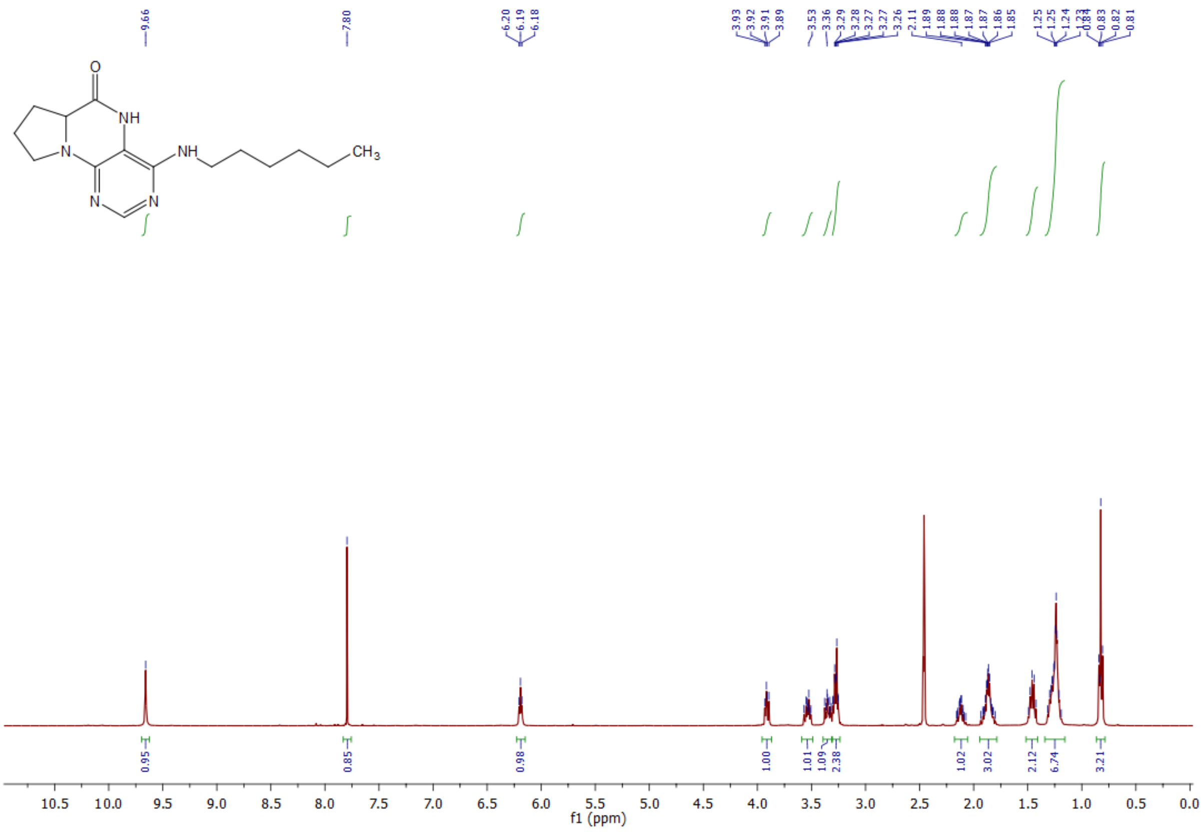

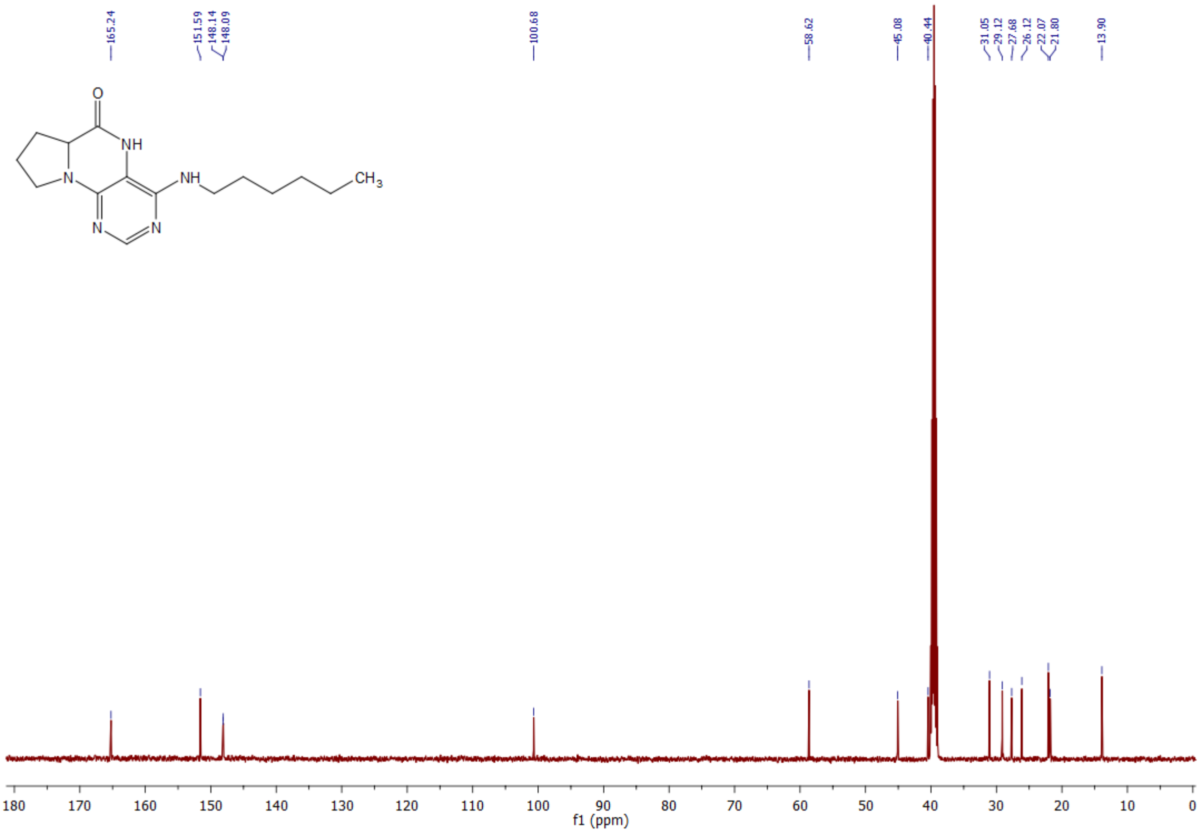

^{1}H and ^{13}C NMR spectra of 4-(Diethylamino)-6a,7,8,9-tetrahydropyrrolo[2,1-h]pteridin-6(5H)-one (2c)

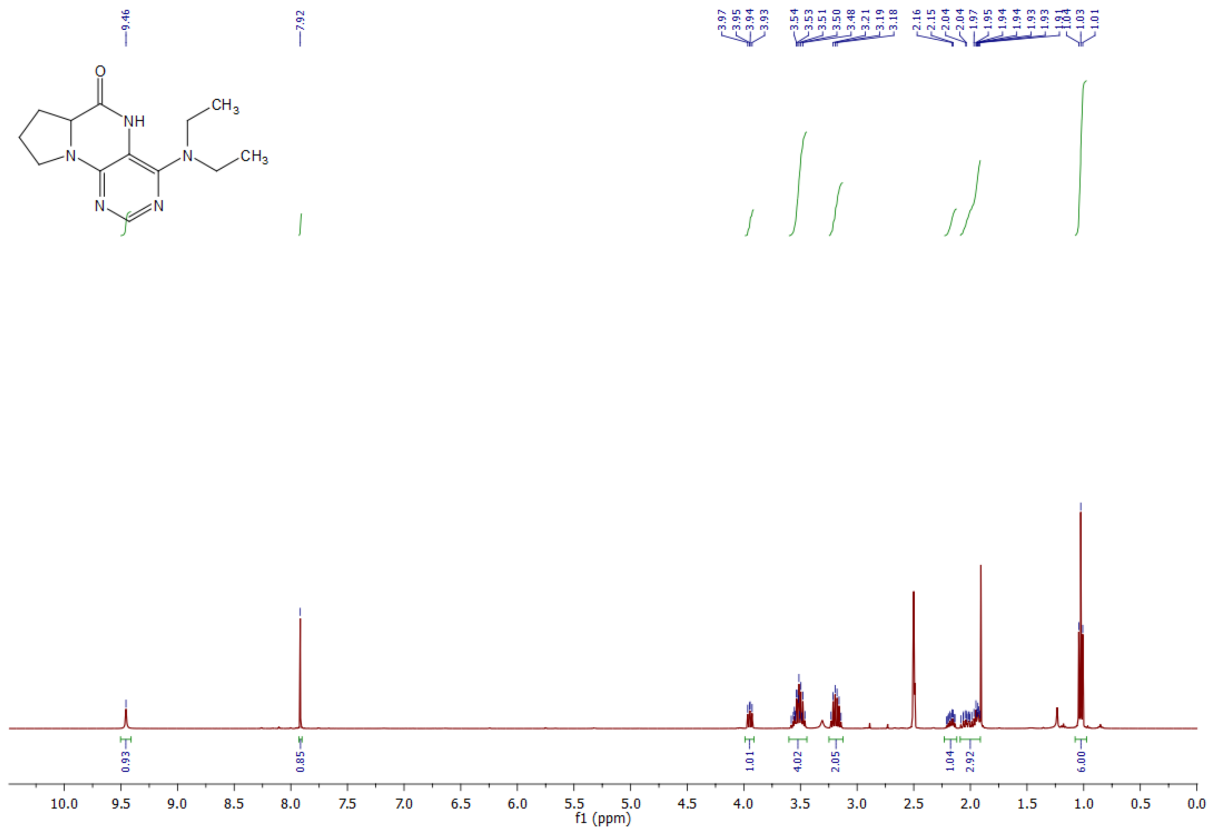

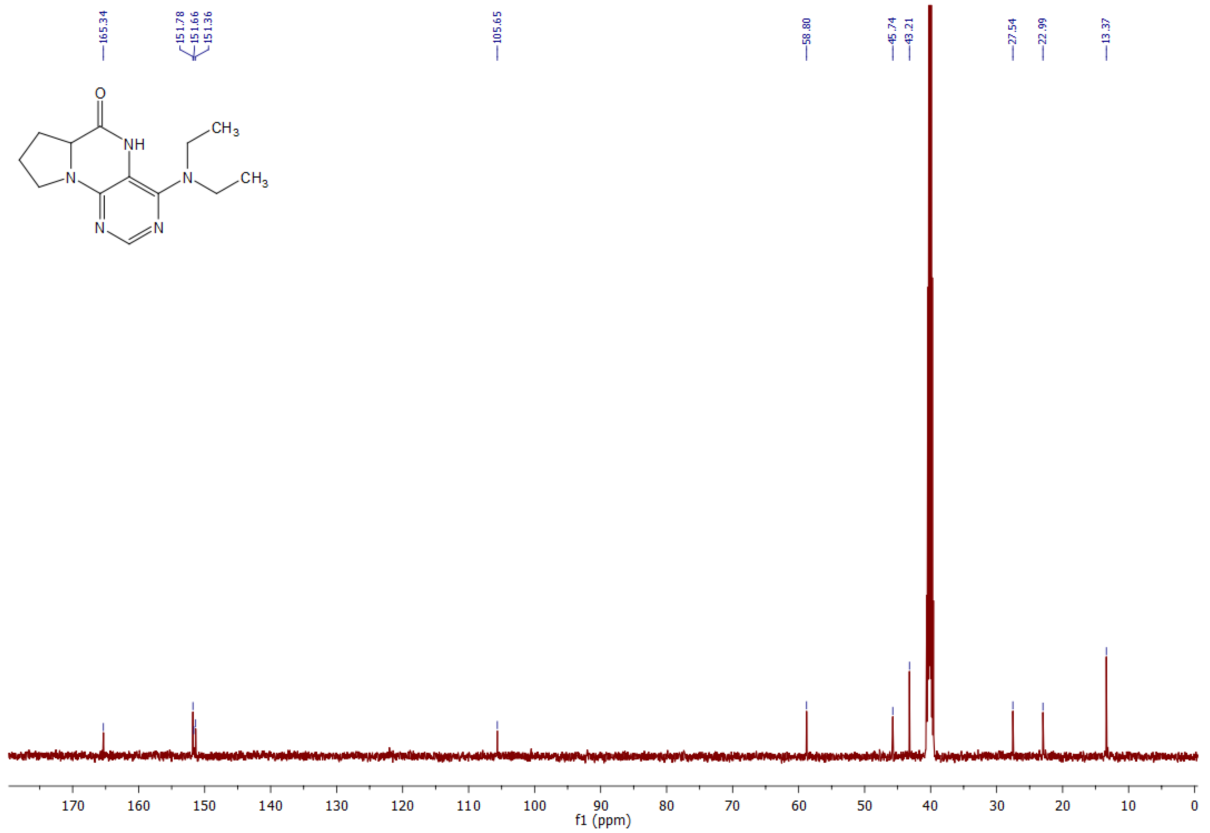

^{1}H and ^{13}C NMR spectra of 4-(Benzylamino)-6a,7,8,9-tetrahydropyrrolo[2,1-h]pteridin-6(5H)-one (2d)

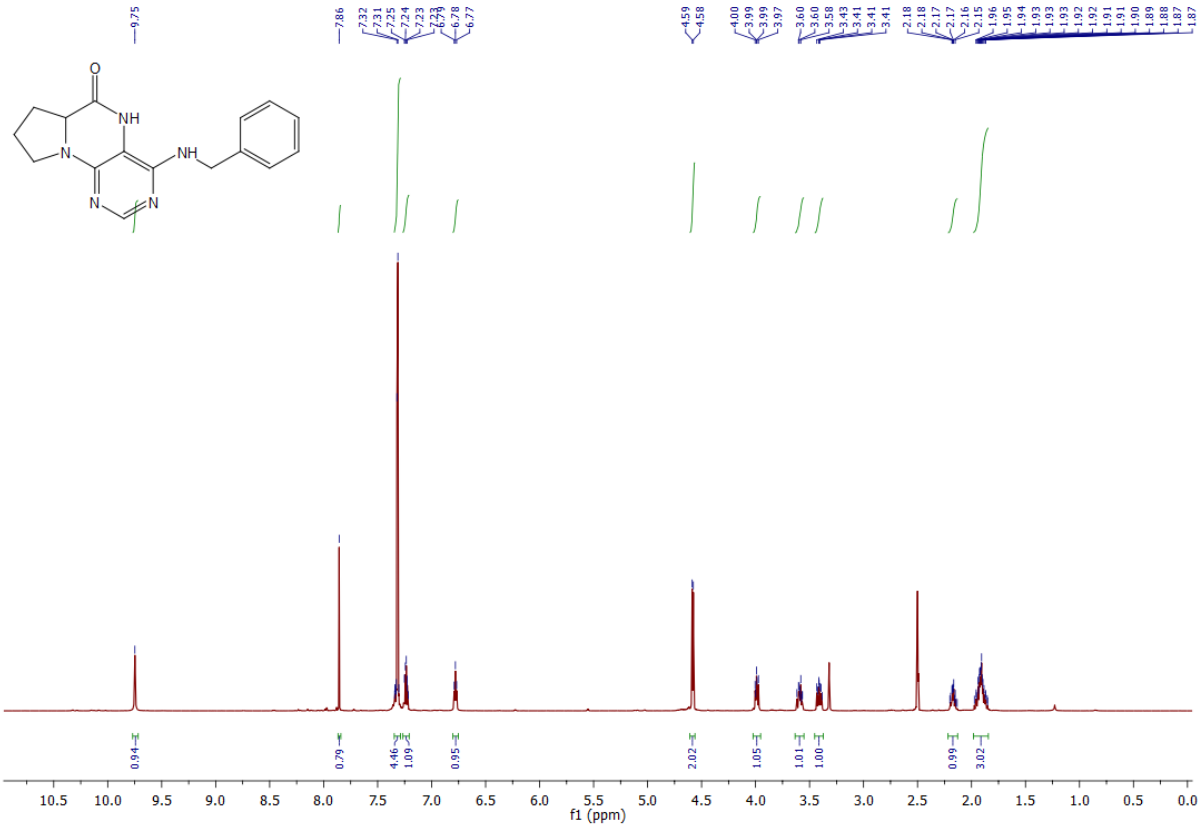

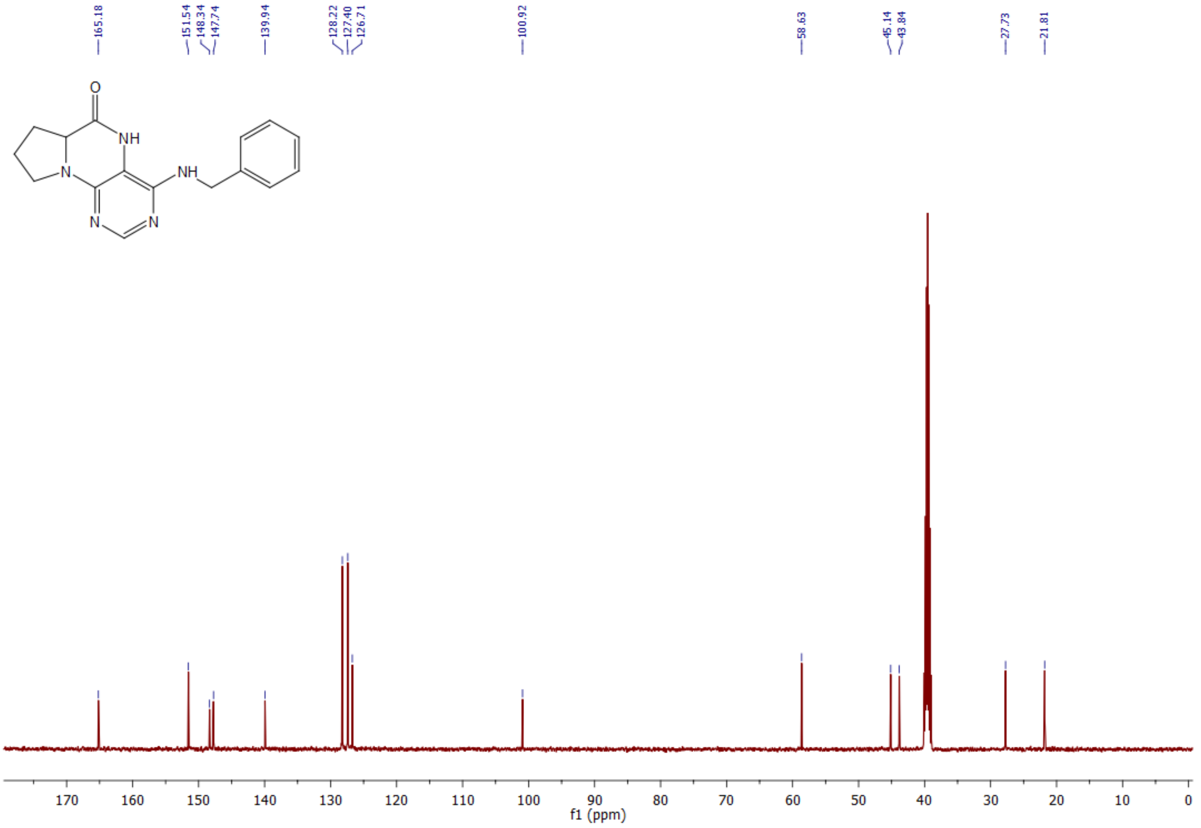

^{1}H and ^{13}C NMR spectra of 4-(Cyclohexylamino)-6a,7,8,9-tetrahydropyrrolo[2,1-h]pteridin-6(5H)-one (2e)

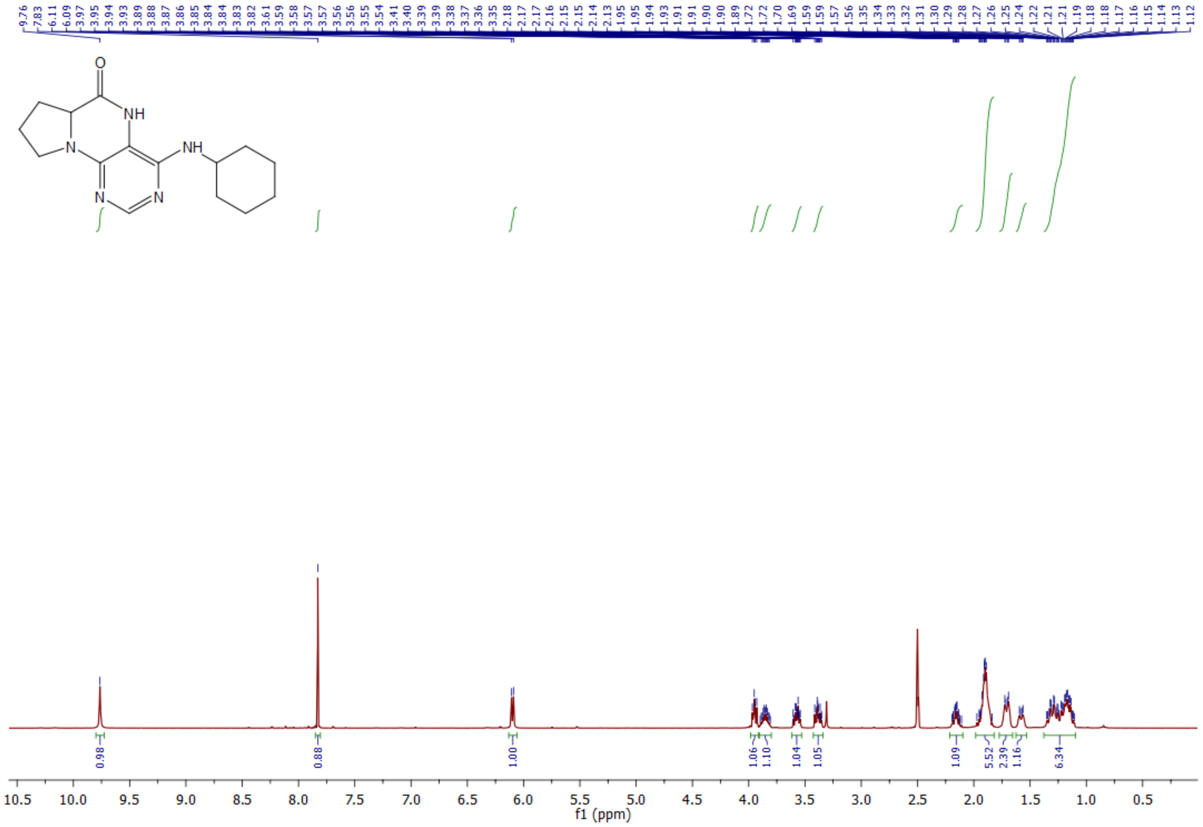

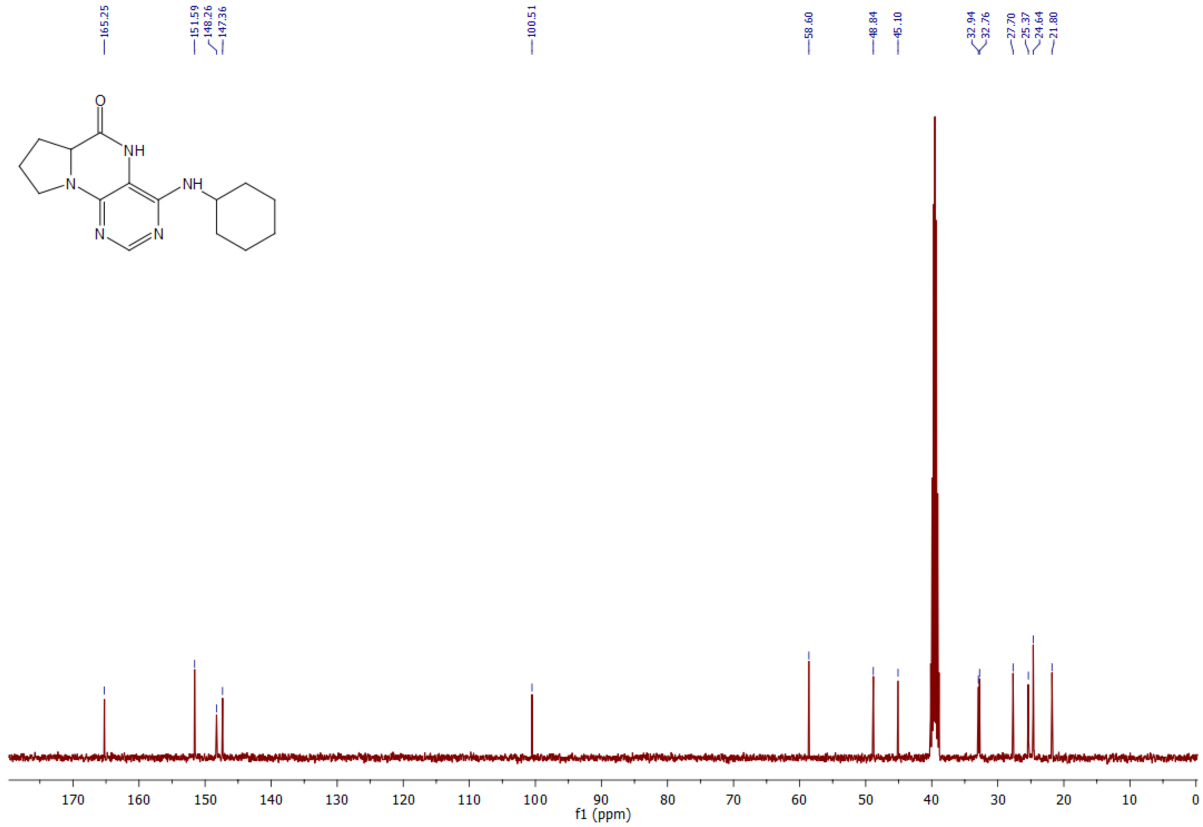

^{1}H and ^{13}C NMR spectra of 4-(Cyclooctylamino)-6a,7,8,9-tetrahydropyrrolo[2,1-h]pteridin-6(5H)-one (2f)

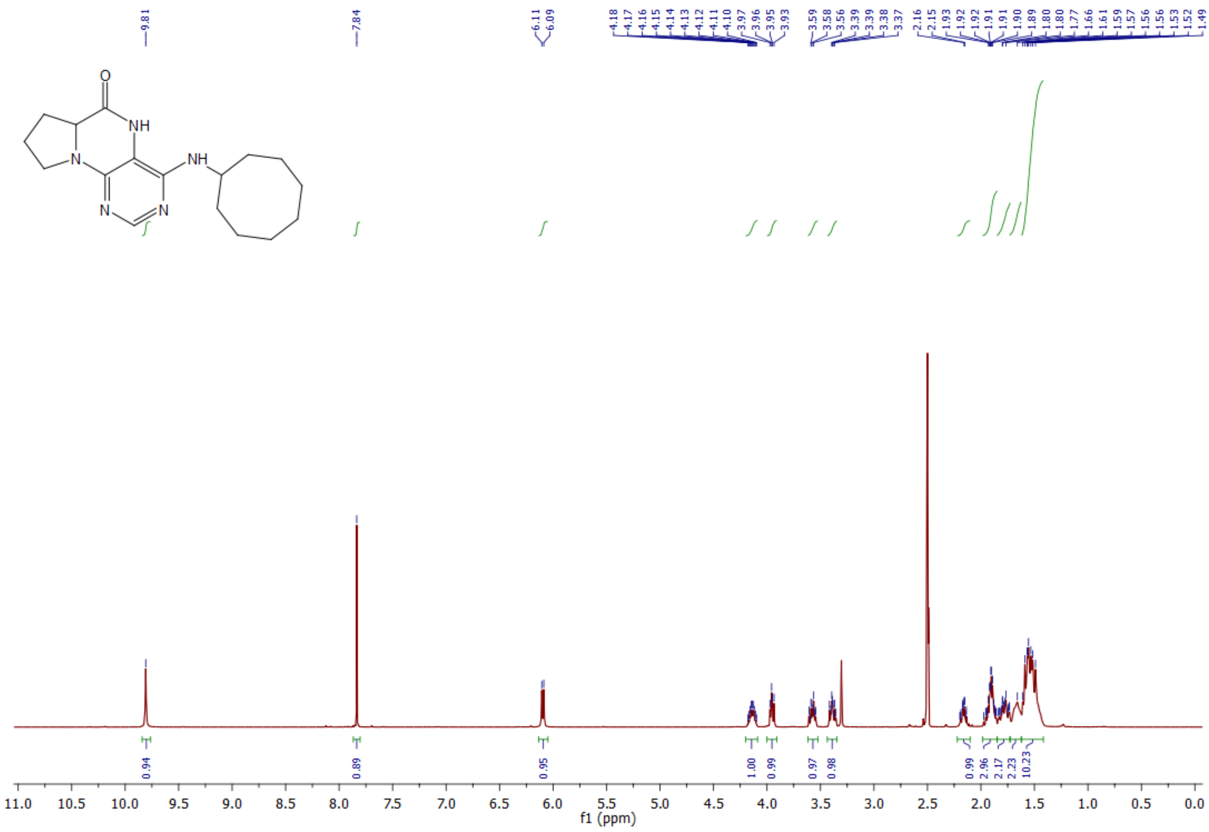

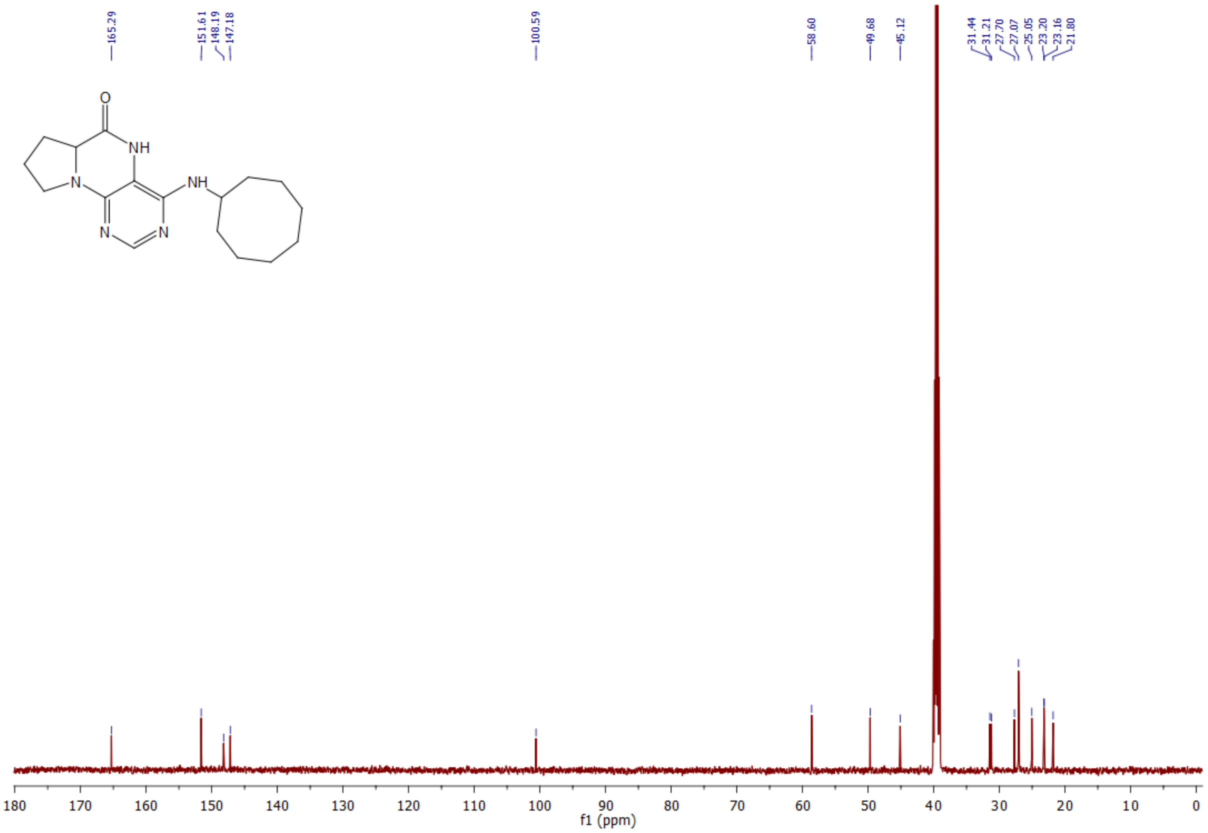

^{1}H and ^{13}C NMR spectra of 4-(Piperidin-1-yl)-6a,7,8,9-tetrahydropyrrolo[2,1-h]pteridin-6(5H)-one (2g)

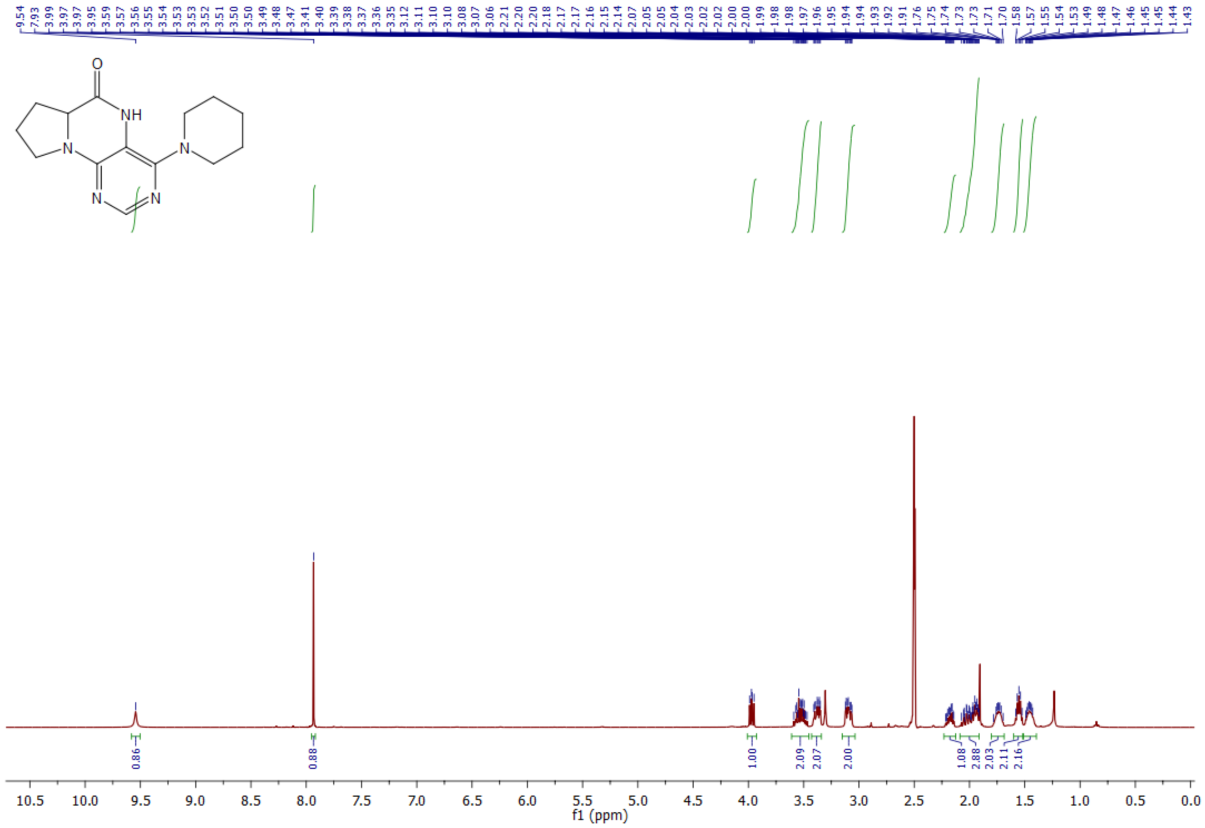

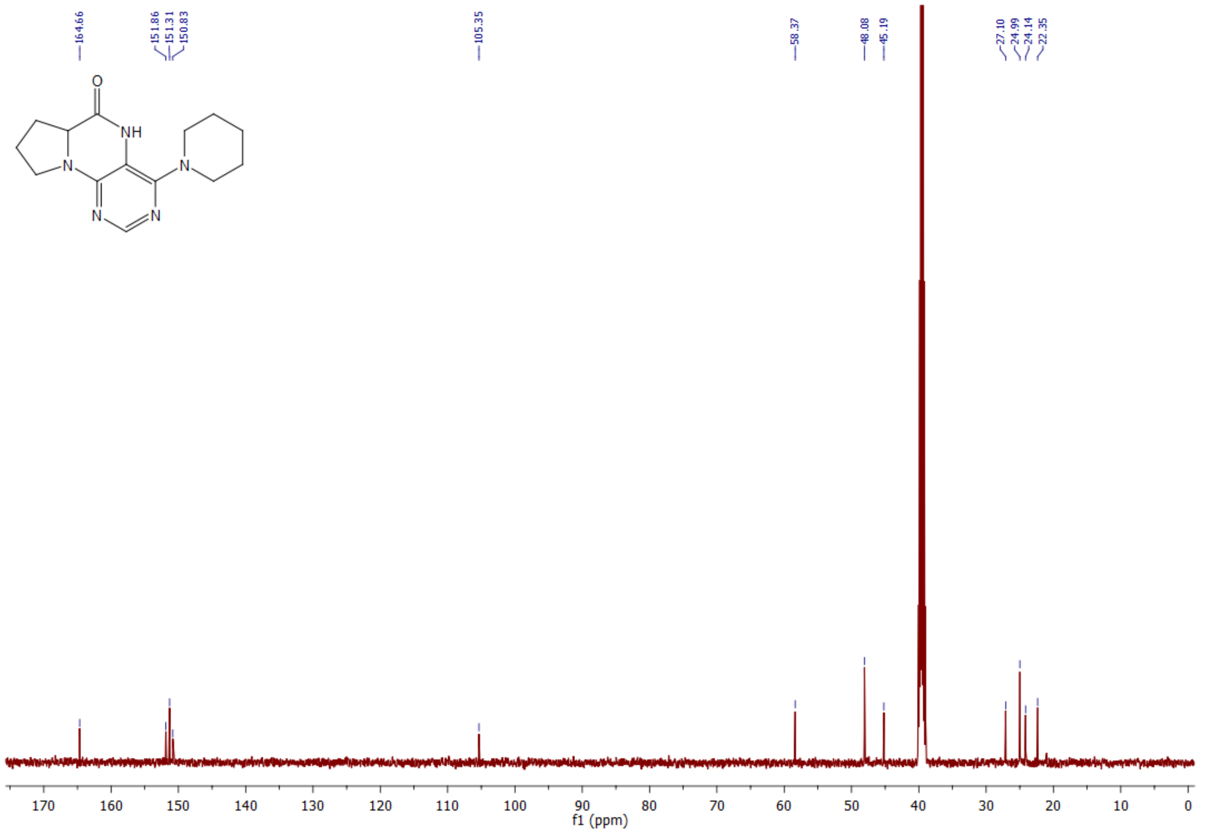

^{1}H and ^{13}C NMR spectra of 4-Morpholino-6a,7,8,9-tetrahydropyrrolo[2,1-h]pteridin-6(5H)-one (2h)

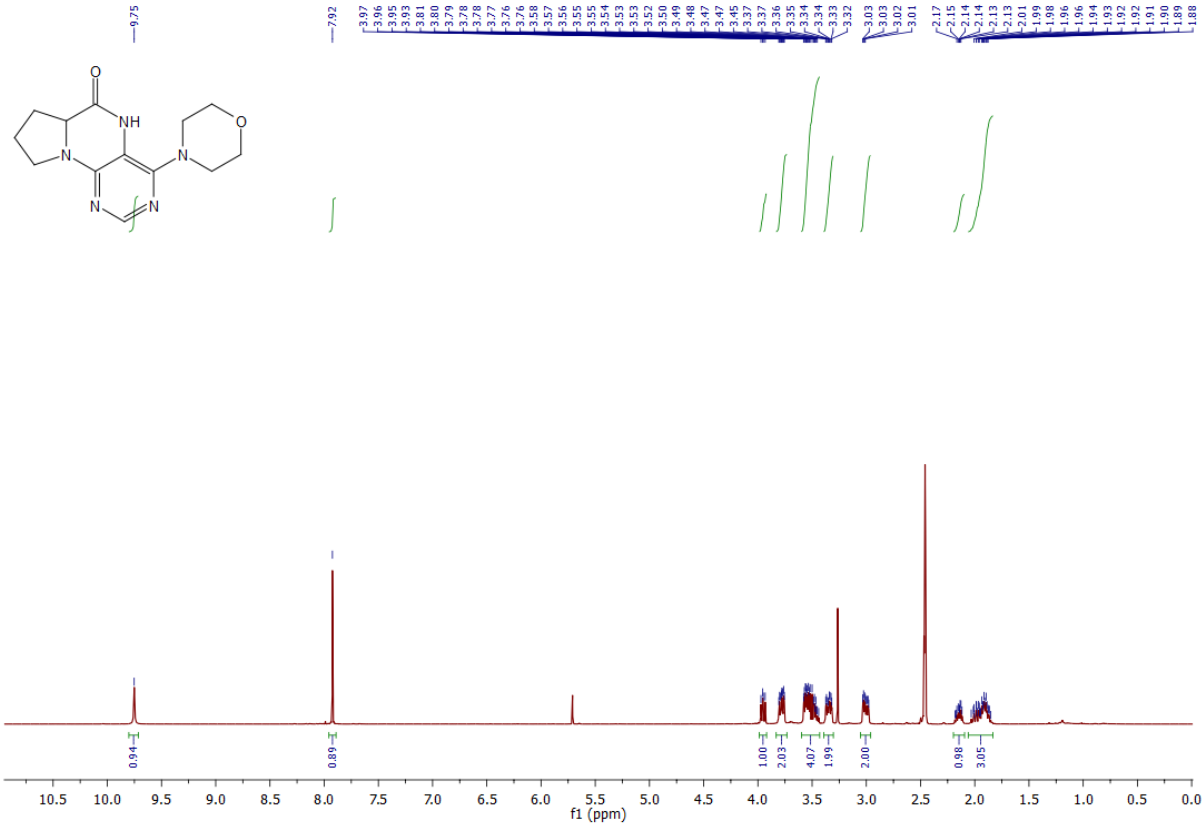

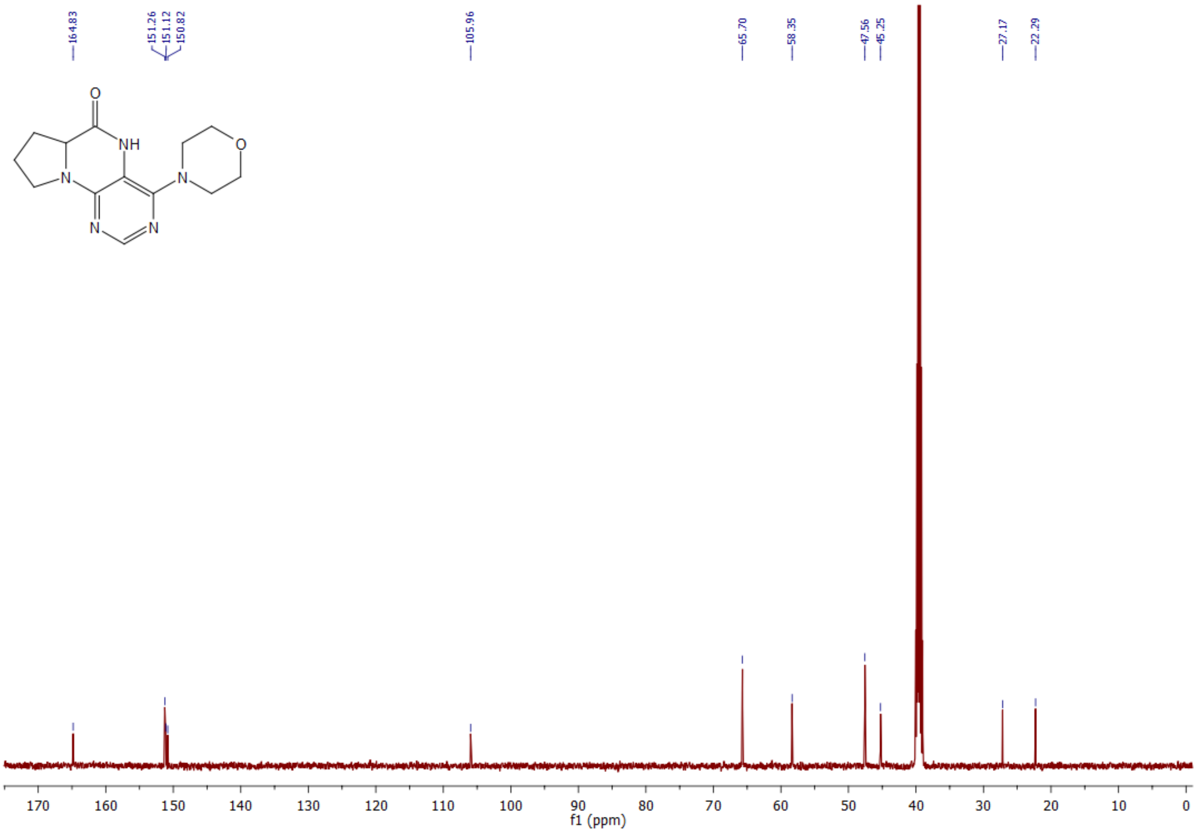

¹H and ¹³C NMR spectra of 4-(Propylamino)-5,7,8,9-tetrahydro-6H-pyrimido[4,5-b][1,4]diazepin-6-one (3a)

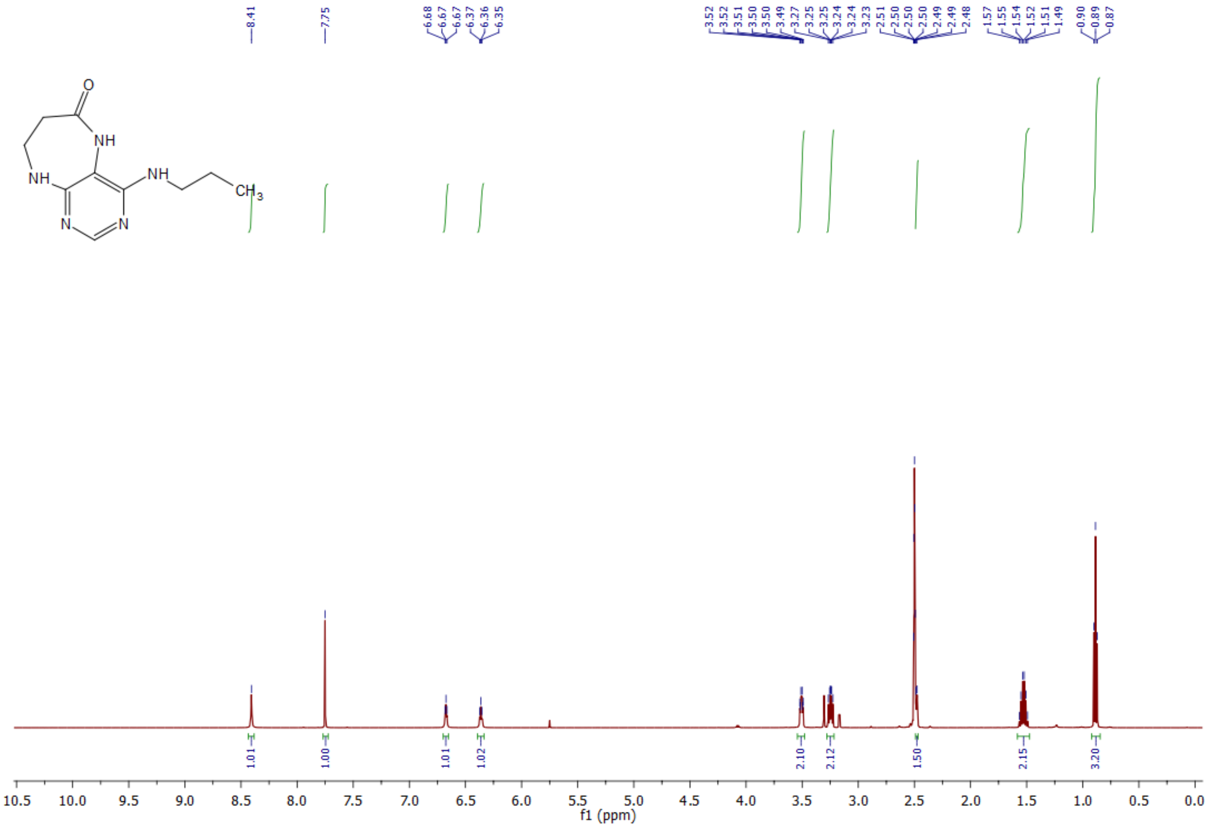

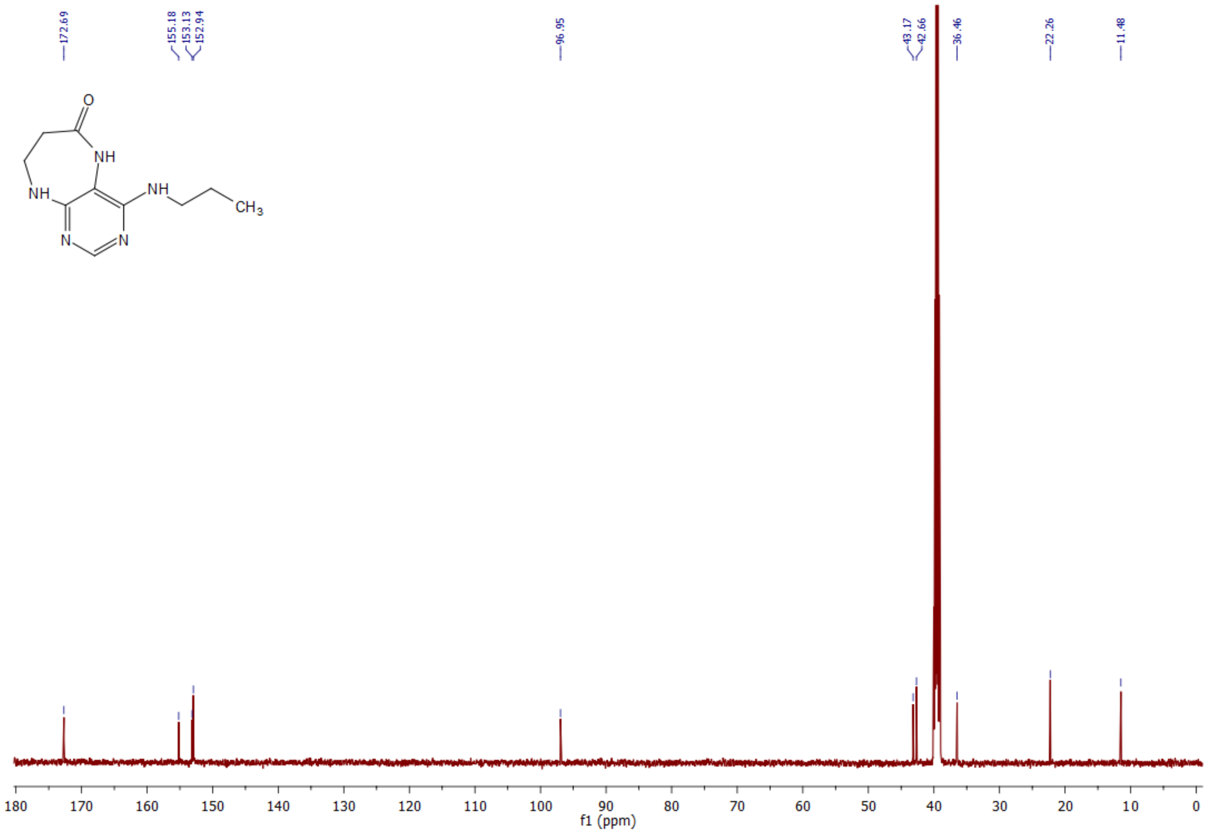

^{1}H and ^{13}C NMR spectra of 4-(Hexylamino)-5,7,8,9-tetrahydro-6H-pyrimido[4,5-b][1,4]diazepin-6-one (3b)

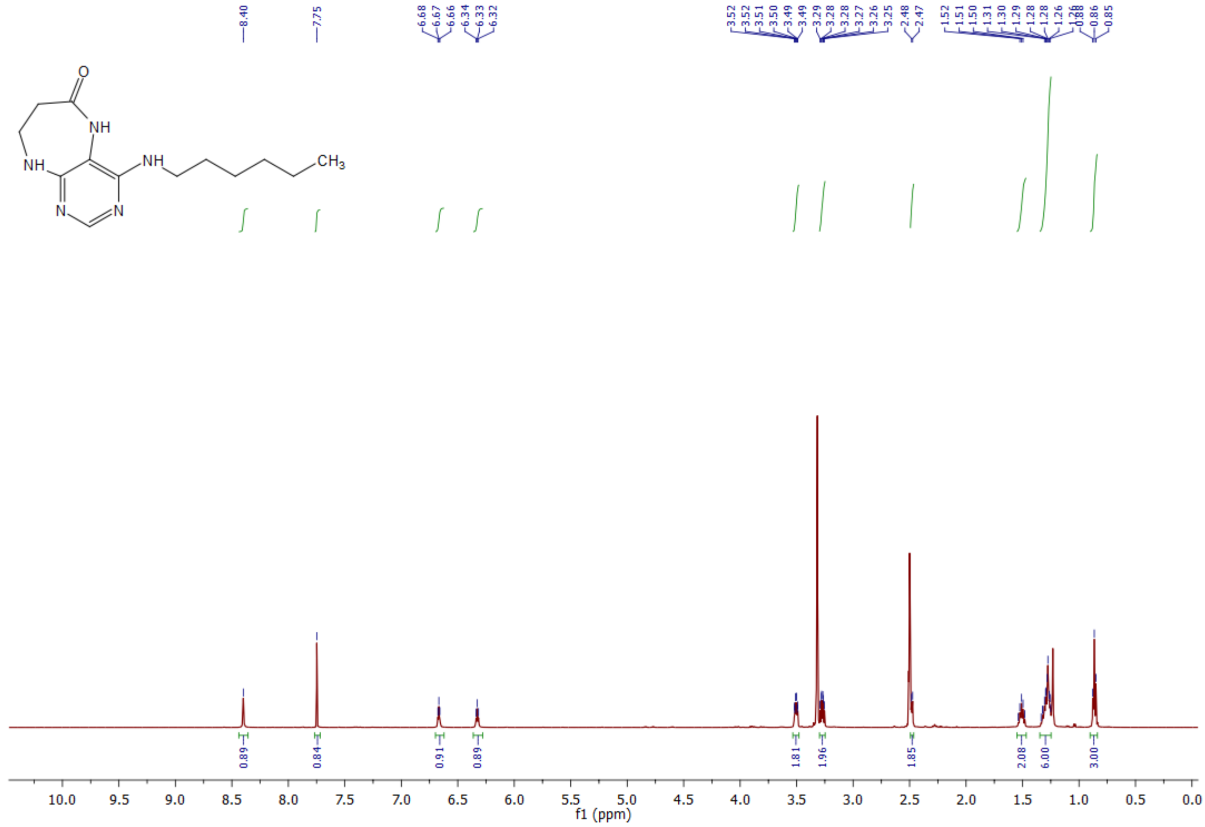

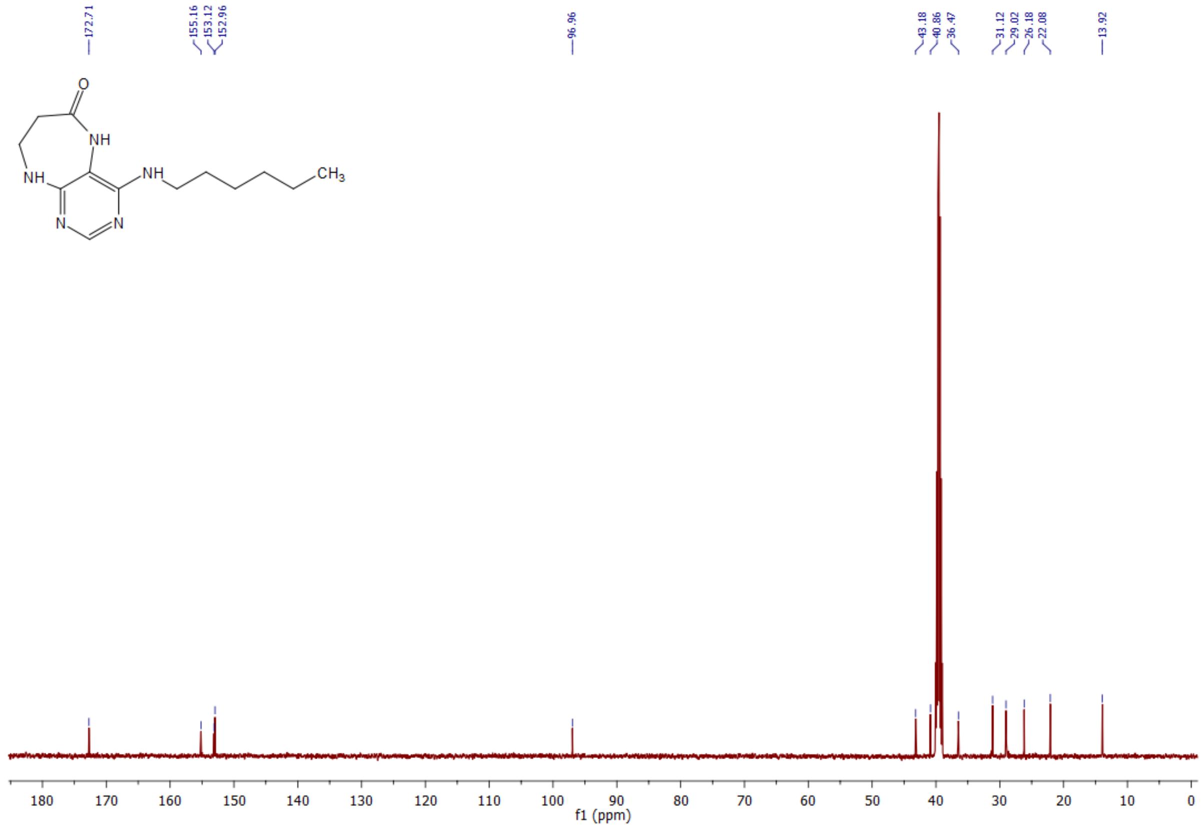

^{1}H and ^{13}C NMR spectra of 4-(Diethylamino)-5,7,8,9-tetrahydro-6H-pyrimido[4,5-b][1,4]diazepin-6-one (3c)

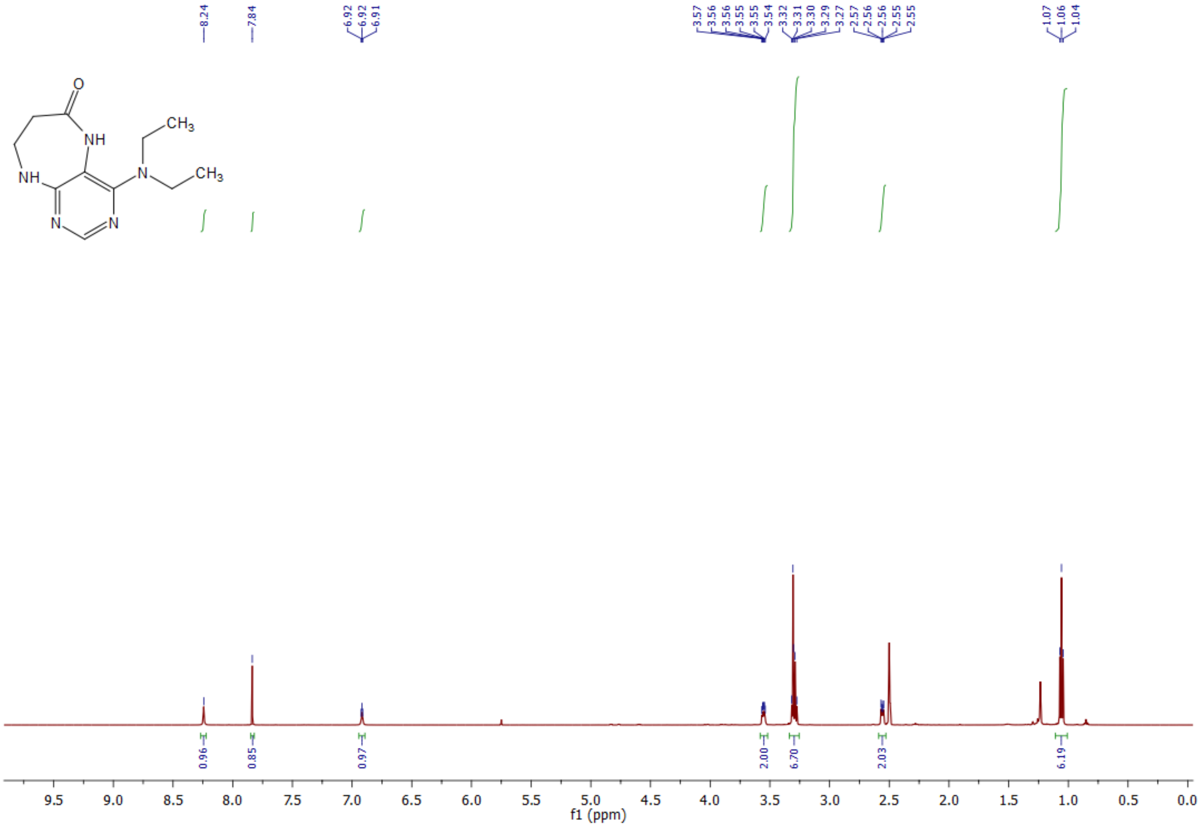

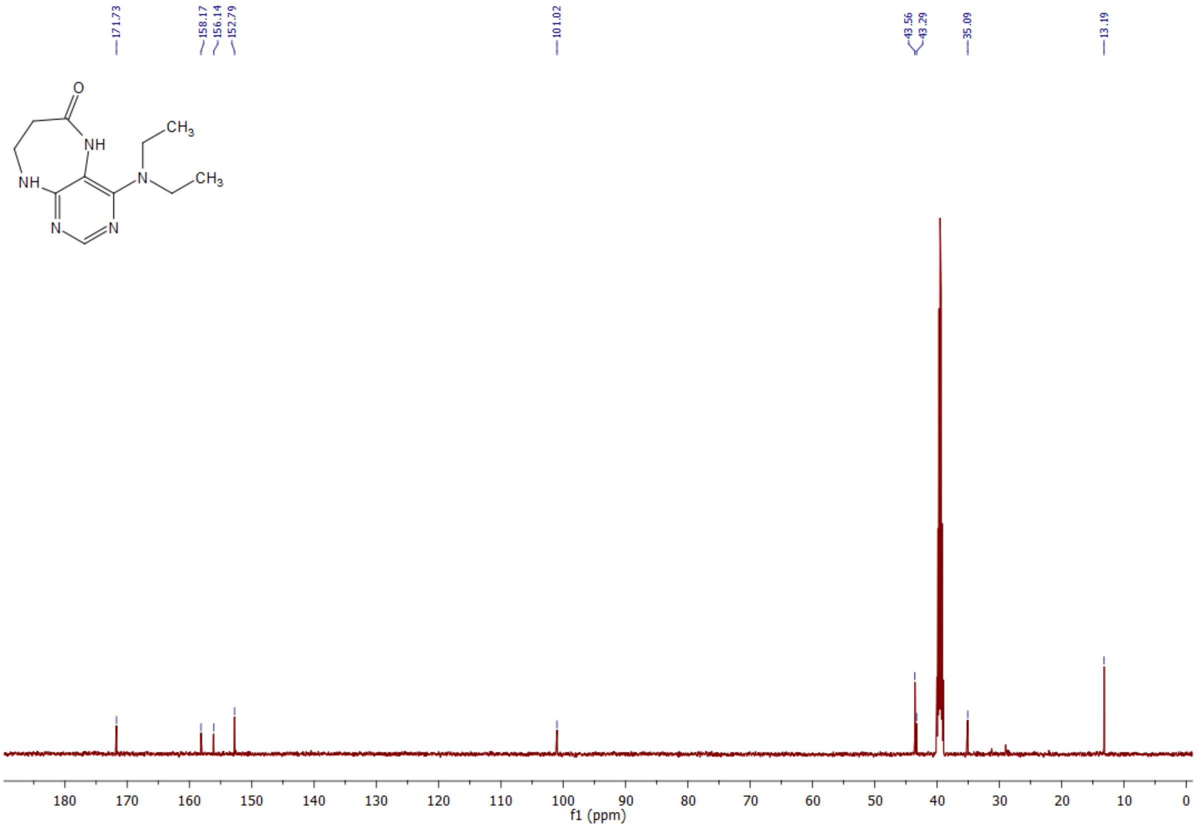

^{1}H and ^{13}C NMR spectra of 4-(Benzylamino)-5,7,8,9-tetrahydro-6H-pyrimido[4,5-b][1,4]diazepin-6-one (3d)

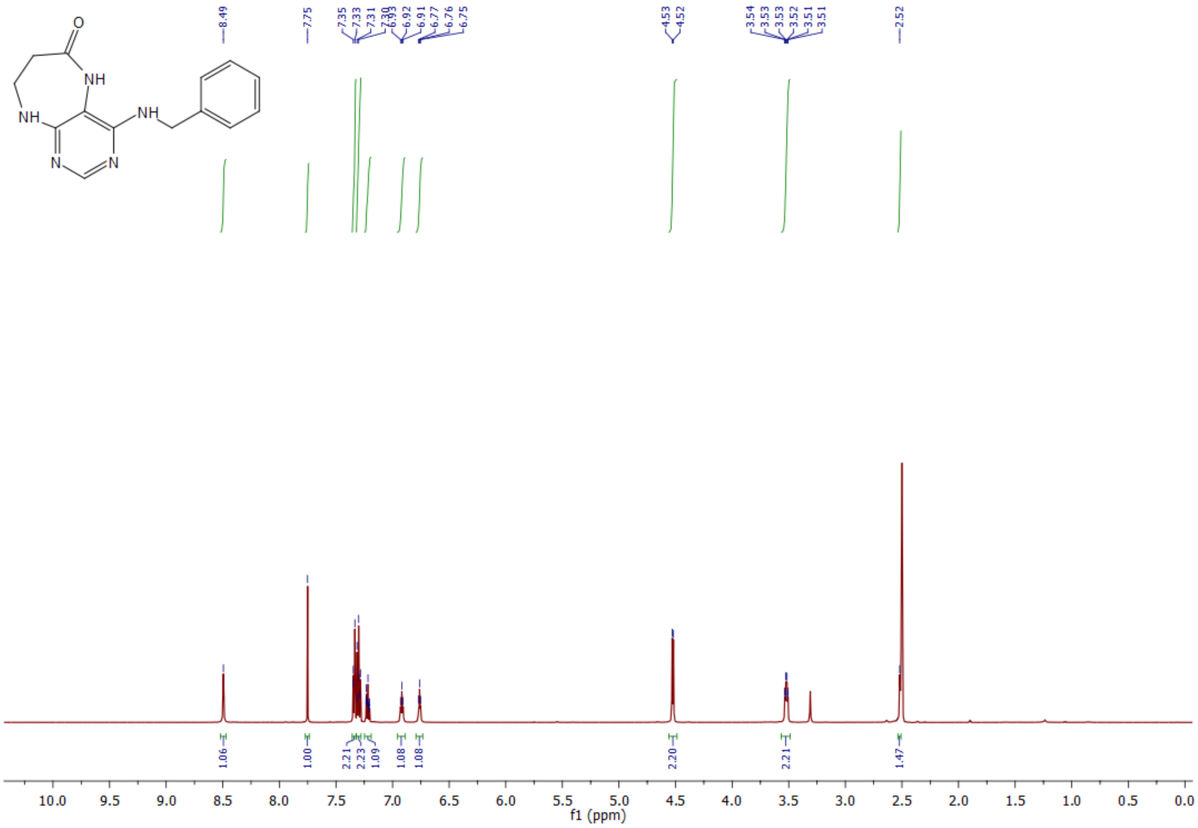

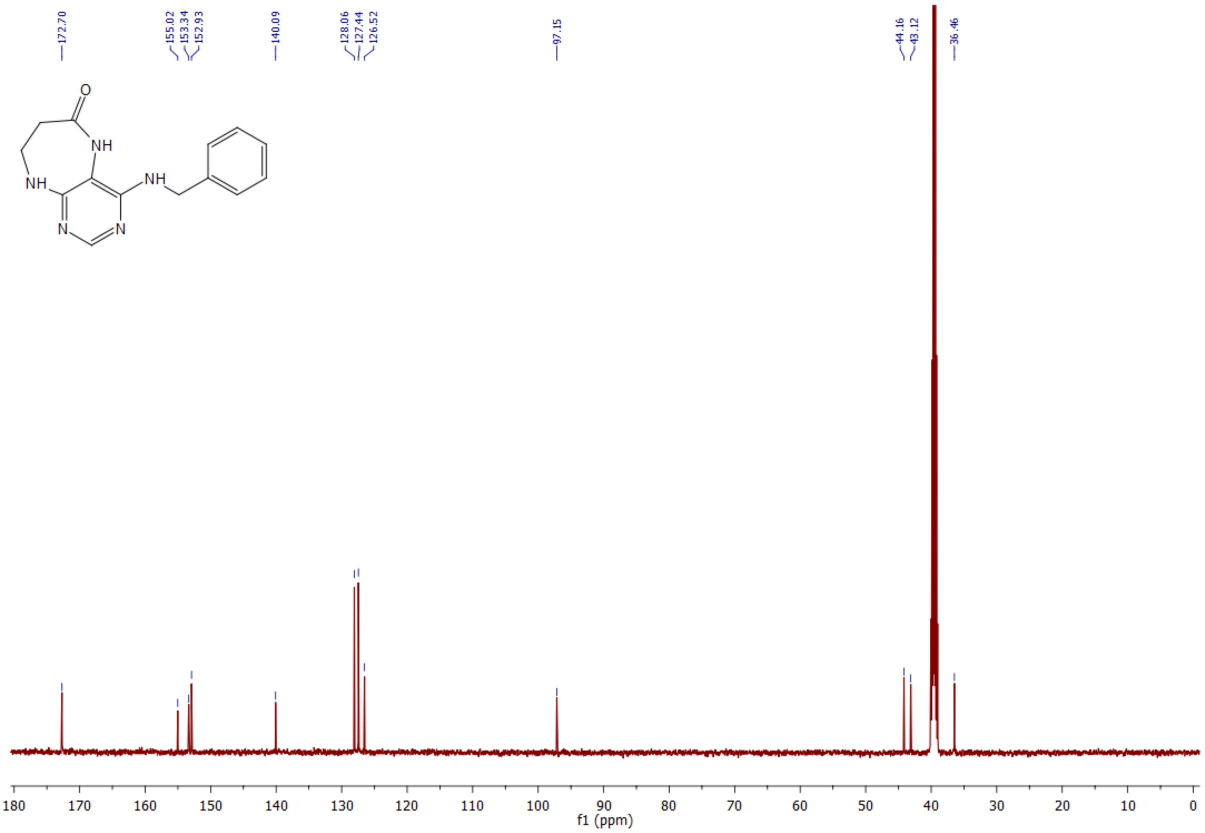

^{1}H and ^{13}C NMR spectra of 4-(Cyclohexylamino)-5,7,8,9-tetrahydro-6H-pyrimido[4,5-b][1,4]diazepin-6-one (3e)

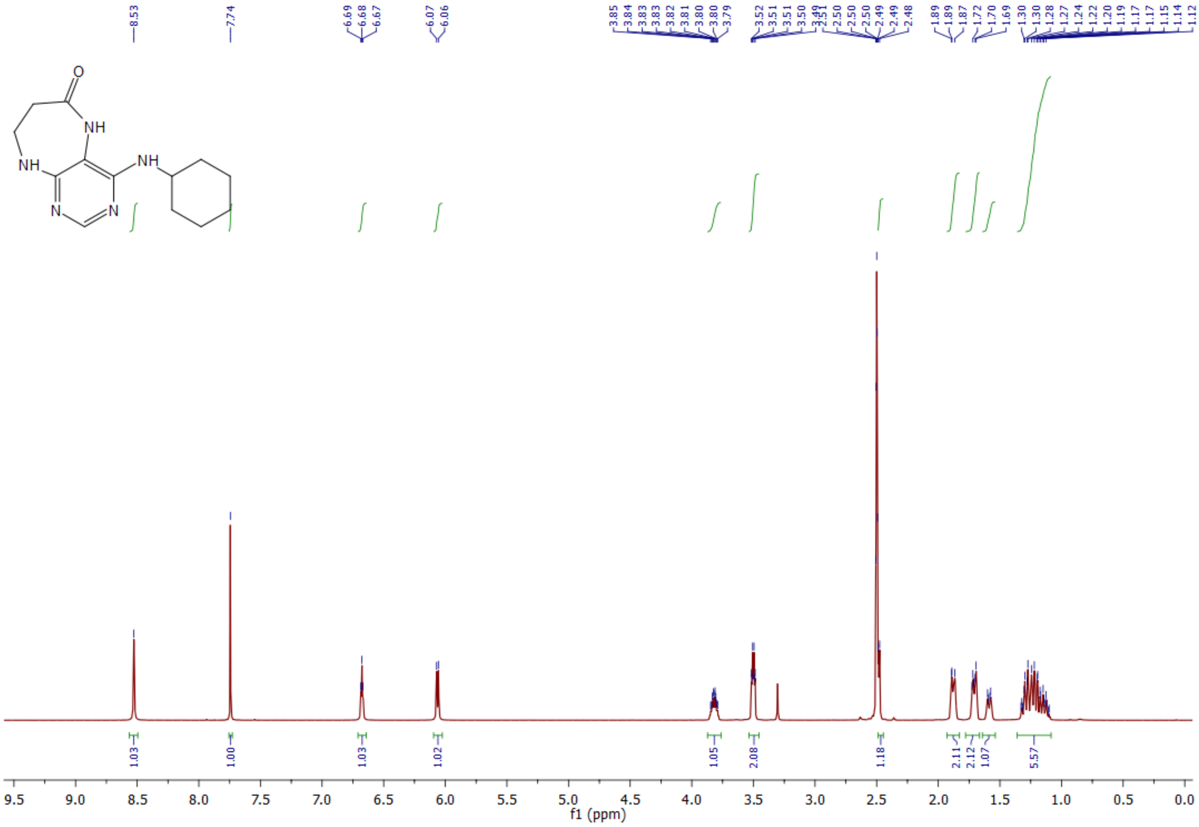

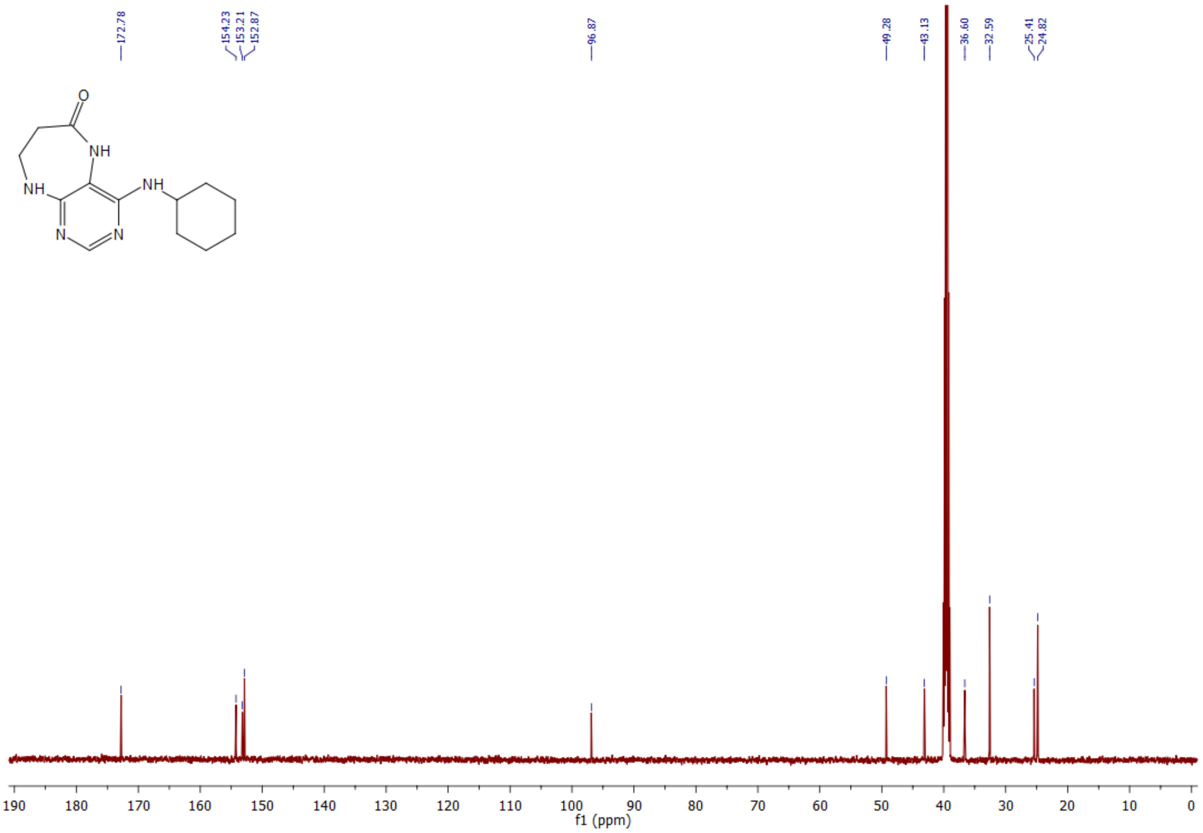

^{1}H and ^{13}C NMR spectra of 4-(Cyclooctylamino)-5,7,8,9-tetrahydro-6H-pyrimido[4,5-b][1,4]diazepin-6-one (3f)

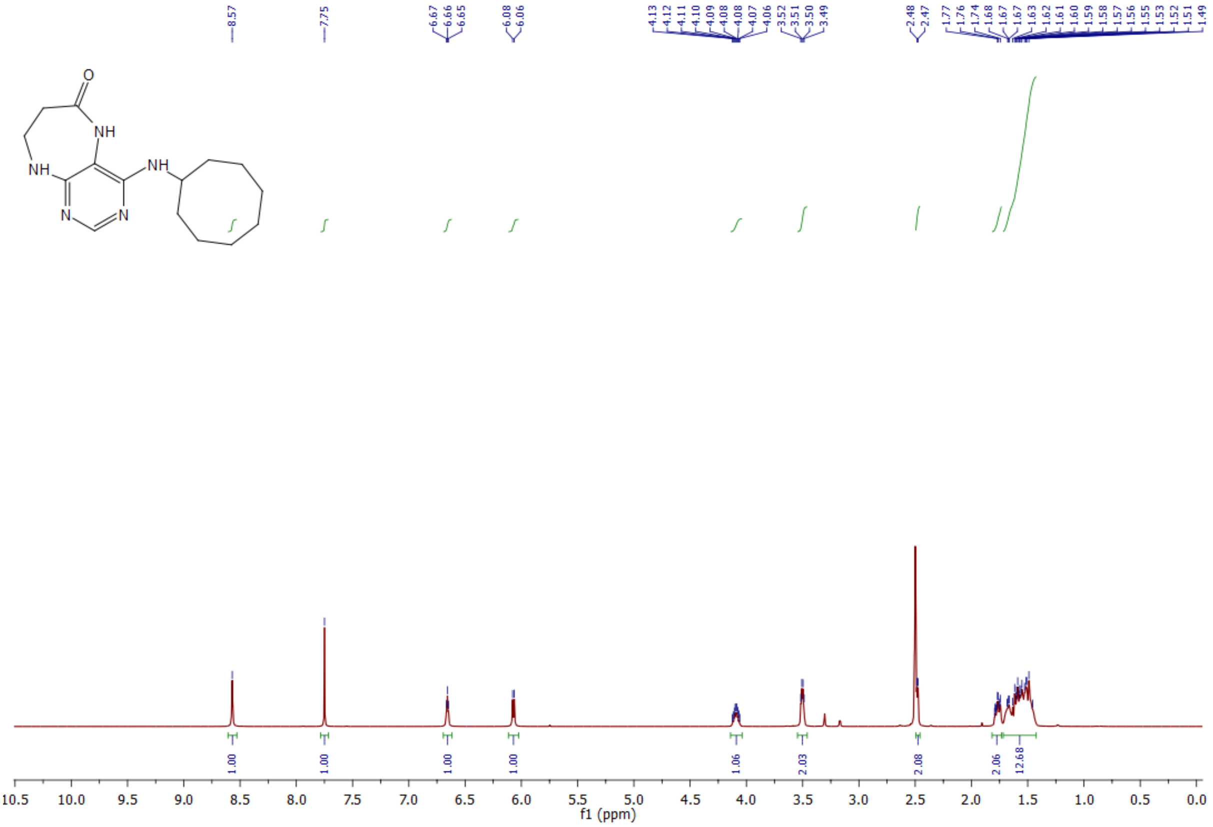

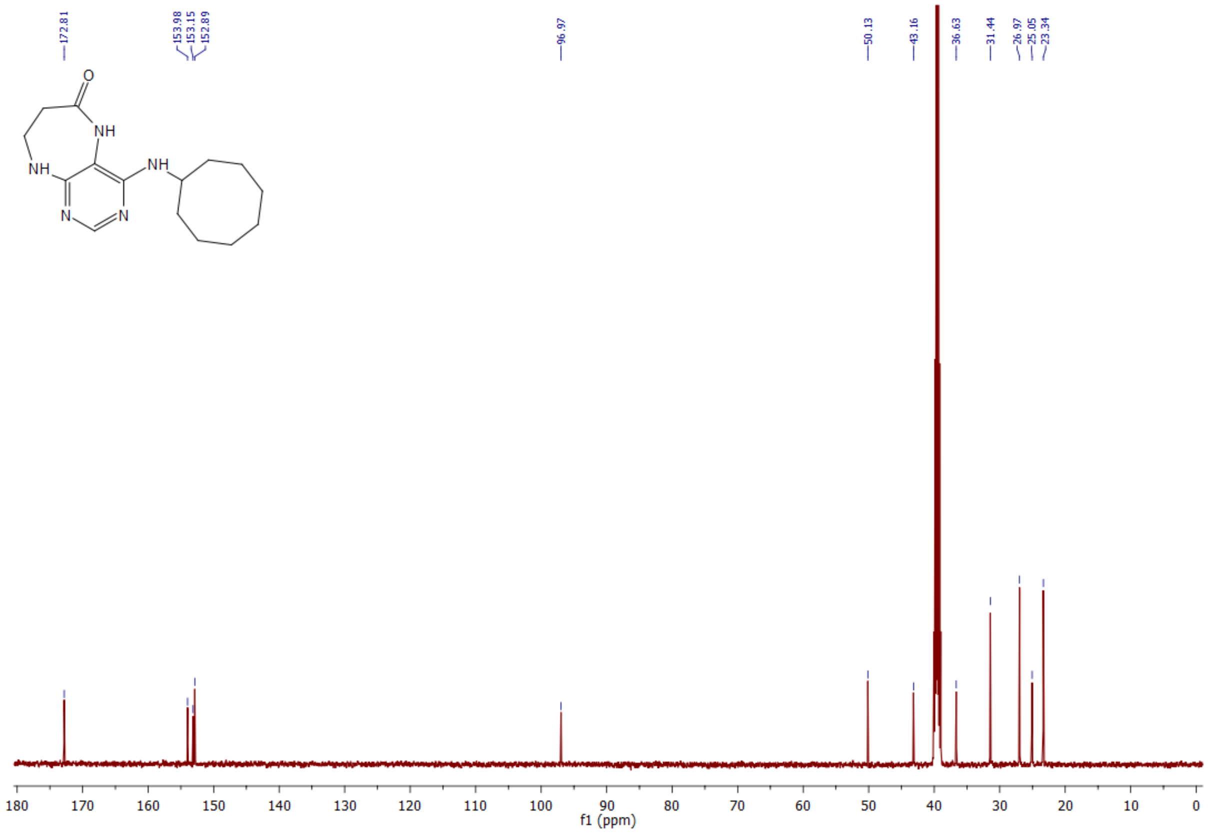

^{1}H and ^{13}C NMR spectra of 4-(Piperidin-1-yl)-5,7,8,9-tetrahydro-6H-pyrimido[4,5-b][1,4]diazepin-6-one (3g)

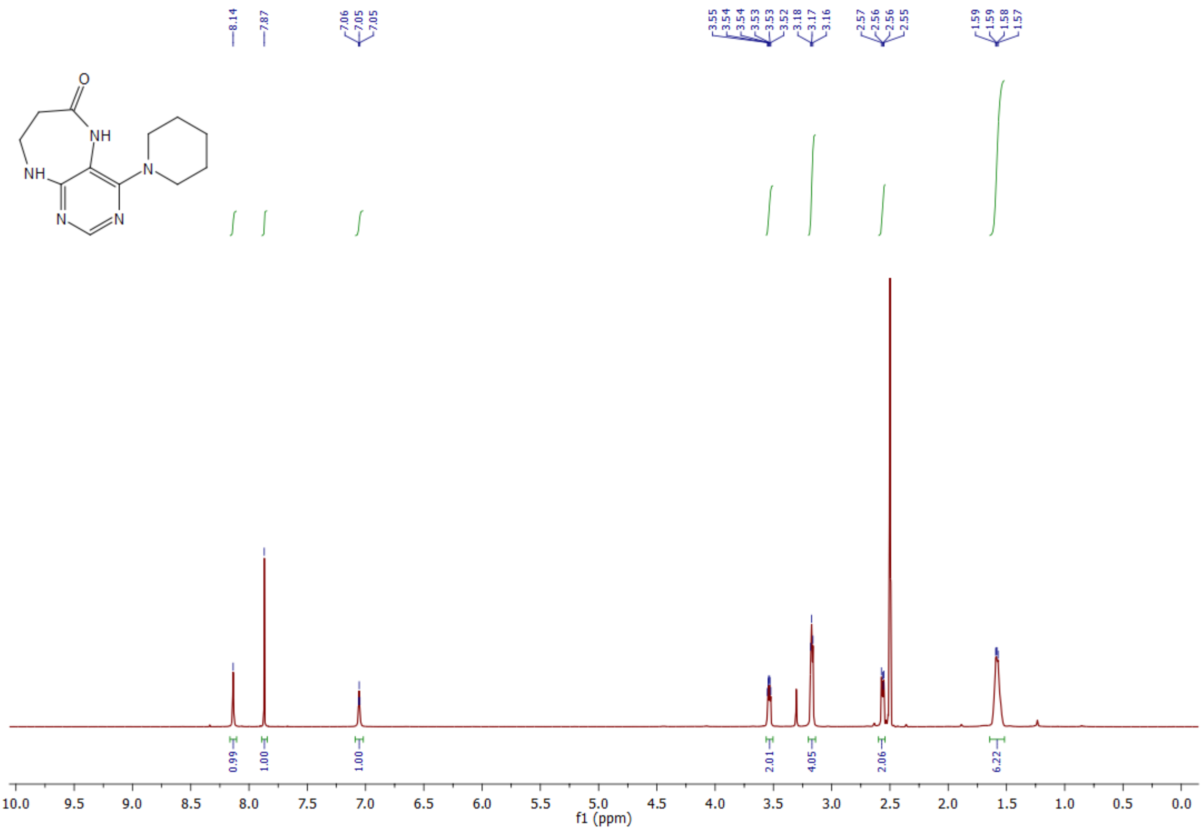

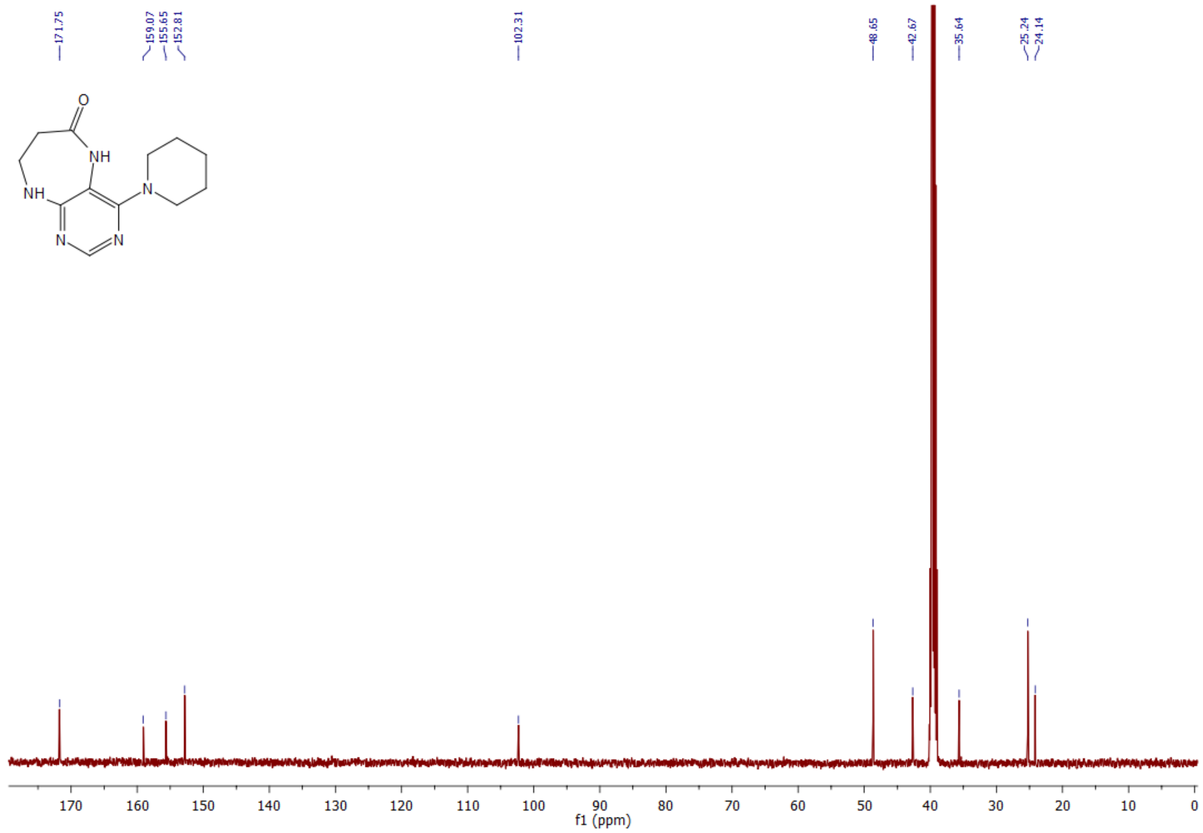

^{1}H and ^{13}C NMR spectra of 4-Morpholino-5,7,8,9-tetrahydro-6H-pyrimido[4,5-b][1,4]diazepin-6-one (3h)

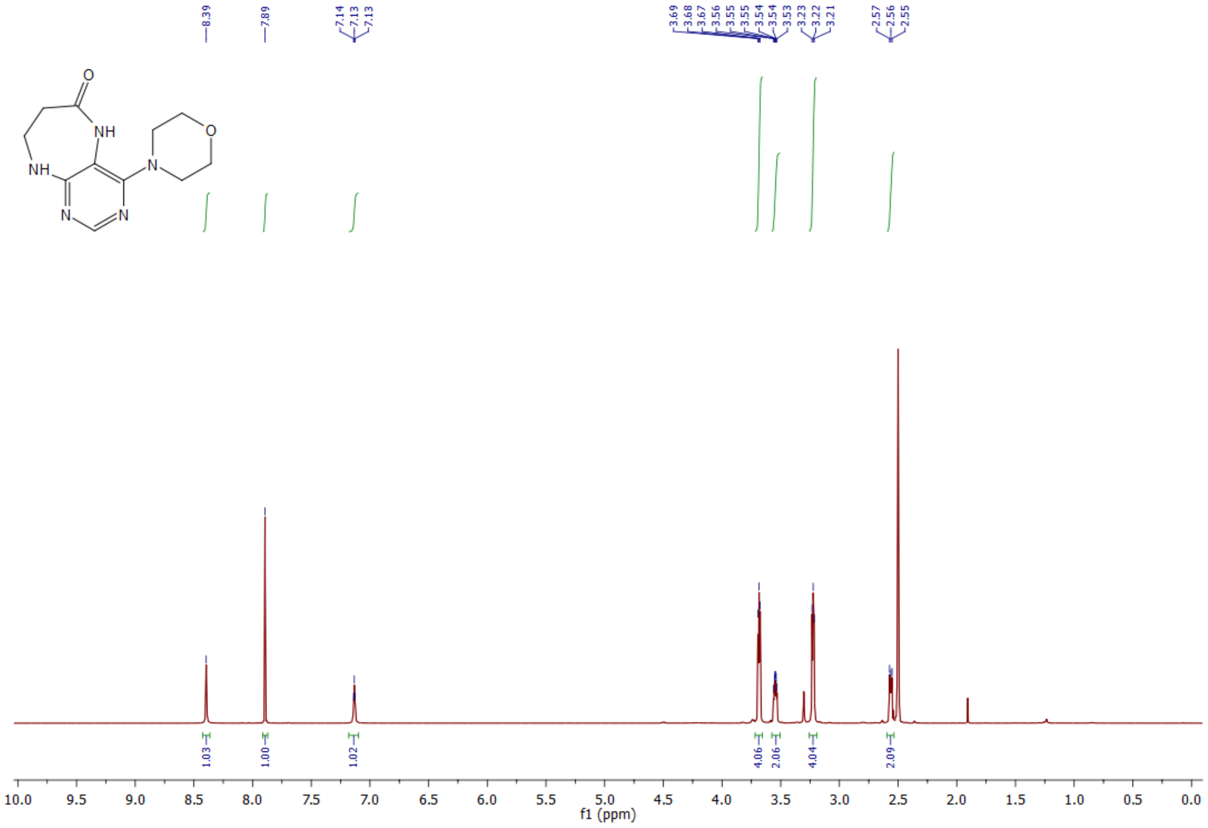

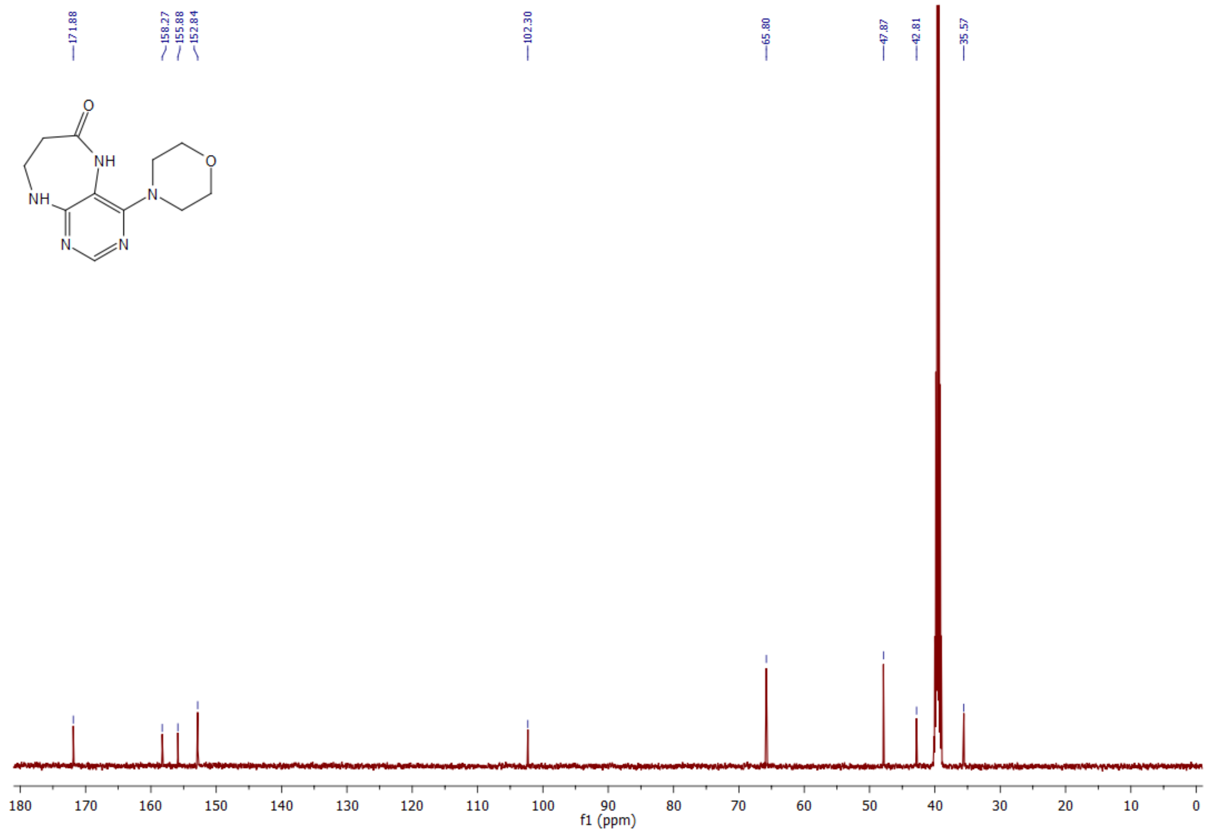

Supplement: Supplementary file 1 [file molecules-26-01603-s001.pdf]
